# *N*-Heterocyclic Carbene-Catalyzed Facile Synthesis of Phthalidyl Sulfonohydrazones: Density Functional Theory Mechanistic Insights and Docking Interactions

**DOI:** 10.1021/acsomega.3c08529

**Published:** 2024-02-26

**Authors:** Tanmoy Ghosh, Debabrata Barman, Krishanu Show, Rabindranath Lo, Debashree Manna, Tapas Ghosh, Dilip K. Maiti

**Affiliations:** † Department of Chemistry, University of Calcutta, Kolkata 700009, India; ‡ Department of Chemistry, Malda College, Malda, West Bengal 732101, India; § Institute of Organic Chemistry and Biochemistry, Czech Academy of Sciences, v.v.i., Flemingovo nám. 2, Prague 6, Praha 16610, Czech Republic; ∥ Department of Applied Chemistry, Maulana Abul Kalam Azad University of Technology, Haringhata, West Bengal 741249, India; ⊥ Department of Chemistry, Jadavpur University, Kolkata 700032, India

## Abstract

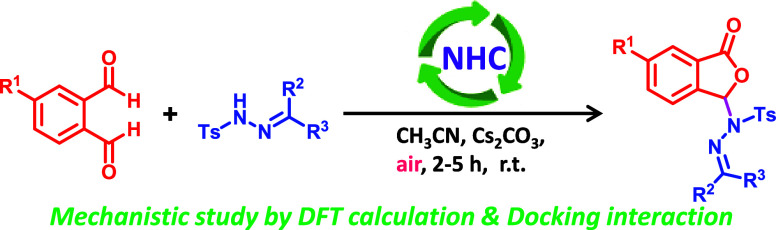

*N*-heterocyclic carbene catalysis reaction protocol is disclosed for the synthesis of phthalidyl sulfonohydrazones. A broad range of *N*-tosyl hydrazones react effectively with phthalaldehyde derivatives under open-air conditions, enabling the formation of a new C–N bond via an oxidative path. The reaction proceeds under mild reaction conditions with broad substrate scopes, wide functional group tolerance, and good to excellent yields. The mechanistic pathway is studied successfully using control experiments, competitive reactions, ESI-MS spectral analyses of the reaction mixture, and computational study by density functional theory. The potential use of one of the phthalidyl sulfonohydrazone derivatives as the inhibitor of β-ketoacyl acyl carrier protein synthase I of *Escherichia coli* is investigated using molecular docking.

## Introduction

**Figure 1:**
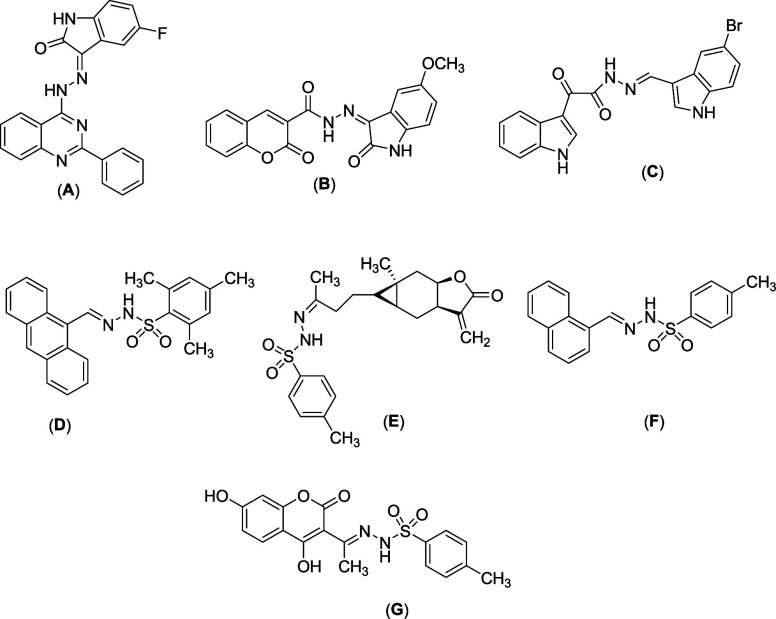
Few drugs and bioactive molecules (A–G) containing hydrazone and tosyl hydrazone units.

Chemical modification on medicinally important natural products or drug molecules is an established strategy to develop chemical entities and prodrugs with enhanced performance or introducing different clinical applications. Sulfonamide-based organic molecules find profound applications in the field of medicine and pharmaceutical areas.^1^ Hydrazones are a significant class of organic molecules for drug design and synthesis. Of late, these functional compounds have been developed with numerous biological activities. Some hydrazone derivatives have proven to possess antimicrobial,^2^ antimalarial,^3^ antiviral,^4^ vasodilator,^5a^ anti-inflammatory,^5b^ anticancer,^5c^ and antituberculosis^6^ activities (**A–C**, Figure 1). Tosyl hydrazone compounds are found as antibacterial, antifungal, and anticancer agents (**D–G**, Figure 1).^7^ Moreover, tosyl hydrazones are treated as versatile and useful partners in organic synthesis.^8^ In particular, under basic conditions, the tosyl hydrazone moieties are easily converted into diazo compounds,^9^ which can undergo insertion reactions, leading to the construction of various chemical bonds like C–C, C–N, C–Si, etc. In addition, hemiaminal esters constitute crucial building blocks for several biological systems. In general, these compounds are obtained via a dynamic kinetic resolution process using various substrates.^10^ Among the various types of sulfonamides, phthalidyl sulfonamide promoters were found with impressive success. Phthalidyl sulfonamides derivatives are an important class of heterocycles having potent biological and photophysical properties. For instance, hydrochlorothiazide is a diuretic medication for the treatments of high blood pressure, congestive heart failure, diabetes insipidus, and renal tubular acidosis.^11^ Saccharin is an artificial sweetener and food additive. Sulfamethoxazoles are known as antibiotics used for bacterial infections such as urinary tract infections, bronchitis, and prostatitis.^12^

**Scheme 1:**
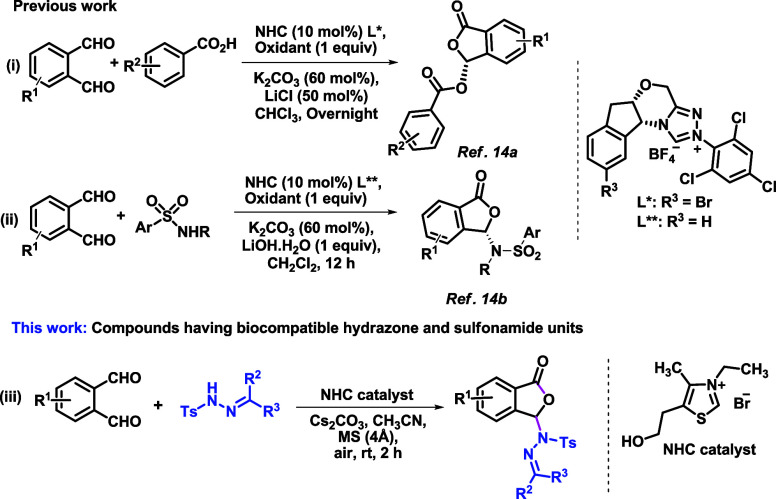
Synthesis of Phthalidyl Esters (eq i), Sulfonamides (eq ii), and Our Approach (eq iii) to Phthalidyl Sulfonohydrazones

Sustainable *N*-heterocyclic carbene (NHC) organocatalysis is established as a powerful tool in modern organic synthesis to access functional molecules.^13^ Chi and co-workers reported an enantioselective method for the synthesis of chiral phthalidyl ester via NHC-catalyzed acetalization of carboxylic acids using a stoichiometric amount of oxidant (eq i, Scheme 1).^14a^ Very recently, the same group has disclosed the reaction of *N*-aryl sulfonamides with phthalaldehydes producing optically enriched phthalidyl sulfonamides under NHC organocatalysis and subsequent oxidation (eq ii, Scheme 1). However, the reaction did not proceed without the use of 3,3′,5,5′-tetra-*tert*-butyldiphenoquinone as an externally supplied oxidant (1 equiv).^14b^ Aerial oxidation in NHC catalysis is well documented in the literature to access aromatic esters or carboxylic acids from aromatic aldehydes with alcohols or nonactivated aldehydes, respectively.^15^ Thus, we examined the NHC-catalyzed C–O/C–N coupled reaction using molecular oxygen from air (eq iii, Scheme 1).

Inspired by the very interesting results, we assume that the sulfonohydrazide moiety-containing phthalidyl scaffold could be an appropriate modification of existing phthalidyl sulfonamide derivatives, which were found having diverse bioactivity. To the best of our knowledge, until date, there is no report regarding the bioactivity assay of phthalidyl sulfonohydrazone derivatives, as their synthesis is unknown in the literature. Our aim was to synthesize organic molecules, which in turn looked like as sulfonamides, hemiaminal ester, and phthalidyl sulfonohydrazone. In this context, we disclosed NHC access to bioactive phthalidyl sulfonohydrazone from phthalaldehyde and *N*-tosyl hydrazones in good to excellent yield under aerobic oxidation (eq iii, Scheme 1). The mechanistic pathways are explored in detail using the density functional theory (DFT) computational study. Further, molecular docking is performed to see the potential bioactivity of one of the reported compounds.

## Results and Discussion

At the outset of our studies, we have investigated the reaction employing *N*-tosyl hydrazone derivative (**2b**) and phthalaldehyde (**1a**) as the model substrates (Table 1) with the variation of catalytic amounts of various NHCs in open air. The oxidative NHC-catalyzed reaction was initially attempted using imidazolium salt **3a** or thiazolium salt **3b** along with various bases and solvents (entries 1–9, Table 1). These reactions were either unsuccessful or only a trace of the desired product (**4b**) was obtained in these cases. Gratifyingly, the desired product was obtained in 55% yield on the treatment of the substrate with thiazolium salt **3c** and base DBU in dichloromethane solvent for 12 h at ambient temperature (entry 10). To our delight, the yield (86%) and reaction rate (the reaction time decreased to 2 h) were drastically improved on the use of Cs_2_CO_3_ as a base in acetonitrile (entry 11). The yield of the **4b** was not improved at all on changing the reaction medium and base (entries 12–15). On the enhancement of catalyst loading (12 mol %) and reaction time (6 h), the yield was not significantly improved (88%, entry 16). A decrease of catalyst loading (8 and 6%, respectively) led to the lowering of the reaction rate and yield (entries 17 and 18). The reaction did not proceed in the absence of NHC (entry 19). Thus, we found the optimized conditions for this reaction using Cs_2_CO_3_ as the base in acetonitrile solvent with NHC **3c** as the efficient catalyst in air to produce the desired phthalidyl sulfonohydrazone derivative in 2 h with 86% yield (entry 11). Also, we have performed the reaction under inert atmosphere conditions using “kahrasch oxidant” (3,3′,5,5′-tetra-*tert*-butyldiphenoquinone, DPQ) and observed a moderate yield.^14b^

**Table 1:**
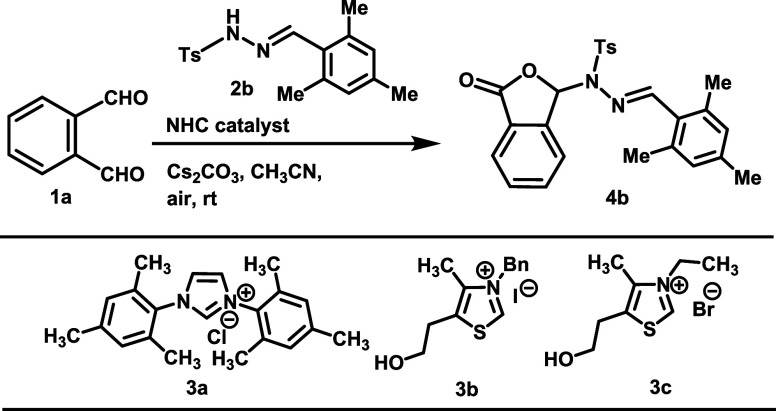
Screening for Optimized Reaction Conditions

entry^a^	catalyst	base	solvent^b^	time (h)	yield (%)^c^
1	3a	DBU	DCM	12	nd^d^
2	3a	DBU	DMF	12	nd
3	3a	Cs_2_CO_3_	DCM	12	nd
4	3a	DBU	THF	12	nd
5	3a	NaOAc	THF	12	nd
6	3b	Cs_2_CO_3_	THF:EtOH(1:1)	12	10
7	3b	Cs_2_CO_3_	DCM	12	<5
8	3b	DBU	THF	12	trace
9	3b	NaOAc	THF:EtOH(1:1)	12	trace
10	3c	DBU	DCM	12	55
11	3c	Cs_2_CO_3_	CH_3_CN	2	86
12	3c	Cs_2_CO_3_	DMF	12	42
13	3c	Cs_2_CO_3_	THF	12	65
14	3c	Cs_2_CO_3_	THF:EtOH(1:1)	12	15
15	3c	NaOAc	EtOH	12	10
16^d^	3c	Cs_2_CO_3_	CH_3_CN	6	86
17^e^	3c	Cs_2_CO_3_	CH_3_CN	8	70
18^f^	3c	Cs_2_CO_3_	CH_3_CN	12	40
19^g^		Cs_2_CO_3_	CH_3_CN	12	nd
20^h^	3c	Cs_2_CO_3_	CH_3_CN	8	84

a Reaction conditions: phthalaldehyde (**1**, 1.0 mmol), *N*-tosyl hydrazone (**2**, 1.0 mmol), solvent (5 mL), NHC precursor **3a**–**c** (10 mol %), and base (20 mol %) were stirred at ambient temperature.

b Molecular sieves (4 Å) and air used.

c Yield of the product obtained after purification by silica gel column chromatography.

d **3c**: 12 mol %.

e **3c**: 8 mol %.

f **3c**: 6 mol %.

g Without NHC, nd: **4** not detected.

h Gram-scale synthesis.

**Scheme 2:**
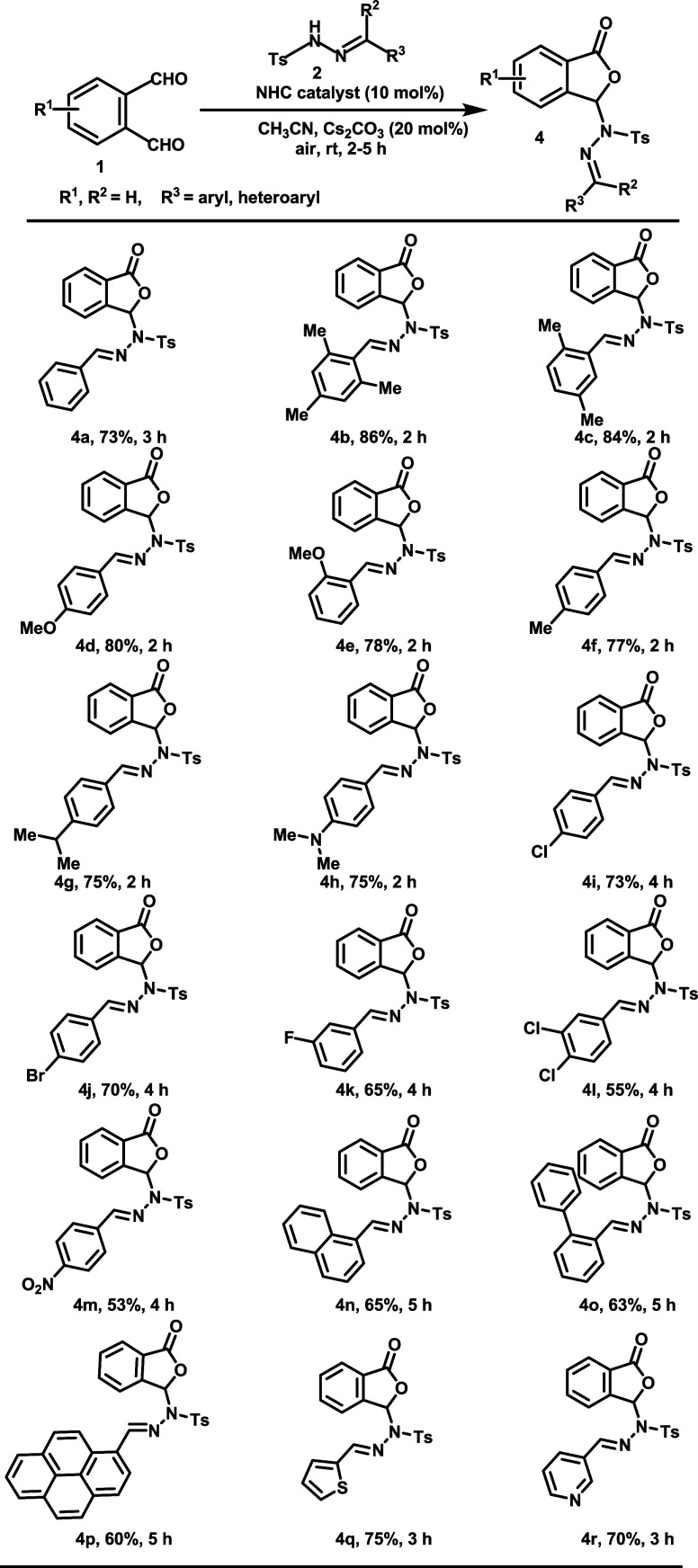
Synthesized Phthalidyl Sulfonohydrazones (**4**) from *N*-Tosyl Hydrazones

General applicability of the developed reaction conditions (entry 11, Table 1) using various substituted *N*-tosyl hydrazones (**2**) and phthalaldehyde (**1**) to obtain functionalized phthalidyl sulfonohydrazide moieties (**4a**–**s**) was framed in Scheme 2. *N*-tosyl hydrazones were derived from aryl aldehydes in this case. Electron-donating groups in the aromatic ring of *N*-tosyl hydrazones performed well under these optimized reaction conditions to yield the product (**4b**–**h**) in 75–86%. Moderate to good yields (53–73%) were observed for *N*-tosyl hydrazones having an electron-withdrawing halogen or nitro group (**4i**–**m**). The reaction also went well when sterically hindered aldehyde precursors from naphthalene, biphenyl, and pyrene were tested under the reaction conditions to furnish desired products (**4n**–**p**) in 65, 63, and 60% yields, respectively. The reaction is also in consistent with heterocyclic *N*-tosyl hydrazones (**4q**, **r**). *N*-tosyl hydrazine from cinnamaldehyde also tolerated in this reaction protocol to furnish corresponding phthalidyl sulfonohydrazide **4s** in 68% yield. In general, all of these allowed installation of a great diversity of substituents in the sulfonohydrazone template.

**Scheme 3:**
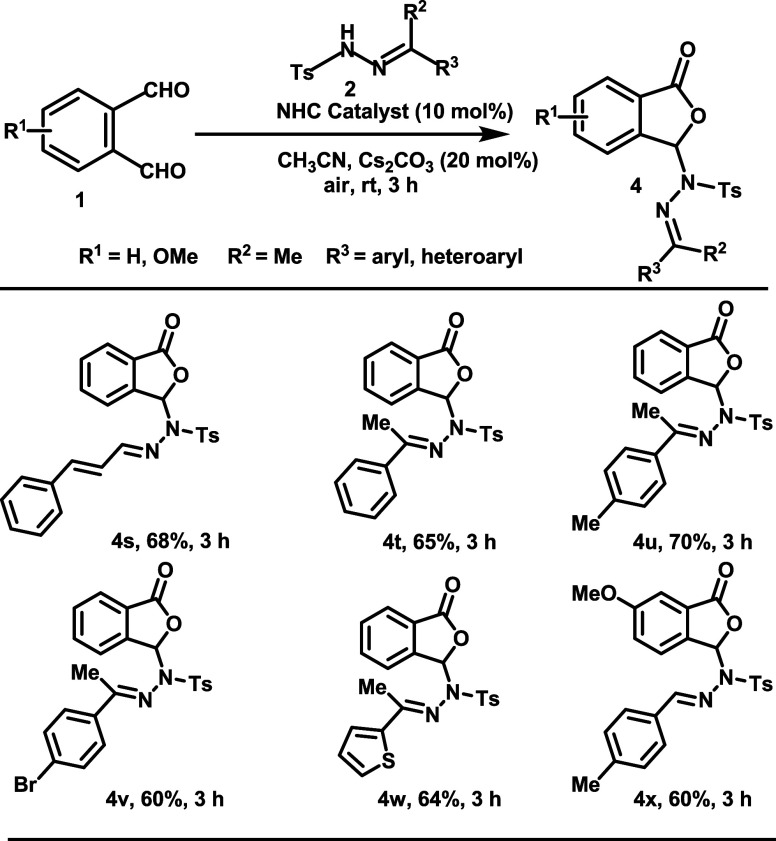
Synthesized Phthalidyl Sulfonohydrazones (**4**) from Ketohydrazones

We further studied the *N*-tosyl hydrazones derived from aryl ketones as substrates to react with phthalaldehydes under the optimized reaction conditions (Scheme 3). *N*-tosyl hydrazones were well tolerated in the reaction protocol irrespective of the major electronic effects of the substituents in the aryl part of the hydrazones and furnished the desired products **4t**–**v** in good yields (60–70%). *N*-tosyl hydrazones with a heteroaryl moiety in the structure reacted well and furnished the product (**4w**) in 64% yield. Regioselectivity studies of unsymmetrical phthalaldehyde were investigated, which afford **4x** as a single isomer with good yield (60%). A gram-scale synthesis of **4b** under the optimized reaction conditions was also verified to afford the desired product in 84% yield (entry 20). The structure of the all unknown compounds (**4a–x**) was determined unambiguously by recording NMR, FT-IR, and HRMS spectra and single-crystal XRD data analyses of compound **4m** (Supporting Information).

**Scheme 4:**
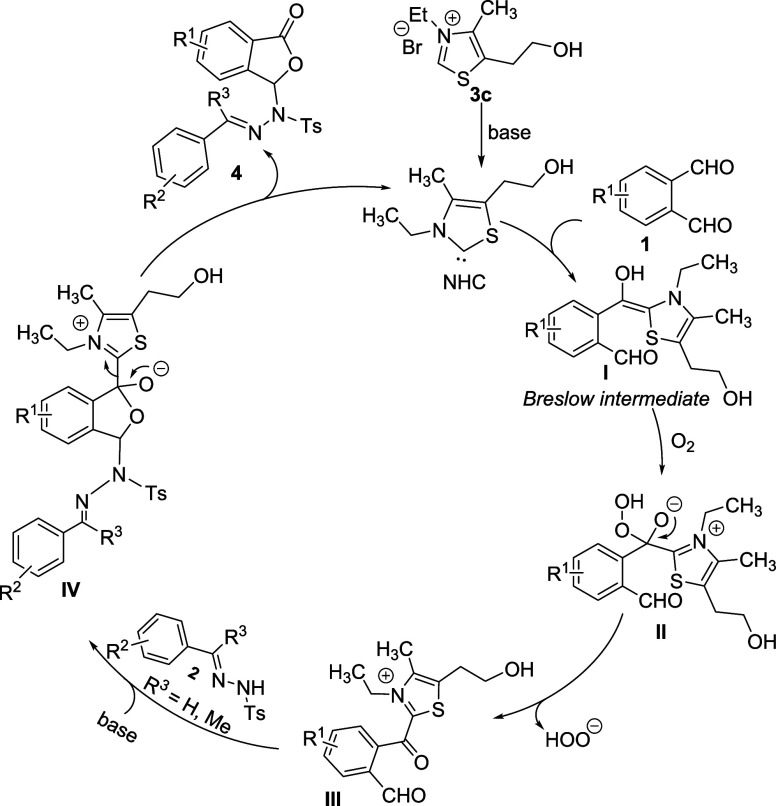
Plausible Mechanistic Pathway

To elucidate the reaction pathway, ESI-MS and DFT calculations were performed. We assume that a nucleophilic NHC-aldehyde adduct (Breslow intermediate) (**I**) is formed initially, which upon aerial oxidation generates the azolium hydroperoxy intermediate **II**(15d,15e) (Scheme 4). Intermediate **II** converts into the acyl azolium intermediate **III**, with liberation of the hydroperoxy anion (oxidative path). The intermediate **III** further undergoes nucleophilic attack by deprotonated *N*-tosyl hydrazone, followed by O–C coupling to give intermediate **IV** through intramolecular annulation.^14^ The intermediate **IV** upon fragmentation may yield a phthalidyl sulfonohydrazone as the final product along with the regeneration of the active catalyst. In the following sections, we will discuss the detailed reaction mechanisms, including the formation of the Breslow intermediate, aerial oxidation of the Breslow intermediate, nucleophilic attack of deprotonated *N*-tosyl hydrazone, O–C coupling, and dissociation of the NHC catalyst.

### DFT Study: Formation of the Breslow Intermediate

**Figure 2:**
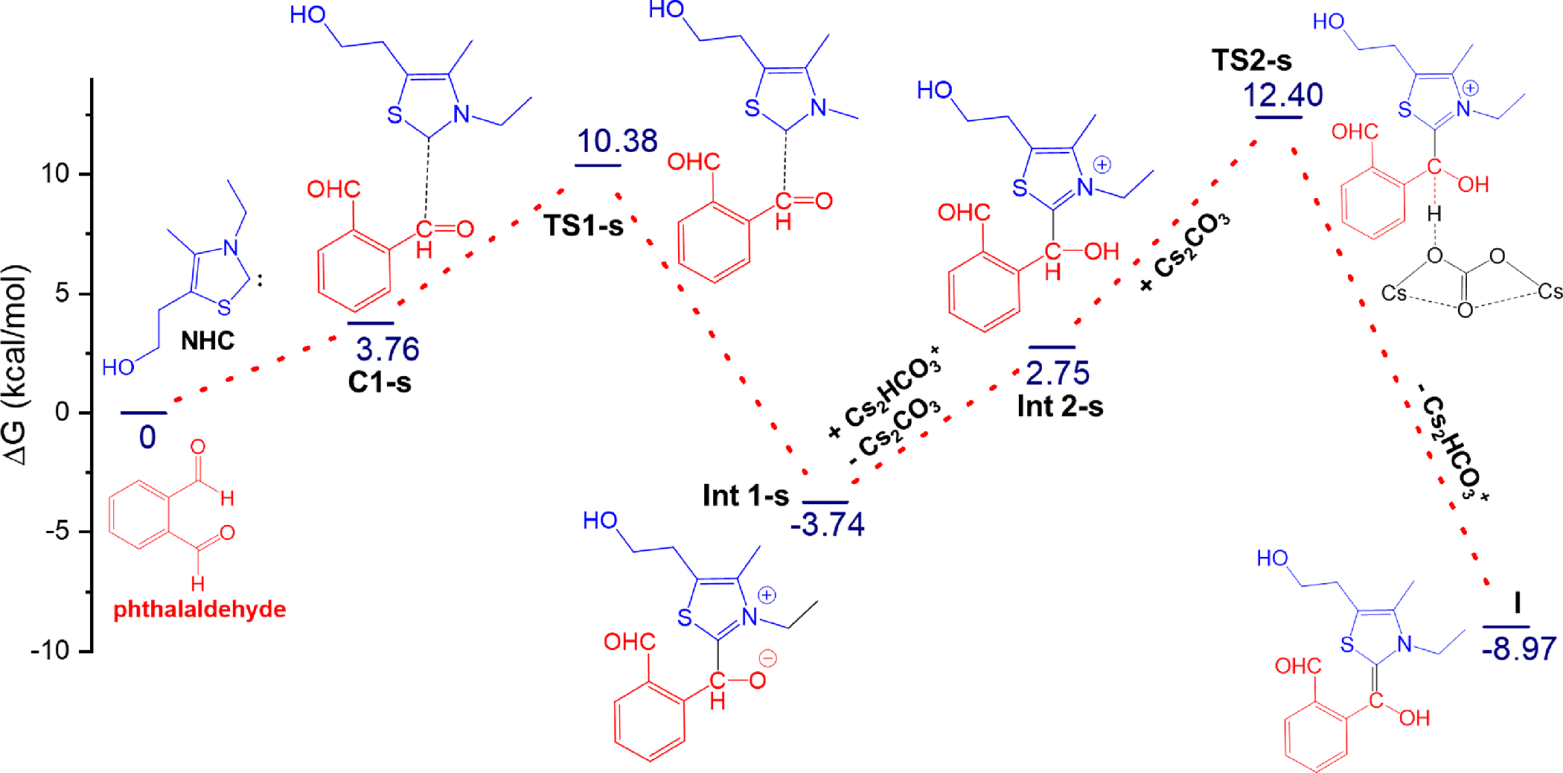
Free energy diagram for forming the Breslow intermediate (**I**) from NHC with phthalaldehyde.

The suggested mechanism involves the nucleophilic attack of NHC to the electrophilic aldehyde group of phthaladehyde, giving rise to the Breslow intermediate (Figure 2). At first, the reaction mixture leads to the formation of free NHC as a result of proton transfer from the azolium cation to the base, CS_2_CO_3_ (Figures S30 and S31, Supporting Information). The free energy profile shows that the formation of NHC occurs with a minimal activation barrier of 2.07 kcal/mol. Then, NHC generates a vdW complex (**C1-s**) by the nucleophilic attack to aldehyde. It is observed that the zwitterionic intermediate (**Int1-s**) occurs through the transition state (**TS1-s**) with a free energy barrier of 10.38 kcal/mol from separated reactants. The generated intermediate exhibits a decrease in a free energy of −3.74 kcal/mol compared to separated reactants, which suggests an exergonic reaction process. The solvent-phase geometries corresponding to the Breslow intermediate formation are given in Figure S32 (Supporting Information). At **TS1-s**, the distance between NHC carbene C and carbonyl C shortens from 3.333 Å (**C1-s**) to 2.282 Å (**TS1-s**), demonstrating the gradual formation of the C–C bond. Finally, the zwitterionic intermediate (**Int1-s**) is generated with a C–C bond distance of 1.523 Å. The Breslow intermediate is formed by 1,2-proton transfer in the zwitterionic intermediate. In our recent study, we have shown that direct proton transfer has a very high free activation energy as a consequence of strained TS and the proton transfer process proceeds through the acid/base pair catalyzed energetically favorable pathway.^16^ Here, Cs_2_CO_3_ helps the 1,2-proton transfer path and the reaction proceeds through the stepwise reaction. In this pathway, the protonation of the O atom occurs before the C–H cleavage takes place. On the other hand, the hydrogen abstraction has an activation free energy of 12.40 kcal/mol from the separated reactants.

### Aerial Oxidation of the Breslow Intermediate

**Figure 3:**
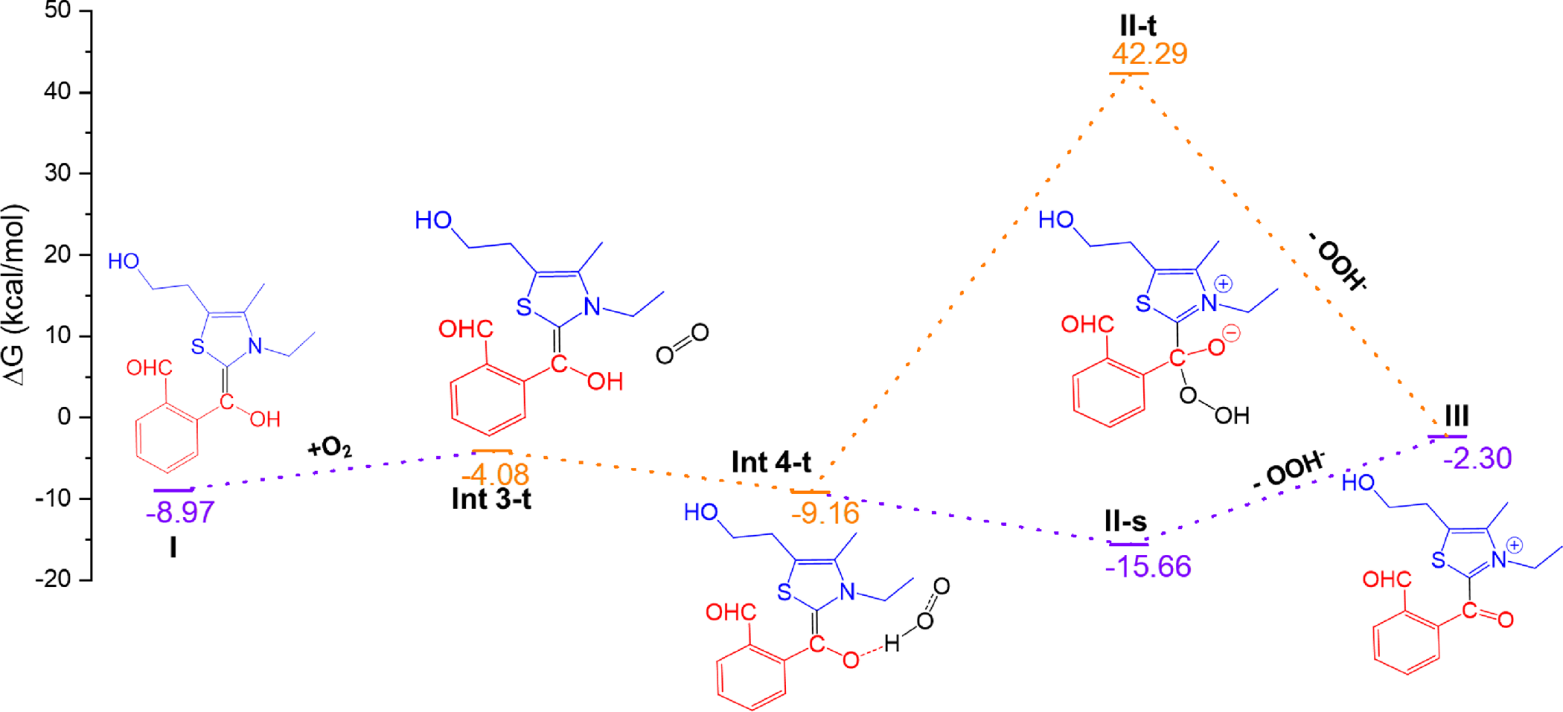
Free energy diagram of the aerial oxidation pathway to generate the acyl azolium intermediate (**III**). Orange color, triplet pathway; violet color, singlet pathway.

The Breslow intermediate (**I**) reacts with molecular oxygen, leading to the triplet intermediate, **Int3-t** (Figure 3). At a distance of 2.790 Å, O_2_ engages in a moderate O–H···O hydrogen bonding with the Breslow intermediate (Figure S33, Supporting Information). Then, the triplet O_2_ takes the proton from the OH group of the Breslow intermediate in **Int4-t** to generate the HOO^–^ ion. A strong H-bonding is formed when the HOO^–^ ion interacts with carbonyl group (Figure S33, Supporting Information). The hydroperoxide intermediates (**II-s** and **II-t**) are formed when the HOO– reacts at the electrophilic carbon center through a barrierless process. Since the **II-s** is energetically more stable than the corresponding triplet state (**II-t**), an intersystem crossing process containing a transition from triplet to singlet occurs. During the reaction, there is an elimination of a HOO^–^ ion from **II** and **III** being formed. However, the dissociation of zwitterionic prerequisites a significant electrostatic effort. Moreover, the HOO^–^ ion is a very strong base and nucleophile. Thus, there is a requirement of receiver species that can capture this leaving anion to assist the process. The second aldehyde or solvent molecule could act as a receiver.^15d^ Finally, the oxidative product (**III**) is formed and is exergonic by 2.30 kcal/mol.

### Intramolecular Annulation

**Figure 4:**
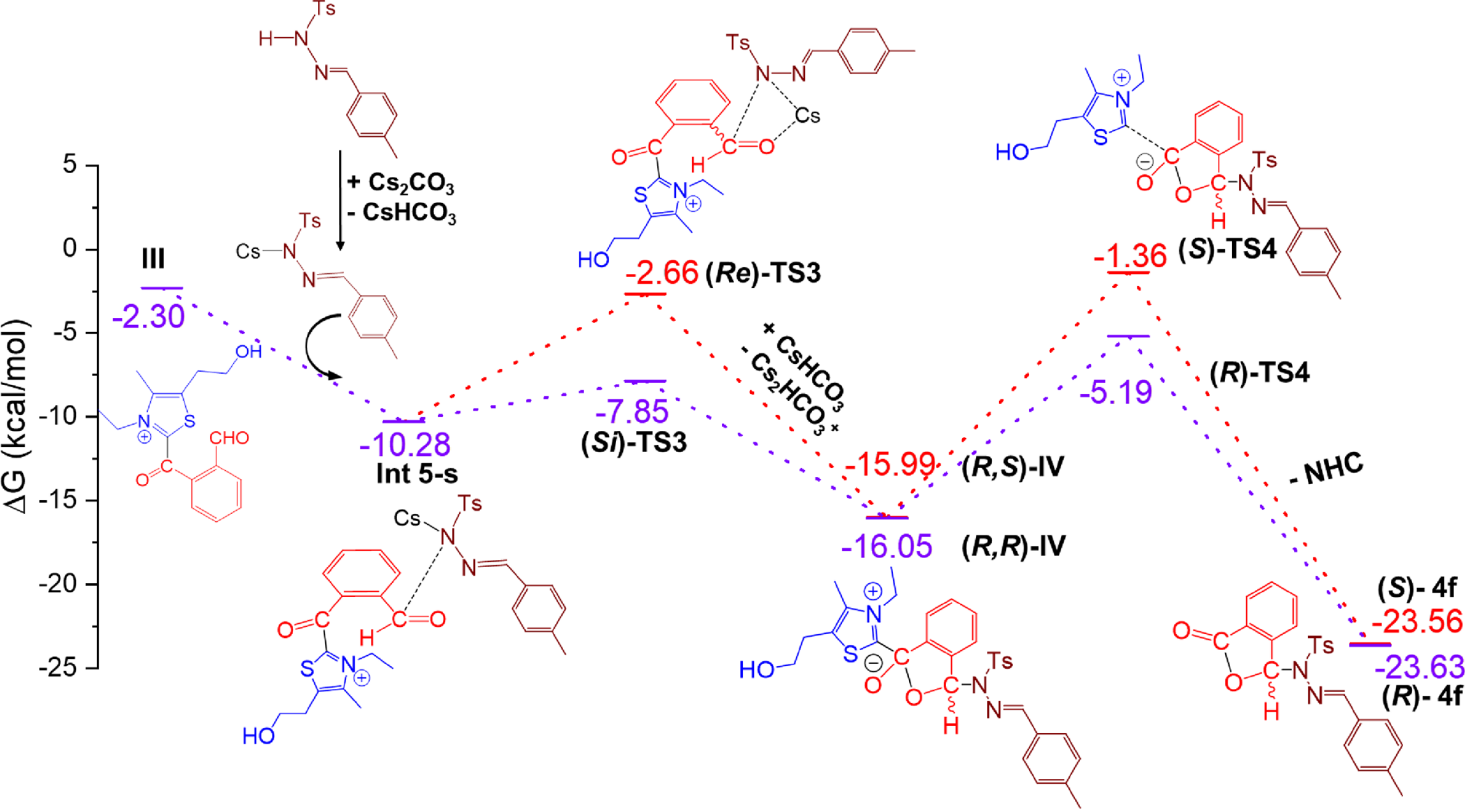
Free energy diagram for forming the final product hemiaminal ester along with the regeneration of the active catalyst. Red color, proceeds through the Re face; violet color, proceeds through the Si face.

As proposed, the nucleophilic addition of *N*-tosyl hydrazone to the oxidized Breslow intermediate (**III**) starts with a vdW complex, **Int 5-s** (Figure 4). Before the nucleophilic addition of *N*-tosyl hydrazine, it should be deprotonated at the N center. In this study, we have considered Cs_2_CO_3_ to remove the hydrogen in *N*-tosyl hydrazone. The production of cesium salt (**Int 5-s**) is energetically favored. Once it is deprotonated, the nucleophilic attack of *N*-tosyl hydrazine occurs at the carbonyl group in **III**. Two stereoisomeric channels associated with the nucleophilic attack of the *re* or *siface* of the carbonyl carbon are feasible. The lengths of the C–N bonds are 1.866 Å (*Re***-TS3**) and 1.981 Å (*Si***-TS3**) (Figure S34, Supporting Information). Thus, the transition state **TS3** is the stereoselectivity determining step responsible for the *R* or *S* configuration of **III**. Thus, the reaction proceeds through O–C coupling to generate intermediate **IV**. Finally, there is extrusion of the NHC catalyst from intermediate **IV** and it produces the final product (**4**). The final step in the reaction follows a pathway through transition state **TS4**, where the activation free energy barrier is 10.86 kcal/mol higher than that of the intermediate **IV**. A free energy difference of 3.83 kcal/mol between **(***S***)-TS4** and **(***R***)-TS4** can be attributed to the fact that **(***R***)-TS4** is sterically less crowded compared to **(***S***)-TS4**. At **(***R***)-TS4**, the distance between NHC carbene C and ester C is measured to be 2.084 Å, whereas at **(***S***)-TS4**, this distance increases to 2.129 Å (Figure S34, Supporting Information). From the separated reactants, the formation of the final product is strongly exergonic by −23.63 kcal/mol.

**Figure 5:**
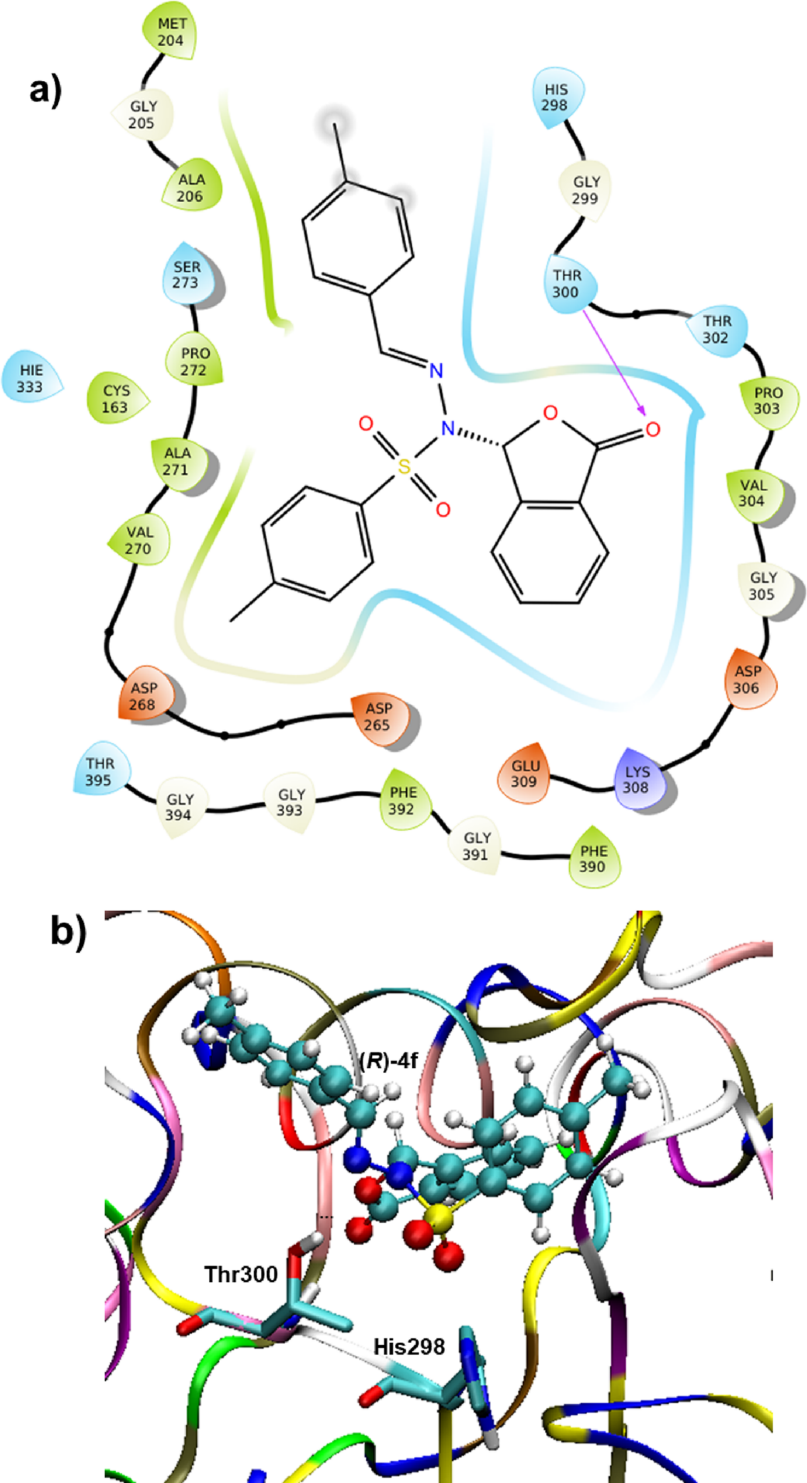
Glide molecular docking interactions of the receptor (PDB ID: 1FJ4) with **(***R***)-4f**. (a) Protein–ligand schematic interaction diagram of the protein and **(***R***)-4f** complex. (b) Binding pose of **(***R***)-4f** in the active site of the receptor.

In this section, we will investigate the potential of one of the phthalidyl sulfonohydrazone derivatives (**4f**) as the inhibitor of β-ketoacyl acyl carrier protein synthase I (KAS I) of *Escherichia coli*. *Escherichia coli*, commonly known as *E. coli*, is a prevalent bacterium responsible for various bacterial infections in humans (Figure 5). These infections encompass a wide range of conditions, including cholecystitis, bacteremia, cholangitis, urinary tract infections, and traveler’s diarrhea, as well as clinical infections like neonatal meningitis and pneumonia. Certainly, three types of β-ketoacyl acyl carrier protein synthase (KAS) enzymes play a crucial role in addressing bacterial resistance issues. Thus, targeting these enzymes can be an effective strategy in tackling antibiotic resistance. Disruption of KAS I can impede the synthesis of essential fatty acids, crucial for bacterial membrane formation and cell growth. Thiolactomycin (TLM), a unique thiolactone molecule comprising natural products, inhibits bacterial cell growth by impeding the β-ketoacyl-ACP synthase activity. Thus, it becomes possible to compare the binding activity of TLM with the final product **4f** (R/S). The docking studies are performed with the protein (1FJ4)^17^ with a resolution factor of 2.35 Å. The active site of KAS I procedures a catalytic triad hole consisting of His–His–Cys.^18,19^ The docking study shows that TLM binds at the active site and forms a hydrogen bond with His333, contributing to the stabilization of the protein–inhibitor complex. The noncovalent interactions of TLM with the amino acids at the active site are elucidated in Figure S35 (Supporting Information). The calculated binding energy of reference TLM is −6.15 kcal/mol, whereas **(***R***)-4** shows a favorable binding energy of −6.87 kcal/mol. **(***S***)-4** has a slightly weak binding interaction (−5.02 kcal/mol) at the active site compared to reference TLM. Thr-300 exhibits strong hydrogen bonding interactions with both compounds (Figure 5 and Figure S36, Supporting Information). Based on the phthalidyl sulfonohydrazone–receptor interactions, it is suggested that these leads have the potential to be developed into effective antimicrobial drugs targeting Gram-negative *E. coli*.

## Conclusions

In conclusion, this study offers an excellent methodology for the synthesis of new phthalidyl sulfonohydrazone compounds using NHC-catalyzed reaction conditions under open air. The reaction mechanism proceeds through the formation of the Breslow intermediate, followed by aerial oxidation. Ultimately, phthalidyl sulfonohydrazones are formed via intramolecular annulation. The full mechanism is supported by both experimental and computational methods. Finally, the phthalidyl sulfonohydrazide-β-ketoacyl acyl carrier protein synthase I interactions are studied successfully using molecular docking, which suggests the potential of phthalidyl sulfonohydrazones to be effective as an antimicrobial drug targeting Gram-negative *E. coli*.

## Experimental Section

All reagents were purchased from commercial suppliers and used without further purification, unless otherwise specified. Commercially supplied ethyl acetate and petroleum ether were distilled before use. All solvents were dried through usual methods. The petroleum ether used in our experiments has a boiling range of 60–80 °C. Analytical thin-layer chromatography was performed on 0.25 mm extra-hard silica gel plates with a UV254 fluorescent indicator. The reported melting points are uncorrected. The ^1^H NMR and ^13^C NMR spectra were recorded at ambient temperature using both 300 MHz spectrometers (300 MHz for ^1^H and 75 MHz for ^13^C). Chemical shifts are reported in ppm with respect to tetramethylsilane internal reference, and coupling constants are reported in Hz. Proton multiplicities are represented as s (singlet), d (doublet), dd (double doublet), t (triplet), q (quartet), and m (multiplet). The infrared spectra were recorded on an FT-IR spectrometer in thin films. HRMS data were recorded on a Q-tof-micro quadruple mass spectrophotometer.

### General Procedure for the Synthesis of Phthalidyl Sulfonohydrazide Derivatives (**4**, GP-1)

In a 25 mL round-bottom flask, phthalaldehyde (**1**, 1.0 mmol) and *N*-tosyl hydrazones^1^ (**2**, 1.0 mmol) were added in CH_3_CN (5 mL) in the presence of thiazolium bromide **3c** (10 mol %) in open-air conditions, and Cs_2_CO_3_ (20 mol %) was added to this reaction mixture and stirred at room temperature for 2–5 h. Upon completion (monitored through TLC), the reaction mixture was filtered through the Celite bed and evaporated in a rotary evaporator under reduced pressure and then extracted with CH_2_Cl_2_ (2 × 15 mL). The combined organic layer was washed with water (3 × 10 mL) and dried over anhydrous Na_2_SO_4_, filtered, and evaporated in a rotary evaporator under reduced pressure at room temperature. The residue was chromatographed on a silica gel column (60–120 mesh) using ethyl acetate–petroleum ether (9 to 20%, v/v) as an eluent, which afforded the corresponding hemiaminal phthalidyl ester derivatives (**4**).

### (*E*)-*N*′-Benzylidene-4-methyl-*N*-(3-oxo-1,3-dihydroisobenzofuran-1-yl)benzenesulfonohydrazide (**4a**)

Compound **4a** was prepared following the GP-1 using phthalaldehyde (**1a**, 1 mmol) and (*E*)-*N*′-benzylidene-4-methylbenzenesulfonohydrazide (**2a**, 1 mmol) as the starting material and afforded the title compound **4a** (using 10% ethyl acetate–petroleum ether (v/v) as an eluent for purification) as white solid, **Yield:** 73% (296.7 mg); **M.P.:** 155–157 °C; ^**1**^**H NMR** (300 MHz, CDCl_3_): δ 8.59 (s, 1H), 7.90–7.85 (m, 3H), 7.70–7.65 (m, 1H), 7.57–7.52 (m, 2H), 7.48 (s, 1H), 7.39–7.24 (m, 7H), 2.45 (s, 3H); ^**13**^**C NMR** (75 MHz, CDCl_3_): δ 168.7, 160.3, 145.3, 144.9, 134.6, 133.8, 133.0, 131.5, 130.4, 129.8, 129.1, 128.6, 128.1, 127.2, 125.5, 123.2, 88.3, 21.7; **FT-IR** (KBr, cm^–1^): 1766, 1596, 1427, 1358, 1044, 956; **HRMS** (ESI-TOF) *m*/*z* Calcd for C_22_H_19_N_2_O_4_S [M + H]^+^: 407.1066, found 407.1070.

### (*E*)-4-Methyl-*N*-(3-oxo-1,3-dihydroisobenzofuran-1-yl)-*N*′-(2,4,6-trimethylbenzylidene)benzenesulfonohydrazide (**4b**)

Compound **4b** was prepared following the GP-1 using phthalaldehyde (**1a**, 1 mmol) and (*E*)-4-methyl-*N*′-(2,4,6-trimethylbenzylidene)benzenesulfonohydrazide (**2b**, 1 mmol) as the starting material and afforded the title compound **4b** (using 9% ethyl acetate–petroleum ether (v/v) as an eluent for purification) as brown solid, **Yield:** 86% (385.7 mg); **M.P.:** 135–137 °C; ^**1**^**H NMR** (300 MHz, CDCl_3_): δ 8.94 (s, 1H), 7.90–7.85 (m, 3H), 7.70–7.66 (m, 1H), 7.58–7.56 (m, 2H), 7.55–7.51 (m, 1H), 7.35 (d, *J* = 8.1 Hz, 2H), 6.74 (m, 2H), 2.45 (s, 3H), 2.22 (s, 3H), 1.94 (s, 6H); ^**13**^**C NMR** (75 MHz, CDCl_3_): δ 168.6, 162.1, 145.2, 145.1, 140.5, 138.8, 134.5, 134.0, 130.4, 129.8, 129.7, 129.1, 127.5, 127.0, 125.5, 123.4, 88.3, 21.7, 21.1, 21.0; **FT-IR** (KBr, cm^–1^): 1772, 1600, 1435, 1400, 1170, 1048, 960; **HRMS** (ESI-TOF) *m*/*z* Calcd for C_25_H_25_N_2_O_4_S [M + H]^+^: 449.1535, found 449.1539.

### (*E*)-*N*′-(2,5-Dimethylbenzylidene)-4-methyl-*N*-(3-oxo-1,3-dihydroisobenzofuran-1-yl)benzenesulfonohydrazide (**4c**)

Compound **4c** was prepared following the GP-1 using phthalaldehyde (**1a**, 1 mmol) and (*E*)-*N*′-(2,5-dimethylbenzylidene)-4-methylbenzenesulfonohydrazide (**2c**, 1 mmol) as the starting material and afforded the title compound **4c** (using 9% ethyl acetate–petroleum ether (v/v) as an eluent for purification) as white solid, **Yield:** 84% (365.0 mg); **M.P.:** 138–140 °C; ^**1**^**H NMR** (300 MHz, CDCl_3_): δ 8.76 (s, 1H), 7.89–7.86 (m, 3H), 7.71–7.66 (m, 1H), 7.57–7.52 (m, 2H), 7.49 (s, 1H), 7.35 (d, *J* = 8.1 Hz, 2H), 7.05–7.03 (m, 2H), 6.97–6.94 (m, 1H), 2.48 (s, 3H), 2.19 (s, 3H), 2.07 (s, 3H); ^**13**^**C NMR** (75 MHz, CDCl_3_): δ 168.7, 160.8, 145.3, 144.9, 135.6, 135.3, 134.5, 133.9, 131.9, 131.0, 130.8, 130.4, 129.8, 129.1, 129.0, 127.3, 125.4, 123.3, 88.3, 21.7, 20.7, 20.7, 19.6; **FT-IR** (KBr, cm^–1^): 1770, 1598, 1440, 1397, 1167, 1049, 962; **HRMS** (ESI-TOF) *m*/*z* Calcd for C_24_H_23_N_2_O_4_S [M + H]^+^: 435.1379, found 435.1381.

### (*E*)-*N*′-(4-Methoxybenzylidene)-4-methyl-*N*-(3-oxo-1,3-dihydroisobenzofuran-1-yl)benzenesulfonohydrazide (**4d**)

Compound **4d** was prepared following the GP-1 using phthalaldehyde (**1a**, 1 mmol) and (*E*)-*N*′-(4-methoxybenzylidene)-4-methylbenzenesulfonohydrazide (**2d**, 1 mmol) as the starting material and afforded the title compound **4d** (using 15% ethyl acetate–petroleum ether (v/v) as an eluent for purification) as colorless liquid, **Yield:** 80% (349.2 mg); ^**1**^**H NMR** (300 MHz, CDCl_3_): δ 8.54 (s, 1H), 7.85–7.80 (m, 3H), 7.67–7.62 (m, 1H), 7.53–7.48 (m, 2H), 7.42 (s, 1H), 7.33 (d, *J* = 8.7 Hz, 4H), 6.87 (d, *J* = 9.0 Hz, 2H), 3.77 (s, 3H), 2.43 (s, 3H); ^**13**^**C NMR** (75 MHz, CDCl_3_): δ 168.7, 162.9, 162.4, 145.0, 144.7, 134.3, 133.8, 130.2, 130.0, 129.5, 129.0, 127.1, 125.4, 125.3, 114.0, 88.2, 55.3, 21.6; **FT-IR** (neat, cm^–1^): 1768, 1609, 1522, 1424, 1167, 1039, 950; **HRMS** (ESI-TOF) *m*/*z* Calcd for C_23_H_21_N_2_O_5_S [M + H]^+^: 437.1171, found 437.1175.

### (*E*)-*N*′-(2-Methoxybenzylidene)-4-methyl-*N*-(3-oxo-1,3-dihydroisobenzofuran-1-yl)benzenesulfonohydrazide (**4e**)

Compound **4e** was prepared following the GP-1 using phthalaldehyde (**1a**, 1 mmol) and (*E*)-*N*′-(2-methoxybenzylidene)-4-methylbenzenesulfonohydrazide (**2e**, 1 mmol) as the starting material and afforded the title compound **4e** (using 14% ethyl acetate–petroleum ether (v/v) as an eluent for purification) as white solid, **Yield:** 78% (340.4 mg); **M.P.:** 125–127 °C; ^**1**^**H NMR** (300 MHz, CDCl_3_): δ 8.96 (s, 1H), 7.88–7.86 (m, 3H), 7.70–7.65 (m, 1H), 7.57–7.54 (m, 2H), 7.48 (s, 1H), 7.36–7.24 (m, 4H), 6.84–6.73 (m, 2H), 3.83 (s, 3H), 2.45 (s, 3H); ^**13**^**C NMR** (75 MHz, CDCl_3_): δ 168.8, 158.7, 156.9, 145.1, 144.9, 134.4, 132.9, 130.3, 129.7, 129.2, 126.7, 125.4, 123.3, 121.5, 120.6, 111.0, 88.3, 55.6, 21.7; **FT-IR** (KBr, cm^–1^): 1764, 1612, 1521, 1420, 1163, 1037, 947; **HRMS** (ESI-TOF) *m*/*z* Calcd for C_23_H_21_N_2_O_5_S [M + H]^+^: 437.1171, found 437.1176.

### (*E*)-4-Methyl-*N*′-(4-methylbenzylidene)-*N*-(3-oxo-1,3-dihydroisobenzofuran-1-yl)benzenesulfonohydrazide (**4f**)

Compound **4f** was prepared following the GP-1 using phthalaldehyde (**1a**, 1 mmol) and (*E*)-4-methyl-*N*′-(4-methylbenzylidene)benzenesulfonohydrazide (**2f**, 1 mmol) as the starting material and afforded the title compound **4f** (using 10% ethyl acetate–petroleum ether (v/v) as an eluent for purification) as white solid, **Yield:** 77% (323.8 mg); **M.P.:** 120–122 °C; ^**1**^**H NMR** (300 MHz, CDCl_3_): δ 8.55 (s, 1H), 7.86–7.80 (m, 3H), 7.67–7.62 (m, 1H), 7.53–7.48 (m, 2H), 7.43 (s, 1H), 7.32 (d, *J* = 8.1 Hz, 2H), 7.26–7.23 (m, 2H), 7.06 (d, *J* = 7.8 Hz, 2H), 2.43 (s, 3H), 2.30 (s, 3H); ^**13**^**C NMR** (75 MHz, CDCl_3_): δ 168.8, 161.9, 145.2, 144.8, 142.3, 134.5, 133.9, 130.33, 130.25, 129.7, 129.4, 128.3, 127.2, 125.4, 123.2, 88.3, 21.8, 21.6; **FT-IR** (KBr, cm^–1^): 1767, 1598, 1432, 1350, 1162, 1069, 940; **HRMS** (ESI-TOF) *m*/*z* Calcd for C_23_H_21_N_2_O_4_S [M + H]^+^: 421.1222, found 421.1220.

### (*E*)-*N*′-(4-Isopropylbenzylidene)-4-methyl-*N*-(3-oxo-1,3-dihydroisobenzofuran-1-yl)benzenesulfonohydrazide (**4g**)

Compound **4g** was prepared following the GP-1 using phthalaldehyde (**1a**, 1 mmol) and (*E*)-*N*′-(4-isopropylbenzylidene)-4-methylbenzenesulfonohydrazide (**2g**, 1 mmol) as the starting material and afforded the title compound **4g** (using 9% ethyl acetate–petroleum ether (v/v) as an eluent for purification) as brown solid, **Yield:** 75% (336.0 mg); **M.P.:** 170–172 °C; ^**1**^**H NMR** (300 MHz, CDCl_3_): δ 8.59 (s, 1H), 7.88–7.84 (m, 3H), 7.69–7.63 (m, 1H), 7.55–7.50 (m, 2H), 7.46 (s, 1H), 7.36–7.28 (m, 4H), 7.14 (d, *J* = 8.1 Hz 2H), 2.92–2.83 (m, 1H), 2.44 (s, 3H), 1.22–1.19 (m, 6H); ^**13**^**C NMR** (75 MHz, CDCl_3_): δ 168.7, 161.6, 153.0, 145.1, 144.8, 134.4, 133.8, 130.6, 130.2, 129.7, 129.0, 128.3, 127.1, 126.7, 125.3, 123.1, 88.2, 34.0, 23.58, 23.56, 21.6; **FT-IR** (KBr, cm^–1^): 1771, 1608, 1440, 1387, 1172, 1041, 942; **HRMS** (ESI-TOF) *m*/*z* Calcd for C_25_H_25_N_2_O_4_S [M + H]^+^: 449.1535, found 449.1540.

### (*E*)-*N*′-(4-(Dimethylamino)benzylidene)-4-methyl-*N*-(3-oxo-1,3-dihydroisobenzofuran-1-yl)benzenesulfonohydrazide (**4h**)

Compound **4h** was prepared following the GP-1 using phthalaldehyde (**1a**, 1 mmol) and (*E*)-*N*′-(4-(dimethylamino)benzylidene)-4-methylbenzenesulfonohydrazide (**2h**, 1 mmol) as the starting material and afforded the title compound **4h** (using 20% ethyl acetate–petroleum ether (v/v) as an eluent for purification) as red solid, **Yield:** 75% (337.1 mg); **M.P.:** 160–163 °C; ^**1**^**H NMR** (300 MHz, CDCl_3_): δ 8.48 (s, 1H), 7.80 (d, *J* = 8.1 Hz, 3H), 7.62–7.59 (m, 1H), 7.52–7.44 (m, 2H), 7.38 (s, 1H), 7.33–7.30 (m, 4H), 6.53 (d, *J* = 8.7 Hz, 2H), 2.96 (s, 6H), 2.44 (s, 3H); ^**13**^**C NMR** (75 MHz, CDCl_3_): δ 168.8, 166.6, 152.6, 144.7, 144.6, 134.19, 134.16, 130.2, 130.1, 129.4, 129.1, 127.1, 125.2, 123.1, 120.0, 111.1, 88.4, 39.9, 21.6; **FT-IR** (KBr, cm^–1^): 1769, 1594, 1444, 1379, 1174, 1042, 945; **HRMS** (ESI-TOF) *m*/*z* Calcd for C_24_H_24_N_3_O_4_S [M + H]^+^: 450.1488, found 450.1485.

### (*E*)-*N*′-(4-Chlorobenzylidene)-4-methyl-*N*-(3-oxo-1,3-dihydroisobenzofuran-1-yl)benzenesulfonohydrazide (**4i**)

Compound **4i** was prepared following the GP-1 using phthalaldehyde (**1a**, 1 mmol) and (*E*)-*N*′-(4-chlorobenzylidene)-4-methylbenzenesulfonohydrazide (**2i**, 1 mmol) as the starting material and afforded the title compound **4i** (using 10% ethyl acetate–petroleum ether (v/v) as an eluent for purification) as white solid, **Yield:** 73% (321.8 mg); **M.P.:** 190–192 °C; ^**1**^**H NMR** (300 MHz, CDCl_3_): δ 8.54 (s, 1H), 7.91–7.84 (m, 3H), 7.71–7.66 (m, 1H), 7.58–7.54 (m, 2H), 7.49 (s, 1H), 7.36 (d, *J* = 8.1 Hz, 2H), 7.24 (m, 4H), 2.45 (s, 3H); ^**13**^**C NMR** (75 MHz, CDCl_3_): δ 168.7, 158.0, 145.5, 144.8, 137.5, 134.6, 133.6, 131.5, 130.4, 129.9, 129.2, 129.0, 128.9, 127.2, 125.5, 123.3, 88.2, 21.8; **FT-IR** (KBr, cm^–1^): 1780, 1595, 1462, 1333, 1168, 1087, 814; **HRMS** (ESI-TOF) *m*/*z* Calcd for C_22_H_18_ClN_2_O_4_S [M + H]^+^: 441.0676, found 441.0680 (one of the major peaks).

### (*E*)-*N*′-(4-Bromobenzylidene)-4-methyl-*N*-(3-oxo-1,3-dihydroisobenzofuran-1-yl)benzenesulfonohydrazide (**4j**)

Compound **4j** was prepared following the GP-1 using phthalaldehyde (**1a**, 1 mmol) and (*E*)-*N*′-(4-bromobenzylidene)-4-methylbenzenesulfonohydrazide (**2j**, 1 mmol) as the starting material and afforded the title compound **4j** (using 10% ethyl acetate–petroleum ether (v/v) as an eluent for purification) as white solid, **Yield:** 70% (339.7 mg); **M.P.:** 196–198 °C; ^**1**^**H NMR** (300 MHz, CDCl_3_): δ 8.53 (s, 1H), 7.91–7.84 (m, 3H), 7.71–7.66 (m, 1H), 7.58–7.49 (m, 2H), 7.49 (s, 1H), 7.41–7.15 (m, 2H), 2.45 (s, 3H); ^**13**^**C NMR** (75 MHz, CDCl_3_): δ 168.7, 157.8, 145.5, 144.8, 134.6, 133.6, 132.0, 130.5, 129.9, 129.3, 129.0, 127.2, 126.0, 125.5, 123.2, 88.2, 21.8; **FT-IR** (KBr, cm^–1^): 1781, 1592, 1471, 1331, 1170, 1070, 812; **HRMS** (ESI-TOF) *m*/*z* Calcd for C_22_H_18_BrN_2_O_4_S [M + H]^+^: 485.0171, found 485.0176 (one of the major peaks).

### (*E*)-*N*′-(3-Fluorobenzylidene)-4-methyl-*N*-(3-oxo-1,3-dihydroisobenzofuran-1-yl)benzenesulfonohydrazide (**4k**)

Compound **4k** was prepared following the GP-1 using phthalaldehyde (**1a**, 1 mmol) and (*E*)-*N*′-(3-fluorobenzylidene)-4-methylbenzenesulfonohydrazide (**2k**, 1 mmol) as the starting material and afforded the title compound **4k** (using 11% ethyl acetate–petroleum ether (v/v) as an eluent for purification) as white solid, **Yield:** 65% (275.9 mg); **M.P.:** 186–188 °C; ^**1**^**H NMR** (300 MHz, CDCl_3_): δ 8.53 (s, 1H), 7.89 (t, *J* = 8.7 Hz, 3H), 7.72–7.67 (m, 1H), 7.59–7.51 (m, 3H), 7.36 (d, *J* = 8.1 Hz, 2H), 7.27–7.20 (m, 1H), 7.09–6.94 (m, 3H), 2.44 (s, 3H); ^**13**^**C NMR** (75 MHz, CDCl_3_): δ 168.6, 164.3 (C–F, ^1^*J*_C–F_ = 245.8 Hz), 161.0 (C–F, ^1^*J*_C–F_ = 245.8 Hz), 156.5 (C–F, ^4^*J*_C–F_ = 2.2 Hz), 156.4 (C–F, ^4^*J*_C–F_ = 2.2 Hz), 145.6, 144.9, 135.3 (C–F, ^3^*J*_C–F_ = 7.7 Hz), 135.2 (C–F, ^3^*J*_C–F_ = 7.7 Hz), 134.6, 133.6, 130.5, 130.3 (C–F, ^3^*J*_C–F_ = 8.0 Hz), 130.2 (C–F, ^3^*J*_C–F_ = 8.0 Hz), 129.9, 129.0, 127.2, 125.5, 124.23 (C–F, ^4^*J*_C–F_ = 2.8 Hz), 124.19 (C–F, ^4^*J*_C–F_ = 2.8 Hz), 123.2, 118.4 (C–F, ^2^*J*_C–F_ = 21.4 Hz), 118.2 (C–F, ^2^*J*_C–F_ = 21.4 Hz), 113.9 (C–F, ^2^*J*_C–F_ = 22.5 Hz), 113.6 (C–F, ^2^*J*_C–F_ = 22.5 Hz), 88.2, 21.7; **FT-IR** (KBr, cm^–1^): 1778, 1602, 1513, 1362, 1322, 1160, 1054, 932; **HRMS** (ESI-TOF) *m*/*z* Calcd for C_22_H_18_FN_2_O_4_S [M + H]^+^: 425.0971, found 425.0975.

### (*E*)-*N*′-(3,4-Dichlorobenzylidene)-4-methyl-*N*-(3-oxo-1,3-dihydroisobenzofuran-1-yl)benzenesulfonohydrazide (**4l**)

Compound **4l** was prepared following the GP-1 using phthalaldehyde (**1a**, 1 mmol) and (*E*)-*N*′-(3,4-dichlorobenzylidene)-4-methylbenzenesulfonohydrazide (**2l**, 1 mmol) as the starting material and afforded the title compound **4l** (using 9% ethyl acetate–petroleum ether (v/v) as an eluent for purification) as yellow solid, **Yield:** 55% (261.4 mg); **M.P.:** 196–198 °C; ^**1**^**H NMR** (300 MHz, CDCl_3_): δ 8.46 (s, 1H), 7.93–7.85 (m, 3H), 7.70 (t, *J* = 7.2 Hz, 1H), 7.61–7.54 (m, 2H), 7.51 (s, 1H), 7.38–7.30 (m, 4H), 7.11–7.08 (m, 1H), 2.45 (s, 3H); ^**13**^**C NMR** (75 MHz, CDCl_3_): δ 168.6, 154.6, 145.7, 144.7, 135.4, 134.7, 133.4, 133.09, 133.07, 130.7, 130.6, 130.0, 129.5, 129.0, 127.1, 126.6, 125.5, 123.2, 88.1, 21.8; **FT-IR** (KBr, cm^–1^): 1779, 1593, 1475, 1337, 1165, 1057, 931; **HRMS** (ESI-TOF) *m*/*z* Calcd for C_22_H_17_Cl_2_N_2_O_4_S [M + H]^+^: 475.0286, found 475.0281 (one of the major peaks).

### (*E*)-4-Methyl-*N*′-(4-nitrobenzylidene)-*N*-(3-oxo-1,3-dihydroisobenzofuran-1-yl)benzenesulfonohydrazide (**4m**)

Compound **4m** was prepared following the GP-1 using phthalaldehyde (**1a**, 1 mmol) and (*E*)-4-methyl-*N*′-(4-nitrobenzylidene)benzenesulfonohydrazide (**2m**, 1 mmol) as the starting material and afforded the title compound **4m** (using 18% ethyl acetate–petroleum ether (v/v) as an eluent for purification) as reddish brown solid, **Yield:** 53% (239.3 mg); **M.P.:** 160–162 °C; ^**1**^**H NMR** (300 MHz, CDCl_3_): δ 8.55 (s, 1H), 8.10–8.07 (m, 2H), 7.96–7.89 (m, 3H), 7.72 (t, *J* = 7.5 Hz, 1H), 7.64–7.57 (m, 3H), 7.40–7.28 (m, 4H), 2.45 (s, 3H); ^**13**^**C NMR** (75 MHz, CDCl_3_): δ 168.4, 151.2, 149.0, 145.9, 144.7, 139.0, 134.7, 133.3, 130.6, 130.1, 128.9, 128.1, 127.2, 125.6, 123.8, 123.2, 88.1, 21.7; **FT-IR** (KBr, cm^–1^): 1767, 1597, 1480, 1340, 1170, 1060, 935; **HRMS** (ESI-TOF) *m*/*z* Calcd for C_22_H_18_N_3_O_6_S [M + H]^+^: 452.0916, found 452.0921.

### (*E*)-4-Methyl-*N*′-(naphthalen-1-ylmethylene)-*N*-(3-oxo-1,3-dihydroisobenzofuran-1-yl)benzenesulfonohydrazide (**4n**)

Compound **4n** was prepared following the GP-1 using phthalaldehyde (**1a**, 1 mmol) and (*E*)-4-methyl-*N*′-(naphthalen-1-ylmethylene)benzenesulfonohydrazide (**2n**, 1 mmol) as the starting material and afforded the title compound **4n** (using 12% ethyl acetate–petroleum ether (v/v) as an eluent for purification) as brown solid, **Yield:** 65% (296.7 mg); **M.P.:** 154–156 °C; ^**1**^**H NMR** (300 MHz, CDCl_3_): δ 9.21 (s, 1H), 8.04 (d, *J* = 8.4 Hz, 1H), 7.95–7.91 (m, 3H), 7.86–7.78 (m, 2H), 7.69- 7.66 (m, 1H), 7.63–7.60 (m, 1H), 7.57–7.55 (m, 2H), 7.48–7.43 (m, 2H), 7.40–7.32 (m, 4H), 2.41 (s, 3H); ^**13**^**C NMR** (75 MHz, CDCl_3_): δ 168.7, 161.0, 145.4, 145.0, 134.6, 133.8, 133.6, 132.2, 130.5, 130.4, 130.0, 129.9, 129.2, 128.6, 128.5, 127.5, 127.4, 126.3, 125.5, 125.0, 124.3, 123.4, 88.5, 21.7; **FT-IR** (KBr, cm^–1^): 1765, 1590, 1509, 1320, 1172, 1065, 944; **HRMS** (ESI-TOF) *m*/*z* Calcd for C_26_H_21_N_2_O_4_S [M + H]^+^: 457.1222, found 457.1221.

### (*E*)-*N*′-([1,1′-Biphenyl]-2-ylmethylene)-4-methyl-*N*-(3-oxo-1,3-dihydroisobenzofuran-1-yl)benzenesulfonohydrazide (**4o**)

Compound **4o** was prepared following the GP-1 using phthalaldehyde (**1a**, 1 mmol) and (*E*)-*N*′-([1,1′-biphenyl]-2-ylmethylene)-4-methylbenzenesulfonohydrazide (**2o**, 1 mmol) as the starting material and afforded the title compound **4o** (using 10–20% ethyl acetate–petroleum ether (v/v) as an eluent for purification) as orange solid, **Yield:** 63% (304.0 mg); **M.P.:** 170–172 °C; ^**1**^**H NMR** (300 MHz, CDCl_3_): δ 8.45 (s, 1H), 7.87 (d, *J* = 7.5 Hz, 1H), 7.74–7.69 (m, 3H), 7.56–7.49 (m, 6H), 7.45–7.39 (m, 2H), 7.32–7.25 m, 3H), 7.19–7.15 (m, 3H), 2.44 (s, 3H); ^**13**^**C NMR** (75 MHz, CDCl_3_): δ 168.6, 159.1, 145.2, 144.9, 143.6, 138.8, 134.5, 134.0, 131.0, 130.5, 130.4, 130.2, 129.8, 129.0, 128.5, 127.9, 127.6, 126.3, 125.6, 123.2, 88.1, 21.8; **FT-IR** (KBr, cm^–1^): 1770, 1594, 1490, 1318, 1175, 1062, 947; **HRMS** (ESI-TOF) *m*/*z* Calcd for C_28_H_23_N_2_O_4_S [M + H]^+^: 483.1379, found 483.1377.

### (*E*)-4-Methyl-*N*-(3-oxo-1,3-dihydroisobenzofuran-1-yl)-*N*′-(pyren-2-ylmethylene)benzenesulfonohydrazide (**4p**)

Compound **4p** was prepared following the GP-1 using phthalaldehyde (**1a**, 1 mmol) and (*E*)-4-methyl-*N*′-(pyren-1-ylmethylene)benzenesulfonohydrazide (**2p**, 1 mmol) as the starting material and afforded the title compound **4p** (using 13% ethyl acetate–petroleum ether (v/v) as an eluent for purification) as brown oily liquid, **Yield:** 60% (318.3 mg); ^**1**^**H NMR** (300 MHz, CDCl_3_): δ 9.55 (s, 1H), 8.25–8.21 (m, 2H), 8.13–8.10 (m, 2H), 7.98 (t, *J* = 4.2 Hz, 6H), 7.91–7.84 (m, 3H), 7.67–7.63 (m, 2H), 7.59 (s, 1H), 7.56–7.51 (m, 1H), 7.33 (d, *J* = 8.1 Hz, 2H), 2.38 (s, 3H); ^**13**^**C NMR** (75 MHz, CDCl_3_): δ 168.8, 160.3, 145.4, 145.0, 134.6, 133.9, 130.9, 130.5, 129.9, 129.8, 129.2, 129.1, 127.4, 127.1, 126.3, 126.2, 126.0, 125.5, 125.1, 124.6, 123.4, 122.3, 88.6, 21.7; **FT-IR** (neat, cm^–1^): 1771, 1593, 1489, 1317, 1171, 1063, 948; **HRMS** (ESI-TOF) *m*/*z* Calcd for C_32_H_23_N_2_O_4_S [M + H]^+^: 531.1379, found 531.1384.

### (*E*)-4-Methyl-*N*-(3-oxo-1,3-dihydroisobenzofuran-1-yl)-*N*′-(thiophen-2-ylmethylene)benzenesulfonohydrazide (**4q**)

Compound **4q** was prepared following the GP-1 using phthalaldehyde (**1a**, 1 mmol) and (*E*)-4-methyl-*N*′-(thiophen-2-ylmethylene)benzenesulfonohydrazide (**2q**, 1 mmol) as the starting material and afforded the title compound **4q** (using 15% ethyl acetate–petroleum ether (v/v) as an eluent for purification) as white solid, **Yield:** 75% (309.4 mg); **M.P.:** 164–166 °C; ^**1**^**H NMR** (300 MHz, CDCl_3_): δ 8.71 (s, 1H), 7.86–7.81 (m, 3H), 7.68–7.63 (m, 1H), 7.54–7.50 (m, 2H), 7.40 (s, 1H), 7.35–7.25 (m, 4H), 6.97 (t, *J* = 4.2 Hz, 1H), 2.42 (s, 3H); ^**13**^**C NMR** (75 MHz, CDCl_3_): δ 168.6, 155.9, 145.3, 144.7, 137.7, 134.5, 133.8, 132.7, 130.8, 130.4, 129.8, 129.1, 127.6, 127.2, 125.5, 123.2, 88.2, 21.8; **FT-IR** (KBr, cm^–1^): 1791, 1588, 1432, 1342, 1167, 1062, 946; **HRMS** (ESI-TOF) *m*/*z* Calcd for C_20_H_17_N_2_O_4_S_2_ [M + H]^+^: 413.0630, found 413.0633.

### (*E*)-4-Methyl-*N*-(3-oxo-1,3-dihydroisobenzofuran-1-yl)-*N*′-(pyridin-3-ylmethylene)benzenesulfonohydrazide (**4r**)

Compound **4r** was prepared following the GP-1 using phthalaldehyde (**1a**, 1 mmol) and (*E*)-4-methyl-*N*′-(pyridin-3-ylmethylene)benzenesulfonohydrazide (**2r**, 1 mmol) as the starting material and afforded the title compound **4r** (using 17% ethyl acetate–petroleum ether (v/v) as an eluent for purification) as white solid, **Yield:** 70% (285.2 mg); **M.P.:** 170–172 °C; ^**1**^**H NMR** (300 MHz, CDCl_3_): δ 8.56–8.47 (m, 3H), 7.92–7.87 (m, 3H), 7.73–7.67 (m, 1H), 7.60–7.50 (m, 4H), 7.36 (d, *J* = 8.1 Hz, 2H), 7.18–7.14 (m, 1H), 2.44 (s, 3H); ^**13**^**C NMR** (75 MHz, CDCl_3_): δ 168.6, 153.5, 151.8, 149.6, 145.7, 144.8, 134.9, 134.7, 133.9, 133.4, 130.5, 130.0, 129.2, 129.0, 127.2, 125.5, 124.0, 123.7, 123.2, 88.1, 21.7; **FT-IR** (KBr, cm^–1^): 1783, 1607, 1436, 1355, 1168, 1048, 942; **HRMS** (ESI-TOF) *m*/*z* Calcd for C_21_H_18_N_3_O_4_S [M + H]^+^: 408.1018, found 408.1015.

### 4-Methyl-*N*-(3-oxo-1,3-dihydroisobenzofuran-1-yl)-*N*′-((1*E*,2*E*)-3-phenylallylidene)benzenesulfonohydrazide (**4s**)

Compound **4s** was prepared following the GP-1 using phthalaldehyde (**1a**, 1 mmol) and 4-methyl-*N*′-((1*E*,2*E*)-3-phenylallylidene)benzenesulfonohydrazide (**2s**, 1 mmol) as the starting material and afforded the title compound **4s** (using 13% ethyl acetate–petroleum ether (v/v) as an eluent for purification) as orange solid, **Yield:** 68% (294.1 mg); **M.P.:** 160–162 °C; ^**1**^**H NMR** (300 MHz, CDCl_3_): δ 8.42 (d, *J* = 9.3 Hz, 1H), 7.88–7.85 (m, 3H), 7.69 (t, *J* = 7.2 Hz, 1H), 7.58–7.52 (m, 2H), 7.43 (s, 1H), 7.39–7.31 (m, 7H), 6.95 (d, *J* = 16.2 Hz, 1H), 6.57–6.49 (m, 1H), 2.45 (s, 3H); ^**13**^**C NMR** (75 MHz, CDCl_3_): δ 168.5, 164.5, 145.1, 144.6, 144.2, 134.9, 134.4, 133.7, 130.3, 129.8, 129.7, 129.0, 128.7, 127.4, 127.2, 125.4, 125.4, 124.1, 123.1, 88.0, 21.6; **FT-IR** (KBr, cm^–1^): 1767, 1650, 1600, 1435, 1352, 1171, 1069, 923; **HRMS** (ESI-TOF) *m*/*z* Calcd for C_24_H_21_N_2_O_4_S [M + H]^+^: 433.1222, found 433.1224.

### (*E*)-4-Methyl-*N*-(3-oxo-1,3-dihydroisobenzofuran-1-yl)-*N*′-(1-phenylethylidene)benzenesulfonohydrazide (**4t**)

Compound **4t** was prepared following the GP-1 using phthalaldehyde (**1a**, 1 mmol) and (*E*)-4-methyl-*N*′-(1-phenylethylidene)benzenesulfonohydrazide (**2t**, 1 mmol) as the starting material and afforded the title compound **4t** (using 11% ethyl acetate–petroleum ether (v/v) as an eluent for purification) as white solid, **Yield:** 65% (273.3 mg); **M.P.:** 164–166 °C; ^**1**^**H NMR** (300 MHz, CDCl_3_): δ 7.81–7.76 (m, 3H), 7.61 (t, *J* = 7.5 Hz, 1H), 7.52–7.46 (m, 4H), 7.41–7.29 (m, 6H), 2.55 (s, 3H), 2.49 (s, 3H); ^**13**^**C NMR** (75 MHz, CDCl_3_): δ 180.5, 168.5, 144.8, 144.2, 136.2, 134.2, 134.1, 131.4, 130.5, 129.4, 129.3, 128.4, 127.2, 127.1, 125.6, 122.7, 88.7, 21.8, 18.4; **FT-IR** (KBr, cm^–1^): 1782, 1603, 1470, 1365, 1170, 1049, 942; **HRMS** (ESI-TOF) *m*/*z* Calcd for C_23_H_21_N_2_O_4_S [M + H]^+^: 421.1222, found 421.1219.

### (*E*)-4-Methyl-*N*-(3-oxo-1,3-dihydroisobenzofuran-1-yl)-*N*′-(1-(*p*-tolyl)ethylidene)benzenesulfonohydrazide (**4u**)

Compound **4u** was prepared following the GP-1 using phthalaldehyde (**1a**, 1 mmol) and (*E*)-4-methyl-*N*′-(1-(*p*-tolyl)ethylidene)benzenesulfonohydrazide (**2u**, 1 mmol) as the starting material and afforded the title compound **4u** (using 10% ethyl acetate–petroleum ether (v/v) as an eluent for purification) as colorless oily liquid, **Yield:** 70% (304.1 mg); ^**1**^**H NMR** (300 MHz, CDCl_3_): δ 7.81–7.75 (m, 3H), 7.62–7.57 (m, 1H), 7.48–7.41 (m, 4H), 7.36–7.28 (m, 3H), 7.09 (d, *J* = 8.1 Hz, 2H), 2.52–2.49 (m, 6H), 2.34 (s, 3H); ^**13**^**C NMR** (75 MHz, CDCl_3_): δ 180.1, 168.5, 144.8, 144.2, 142.0, 134.2, 134.0, 133.5, 130.5, 129.4, 129.3, 129.0, 127.3, 127.1, 125.5, 122.7, 88.8, 21.8, 21.4, 18.2; **FT-IR** (neat, cm^–1^): 1772, 1605, 1436, 1355, 1164, 1073, 942; **HRMS** (ESI-TOF) *m*/*z* Calcd for C_24_H_23_N_2_O_4_S [M + H]^+^: 435.1379, found 435.1382.

### (*E*)-*N*′-(1-(4-Bromophenyl)ethylidene)-4-methyl-*N*-(3-oxo-1,3-dihydroisobenzofuran-1-yl)benzenesulfonohydrazide (**4v**)

Compound **4v** was prepared following the GP-1 using phthalaldehyde (**1a**, 1 mmol) and (*E*)-*N*′-(1-(4-bromophenyl)ethylidene)-4-methylbenzenesulfonohydrazide (**2v**, 1 mmol) as the starting material and afforded the title compound **4v** (using 9% ethyl acetate–petroleum ether (v/v) as an eluent for purification) as white solid, **Yield:** 60% (299.6 mg); **M.P.:** 170–172 °C; ^**1**^**H NMR** (300 MHz, CDCl_3_): δ 7.77 (d, *J* = 7.8 Hz, 3H), 7.61 (d, *J* = 7.8 Hz, 1H), 7.50–7.33 (m, 9H), 2.52–2.49 (m, 6H); ^**13**^**C NMR** (75 MHz, CDCl_3_): δ 179.5, 169.2, 145.0, 144.1, 134.9, 134.1, 131.6, 130.6, 129.5, 129.3, 128.7, 126.8, 126.4, 125.6, 124.0, 122.6, 88.7, 21.8, 18.2; **FT-IR** (KBr, cm^–1^): 1785, 1593, 1433, 1345, 1168, 1072, 949; **HRMS** (ESI-TOF) *m*/*z* Calcd for C_23_H_20_BrN_2_O_4_S [M + H]^+^: 499.0327, found 499.0329 (one of the major peaks).

### (*E*)-4-Methyl-*N*-(3-oxo-1,3-dihydroisobenzofuran-1-yl)-*N*′-(1-(thiophen-2-yl)ethylidene)benzenesulfonohydrazide (**4w**)

Compound **4w** was prepared following the GP-1 using phthalaldehyde (**1a**, 1 mmol) and (*E*)-4-methyl-*N*′-(1-(thiophen-2-yl)ethylidene)benzenesulfonohydrazide (**2w**, 1 mmol) as the starting material and afforded the title compound **4w** (using 15% ethyl acetate–petroleum ether (v/v) as an eluent for purification) as white solid, **Yield**: 64% (273.0 mg); **M.P**.: 160–164 °C; ^**1**^**H NMR** (300 MHz, CDCl_3_): δ 7.80–7.75 (m, 3H), 7.64–7.59 (m, 2H), 7.49–7.44 (m, 2H), 7.37–7.28 (m, 3H), 7.22–7.16 (m, 2H), 2.51–2.50 (m, 6H); ^**13**^**C NMR** (75 MHz, CDCl_3_): δ 175.6, 168.5, 144.8, 144.2, 139.5, 134.1, 134.0, 130.5, 129.4, 129.3, 128.6, 127.1, 126.1, 126.0, 125.5, 122.7, 88.7, 21.8, 18.5; **FT-IR** (KBr, cm^–1^): 1792, 1590, 1434, 1340, 1166, 1067, 945; **HRMS** (ESI-TOF) *m*/*z* Calcd for C_21_H_19_N_2_O_4_S_2_ [M + H]^+^: 427.0786, found 427.0781.

### (*E*)-*N*-(5-Methoxy-3-oxo-1,3-dihydroisobenzofuran-1-yl)-4-methyl-*N*′-(4-methylbenzylidene)benzenesulfonohydrazide (**4x**)

Compound **4x** was prepared following the GP-1 using 4-methoxyphthalaldehyde (**1b**, 1 mmol) and (*E*)-4-methyl-*N*′-(4-methylbenzylidene)benzenesulfonohydrazide (**2f**, 1 mmol) as the starting material and afforded the title compound **4x** (using 15% ethyl acetate–petroleum ether (v/v) as an eluent for purification) as white solid, **Yield:** 60% (270.3 mg); **M.P.:** 148–150 °C; ^**1**^**H NMR** (300 MHz, CDCl_3_): δ 8.56 (s, 1H), 7.82 (d, *J* = 8.4 Hz, 2H), 7.42–7.28 (m, 7H), 7.21–7.17 (m, 1H), 7.11 (d, *J* = 7.8 Hz, 2H), 3.84 (s, 3H), 2.45 (s, 3H), 2.34 (s, 3H); ^**13**^**C NMR** (75 MHz, CDCl_3_): δ 168.8, 162.6, 161.5, 145.1, 142.3, 136.9, 134.0, 130.3, 129.7, 129.4, 129.1, 128.8, 128.4, 124.1, 123.0, 107.6, 88.2, 55.8, 21.7, 21.6; **FT-IR** (KBr, cm^–1^): 1765, 1597, 1430, 1355, 1165, 1068, 937; **HRMS** (ESI-TOF) *m*/*z* Calcd for C_24_H_23_N_2_O_5_S [M + H]^+^: 451.1328, found 451.1331.

### Computational Methods

All the geometries considered in this study have been fully optimized using the dispersion-corrected PBE0-D3^20,21^ functional with the def2-TZVPP basis set.^22^ The solvent effects were considered via the COSMO solvation model^23^ with acetonitrile solvent medium. The vibrational frequencies of each stationary point were carried out at the same level of theory to classify the stationary points either as real minima (with no imaginary frequencies) or as transition state with only one imaginary frequency. All the calculations were performed using Gaussian 16.^24^

Molecular docking was performed using the Schrodinger Suite molecular modeling package (version 2021-3) using the default parameters. Co-crystal structures of thiolactomycin with β-ketoacyl-[acyl carrier protein] 2 synthase (PDB: 1FJ4),^17^ with a resolution of 2.35 Å, were used as templates and were prepared using the Protein Preparation Wizard. In this step, force field atom types and bond orders were assigned, missing atoms were added, tautomer/ionization states were assigned, and the tautomers of ionizable residues (Asn, Gln, and His residues) were adjusted to optimize the hydrogen bond network. Hydrogen-constrained energy minimization was then performed. Glide SP docking was used to grant full flexibility of ligands into the active site.^25,26^ A postdocking minimization, in which only the ligands were flexible, was performed on the output complexes. The binding energies were calculated for each ligand.

## Data Availability

The data underlying this study are available in the published article and its Supporting Information.

## Supporting Information Available

ESI-MS spectra; spectroscopic and XRD data; NMR spectra; computational details; and additional figures (PDF)

## Accession Codes

CCDC 2080548 contains the supplementary crystallographic data for this paper. The data can be obtained free of charge via www.ccdc.cam.ac.uk/data_request/cif, or by emailing data_request@ccdc.cam.ac.uk, or by contacting The Cambridge Crystallographic Data Centre, 12 Union Road, Cambridge CB2 1EZ, UK; fax: +44 1223 336033.

## References

[ref1] aLesch J. E. The First Miracle Drugs: How the Sulfa Drugs Transformed Medicine; Oxford University Press, 2007.

[ref2] aLoncle C.; Brunel J.; Vidal N.; Dherbomez M.; Letourneux Y. Synthesis and antifungal activity of cholesterol-hydrazone derivatives. Eur. J. Med. Chem. 2004, 39, 1067–1071. 10.1016/j.ejmech.2004.07.005. 15571868

[ref3] aMelnyk P.; Leroux V.; Sergheraert C.; Grellier P. Design, synthesis and in vitro antimalarial activity of an acylhydrazone library. Bioorg. Med. Chem. Lett. 2006, 16, 31–35. 10.1016/j.bmcl.2005.09.058. 16263280

[ref4] Abdel-Aal M. T.; El-Sayed W. A.; El-Ashry E. H. Synthesis and Antiviral Evaluation of Some Sugar Arylglycinoylhydrazones and Their Oxadiazoline Derivatives. Arch. Pharm. Chem. Life Sci. 2006, 339, 656–663. 10.1002/ardp.200600100. 17149795

[ref5] aSilva A. G.; Zapata-Suto G.; Kummerle A. E.; Fraga C. A. M.; Barreiro E. J.; Sudo R. T. Synthesis and vasodilatory activity of new *N*-acylhydrazone derivatives, designed as LASSBio-294 analogues. Bioorg. Med. Chem. 2005, 13, 3431–3437. 10.1016/j.bmc.2005.03.003. 15848755

[ref6] aPatole J.; Sandbhor U.; Padhye S.; Deobagkar D. N.; Anson C. E.; Powell A. Structural chemistry and In vitro antitubercular activity of acetylpyridine benzoyl hydrazone and its copper complex against *Mycobacterium smegmatis*. Bioorg. Med. Chem. Lett. 2003, 13, 51–55. 10.1016/S0960-894X(02)00855-7. 12467615

[ref7] aXie Z.; Song Y.; Xu L.; Guo Y.; Zhang M.; Li L.; Chen K.; Liu X. Rapid Synthesis of N-Tosylhydrazones under Solvent-Free Conditions and Their Potential Application Against HumanTriple-Negative Breast Cancer. ChemistryOpen 2018, 7, 977–983. 10.1002/open.201800206. 30524923 PMC6276103

[ref8] aBarluenga J.; Valdés C. Tosylhydrazones: New Uses for Classic Reagents in Palladium-Catalyzed Cross-Coupling and Metal-Free Reactions. Angew. Chem., Int. Ed. 2011, 50, 7486–7500. 10.1002/anie.201007961. 21748827

[ref9] aLiu L.; Zhang J. L. Gold-catalyzed transformations of a-diazocarbonyl compounds: selectivity and diversity. Chem. Soc. Rev. 2016, 45, 506–516. 10.1039/C5CS00821B. 26658761

[ref10] aPiotrowski D. W.; Kamlet A. S.; Dechert-Schmitt A. -M. R.; Yan J.; Brandt T. A.; Xiao J.; Wei L.; Barrila M. T. Regio- and Enantioselective Synthesis of Azole Hemiaminal Esters by Lewis-Base-Catalyzed Dynamic Kinetic Resolution. J. Am. Chem. Soc. 2016, 138, 4818–4823. 10.1021/jacs.6b00207. 27003237

[ref11] aDineva S.; Uzunova K.; Pavlova V.; Filipova E.; Kalinov K.; Vekov T. Comparative efficacy and safety of chlorthalidone and hydrochlorothiazide—meta-analysis. J. Hum. Hypertens. 2019, 33, 766–774. 10.1038/s41371-019-0255-2. 31595024 PMC6892412

[ref12] aWormser G. P.; Keusch G. T.; Heel R. C. Co-trimoxazole (Trimethoprim-sulfamethoxazole) An Updated Review of its Antibacterial Activity and Clinical Efficacy. Drugs 1982, 24, 459–518. 10.2165/00003495-198224060-00002. 6759092

[ref13] aBiju A. T. “N-Heterocyclic Carbenes in Organocatalysis” 2019 Wiley-VCH Verlag GmbH & Co. KGaA.

[ref14] aLiu Y.; Chen Q.; Mou C.; Pan L.; Duan X.; Chen X.; Chen H.; Zhao Y.; Lu Y.; Jin Z.; Chi Y. R. Catalytic asymmetric acetalization of carboxylic acids for access to chiral phthalidyl ester prodrugs. Nat. Commun. 2019, 10, 1675 10.1038/s41467-019-09445-x. 30975988 PMC6459872

[ref15] aKiran I. N. C.; Lalwani K.; Sudalai A. *N*-Heterocyclic carbene catalyzed esterification of aromatic aldehydes with alcohols under aerobic conditions. RSC Adv. 2013, 3, 1695–1698. 10.1039/C2RA22718E.

[ref16] aShyam A.; Pradhan A. K.; Mondl P. Mechanistic insights into the N-Heterocyclic Carbene catalyzed synthesis α,δ-diketones: a dft approach. ChemistrySelect 2020, 5, 11996–12008. 10.1002/slct.202002811.

[ref17] Price A. C.; Choi K.-H.; Heath R. J.; Li Z.; White S. W.; Rock C. O. Inhibition of beta-ketoacyl-acyl carrier protein synthases by thiolactomycin and cerulenin. Structure and mechanism. J. Biol. Chem. 2001, 276, 6551–6559. 10.1074/jbc.M007101200. 11050088

[ref18] Magnuson K.; Jackowski S.; Rock C. O.; Cronan J. E. Jr. Regulation of fatty acid biosynthesis in Escherichia coli. Microbiol. Rev. 1993, 57, 522–542. 10.1128/mr.57.3.522-542.1993. 8246839 PMC372925

[ref19] De Mendoza D.; Cronan J. E. Jr Thermal regulation of membrane lipid fluidity in bacteria. Trends Biochem. Sci. 1983, 8, 49–52. 10.1016/0968-0004(83)90388-2.

[ref20] Adamo C.; Barone V. Toward reliable density functional methods without adjustable parameters: The PBE0 model. J. Chem. Phys. 1999, 110, 6158–6170. 10.1063/1.478522.

[ref21] Grimme S.; Antony J.; Ehrlich S.; Krieg H. A consistent and accurate ab initio parametrization of density functional dispersion correction (DFT-D) for the 94 elements H-Pu. J. Chem. Phys. 2010, 132, 154104 10.1063/1.3382344. 20423165

[ref22] Weigend F.; Ahlrichs R. Balanced basis sets of split valence, triple zeta valence and quadruple zeta valence quality for H to Rn: Design and assessment of accuracy. Phys. Chem. Chem. Phys. 2005, 7, 3297–3305. 10.1039/b508541a. 16240044

[ref23] Klamt A.; Schüürmann G. COSMO: a new approach to dielectric screening in solvents with explicit expressions for the screening energy and its gradient. J. Chem. Soc., Perkin Trans. 1993, 2 (2), 799–805. 10.1039/P29930000799.

[ref24] Gaussian 16, Revision A.03, Frisch M. J., Gaussian, Inc.: Wallingford CT, 2016.

[ref25] Halgren T. A.; Murphy R. B.; Friesner R. A.; Beard H. S.; Frye L. L.; Pollard W. T.; Banks J. L. Glide: A New Approach for Rapid, Accurate Docking and Scoring. 2. Enrichment Factors in Database Screening. J. Med. Chem. 2004, 47, 1750–1759. 10.1021/jm030644s. 15027866

[ref26] Friesner R. A.; Banks J. L.; Murphy R. B.; Halgren T. A.; Klicic J. J.; Mainz D. T.; Repasky M. P.; Knoll E. H.; Shelley M.; Perry J. K.; Shaw D. E.; Francis P.; Shenkin P. S. Glide: A New Approach for Rapid, Accurate Docking and Scoring. 1. Method and Assessment of Docking Accuracy. J. Med. Chem. 2004, 47, 1739–1749. 10.1021/jm0306430. 15027865

