# Supplementary material for: N-Heterocyclic Carbene-Catalyzed Facile Synthesis of Phthalidyl Sulfonohydrazones: Density Functional Theory Mechanistic Insights and Docking Interactions
Source: ACS Omega. 2024 Feb 26;9(10):11510–22. doi: 10.1021/acsomega.3c08529 (PMC10938401; doi:10.1021/acsomega.3c08529)
Supplement: Supplementary file 1 — ao3c08529_si_001.pdf [file ao3c08529_si_001.pdf]

Supporting Information

N-Heterocyclic Carbene-Catalyzed Facile Synthesis of Phthalidyl Sulfonohydrazones: Density Functional Theory Mechanistic Insights and Docking Interactions

Tanmoy Ghosh,[a‖] Debabrata Barman,[a‖] Krishanu Show,[b] Rabindranath Lo,*,[c] Debashree Manna,*,[c,d] Tapas Ghosh*,[e] and Dilip K. Maiti*,[a]

[a]Dr. Debabrata Barman, Dr. Tanmoy Ghosh, Dr. Dilip K. Maiti
Department of Chemistry, University of Calcutta, Kolkata 700009, India.
Email:dkmchem@caluniv.ac.in
[b]Dr. Krishanu Show, Department of Chemistry, Malda College, Malda 732101, West Bengal, India
[c]Dr. Rabindranath Lo, Dr. Debashree Manna
Institute of Organic Chemistry and Biochemistry, Czech Academy of Sciences, v.v.i., Flemingovo nám. 2, 16610 Praha 6, Czech Republic, Email: rabindranath.lo@uochb.cas.cz, debashree.manna@uochb.cas.cz
[d]Dr. Debashree Manna, Department of Applied Chemistry, Maulana Abul Kalam Azad University of Technology, West Bengal, Simhat, Haringhata-741249, W.B., India. Email: debashree.manna@uochb.cas.cz
[e]Dr. Tapas Ghosh, Department of Chemistry, Jadavpur University, Kolkata 700032, India. Email: tapasg.chemistry@jadavpuruniversity.in
[‖]Equal contribution

Serial No.	Content	Page Numbers
1.	Plausible mechanism of N-Heterocyclic Carbene-Catalyzed oxidative cyclization reaction and ESI-MS study of intermediates	S-02
2.	^{1}H and ^{13}C-NMR spectra of phthalidyl sulfonohydrazones (**4a-x**)	S-04
3.	X-ray crystallographic characterization data of compound **4m**	S-29
4.	Computational analysis of reaction pathway and study of bioactivity	S-31

1. **Plausible mechanism of N-Heterocyclic Carbene-Catalyzed oxidative cyclization reaction and ESI-MS study of intermediates**

The exact mechanism of the new organocatalysis is not realized convincingly. We have envisioned that at first Breslow intermediate **I** is formed *via* nucleophilic addition of the NHC to phthalaldehyde (**1a**).In an open air oxidation by O_2 give Intermediate **II** which then convert into the acyl azolium intermediate **III** followed by liberation of the hydroperoxy anion. The intermediate **III** further undergo nucleophilic attack by deprotonated *N*-tosyl hydrazone and followed by intramolecular anulation to give intemediate **IV**. The final intermediate releases NHC for the next catalytic cycle along with the desired poduct, e.g., phthalidyl sulfonohydrazone (**4f**). From our ESI-MS reaction kinetics spectrum (Figure S1─S3) of the on-going reaction among **1a**, **2f** and **3c**, symbolic ESI-MS peaks were detected for '*Breslow intermediate*' **I,** at e/m 306.0980, intermediate **II** (azolium hydroperoxy intermediate) and **IV** at e/m 360.0889 and 591.4156, and **4f** at e/m 421.1139.

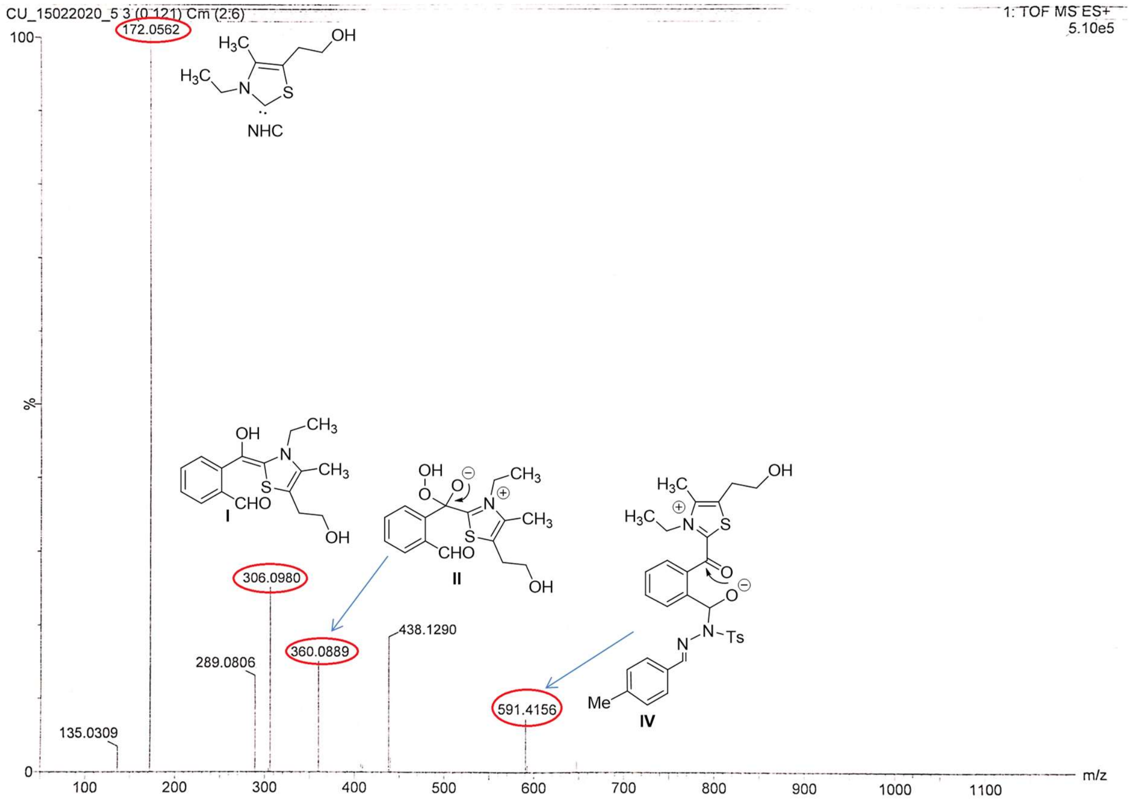

Figure S1. ESI-MS spectrum of the ongoing reaction after 2 min (intermediate **I**, **II** and **IV**).

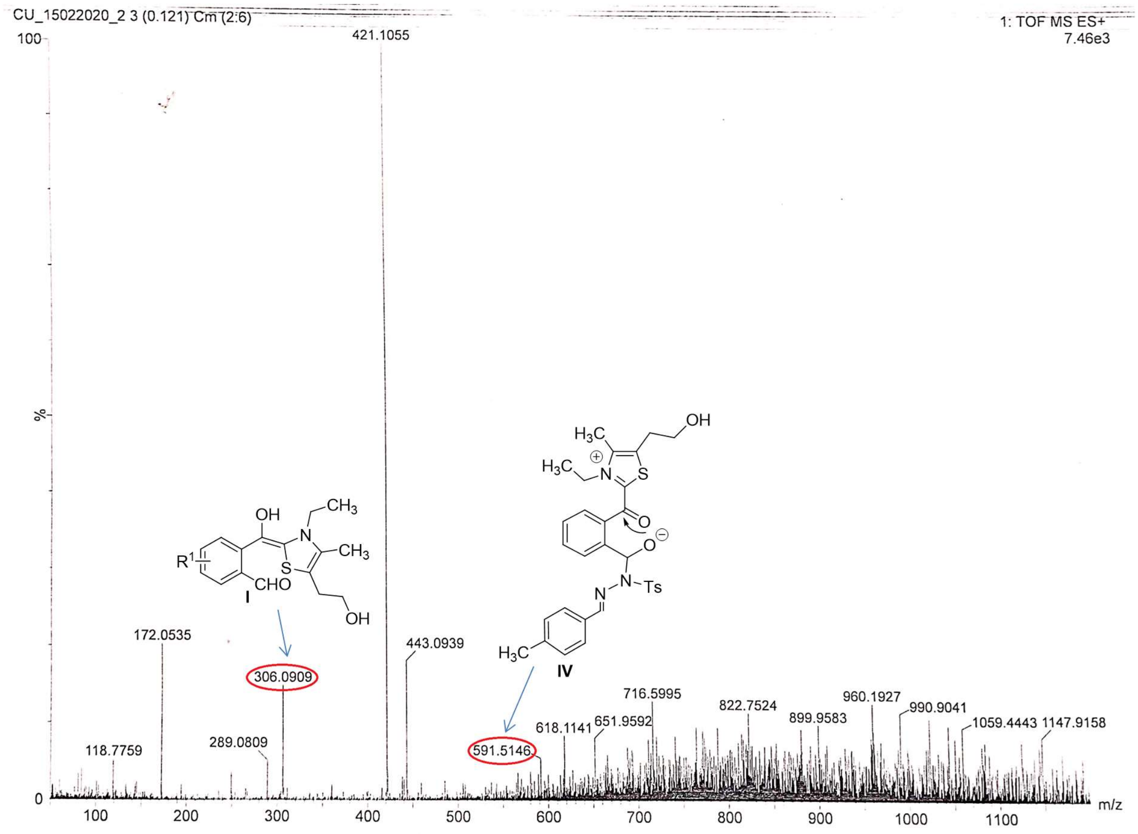

Figure S2. ESI-MS spectrum of the ongoing reaction after 3 min (intermediate **I** and **IV**).

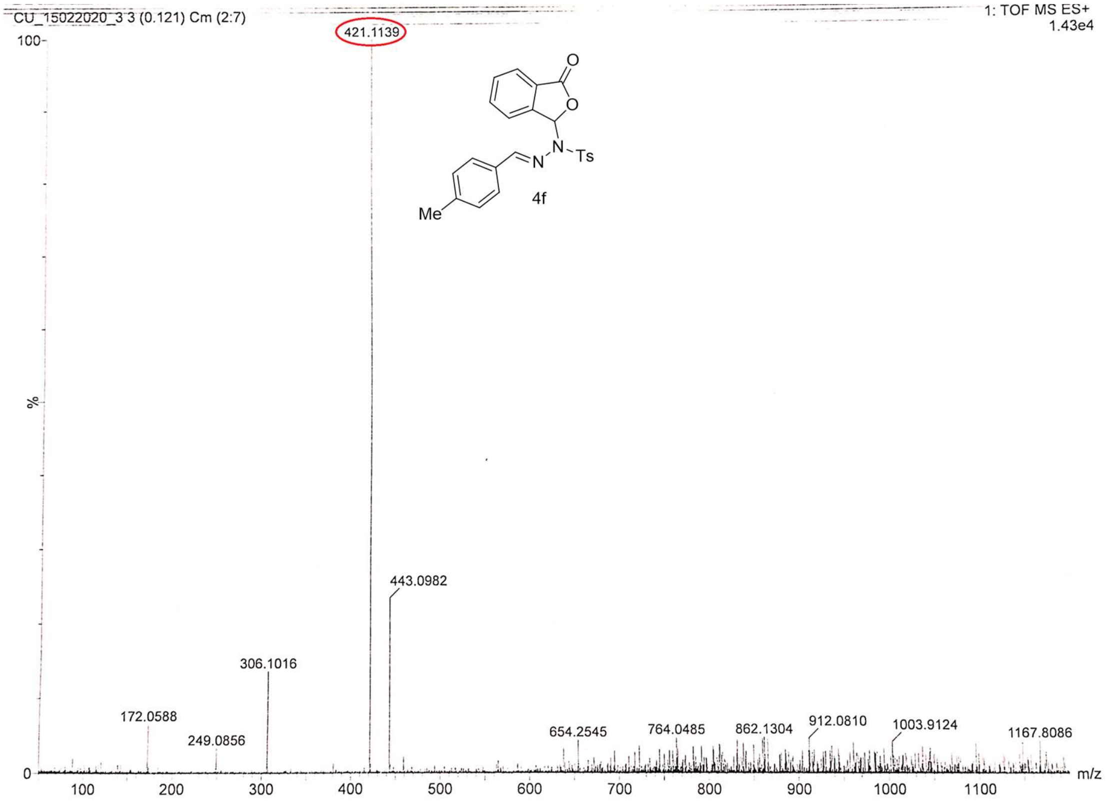

Figure S3. ESI-MS spectrum of the ongoing reaction after 30 min (compound **4f**).

2. ^{1}H and ^{13}C-NMR spectra of phthalidyl sulfonohydrazone derivatives

Figure S4. ^{1}H NMR (300 MHz) and ^{13}C NMR (75 MHz) spectra of compound 4a in CDCl$_3$.

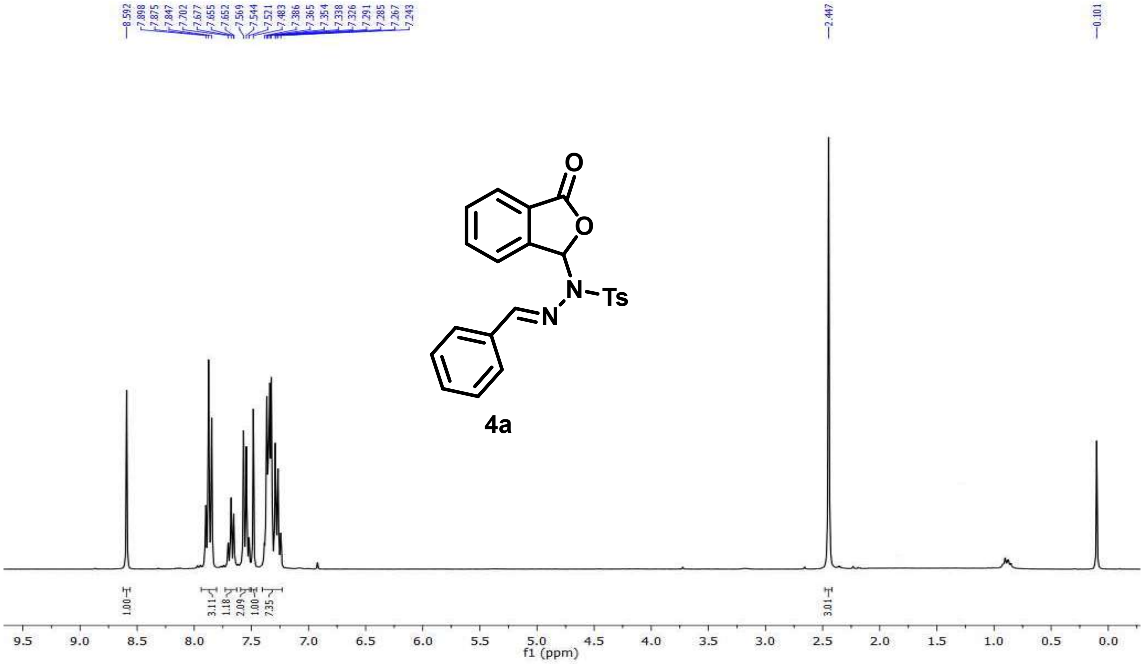

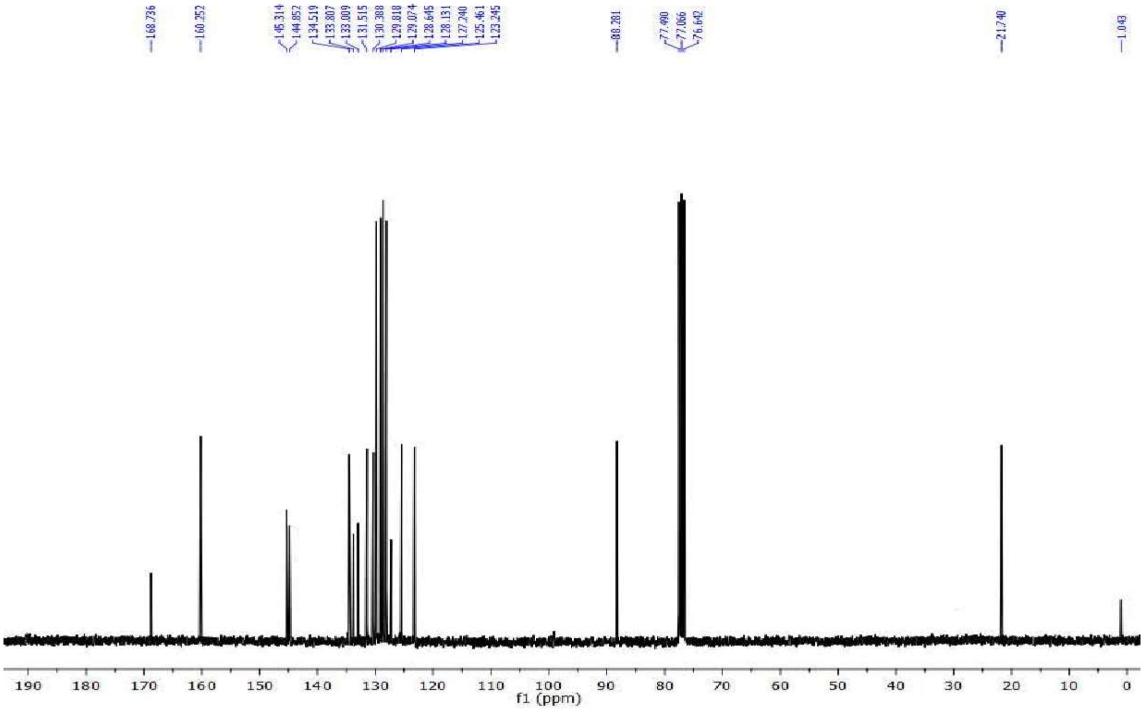

Figure S5. ^{1}H NMR (300 MHz) and ^{13}C NMR (75 MHz) spectra of compound 4b in CDCl$_3$.

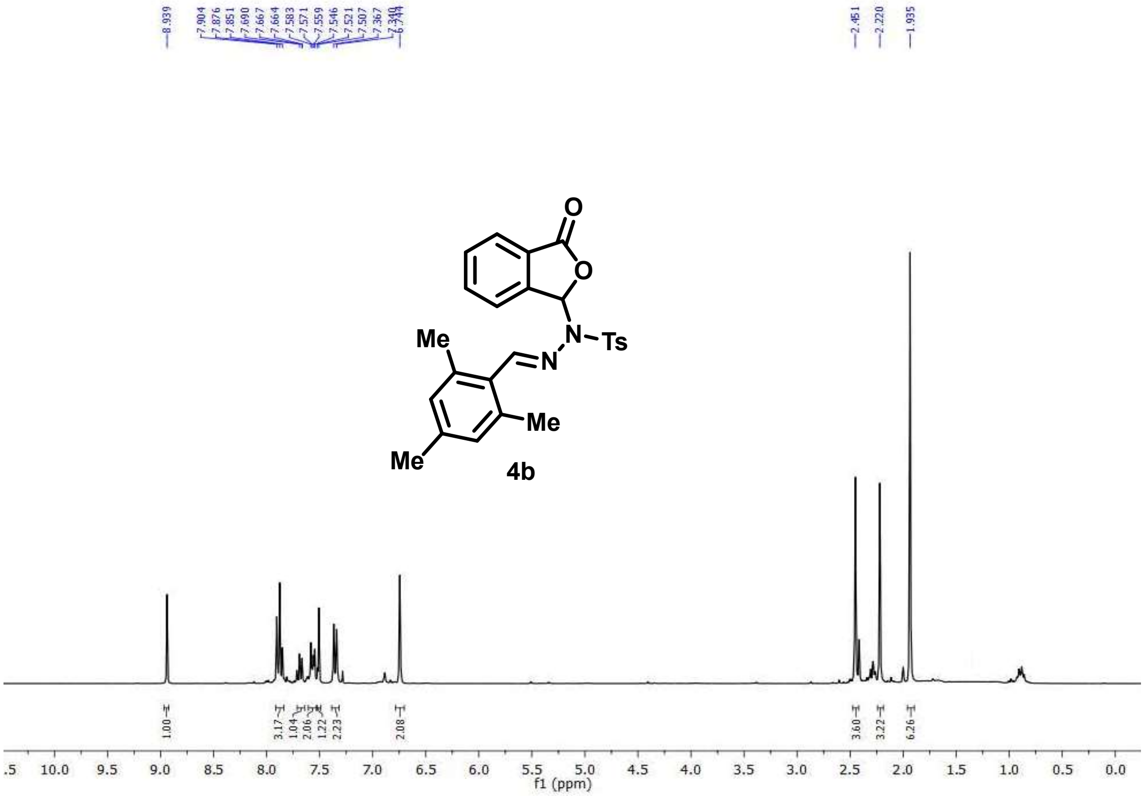

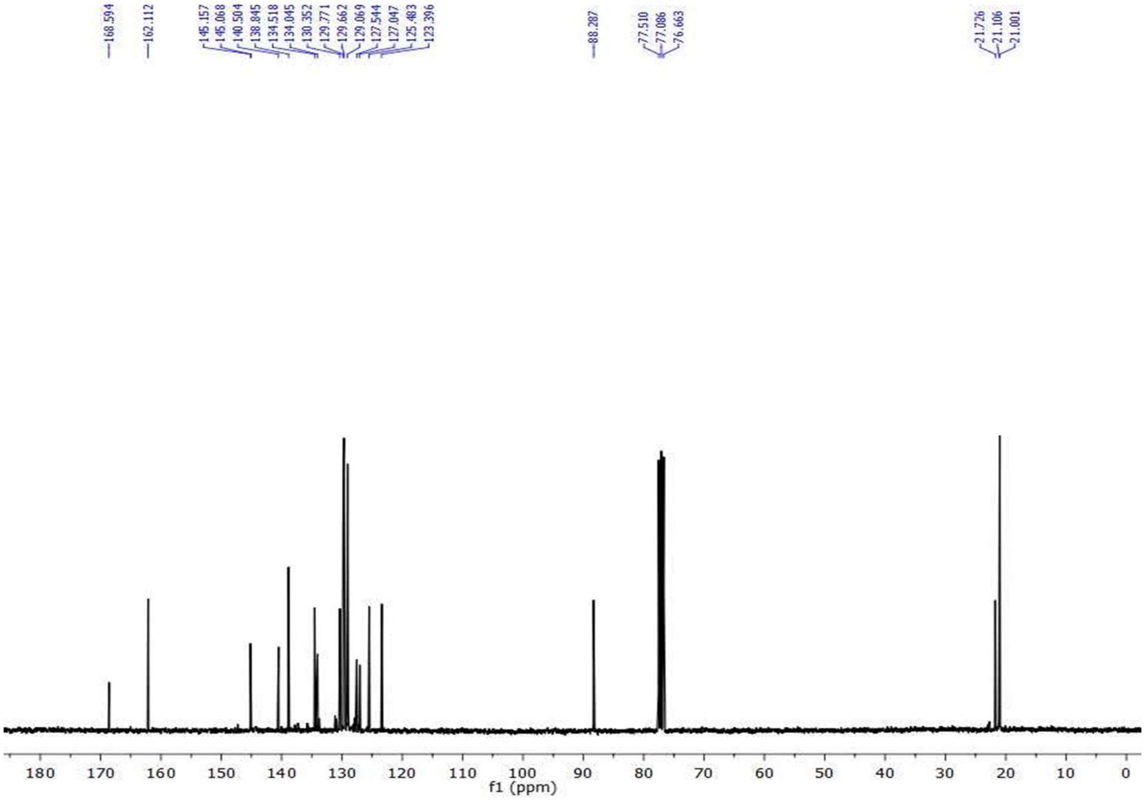

Figure S6. ^{1}**H NMR (300 MHz) and** ^{13}**C NMR (75 MHz) spectra of compound 4c in** $CDCl_3$.

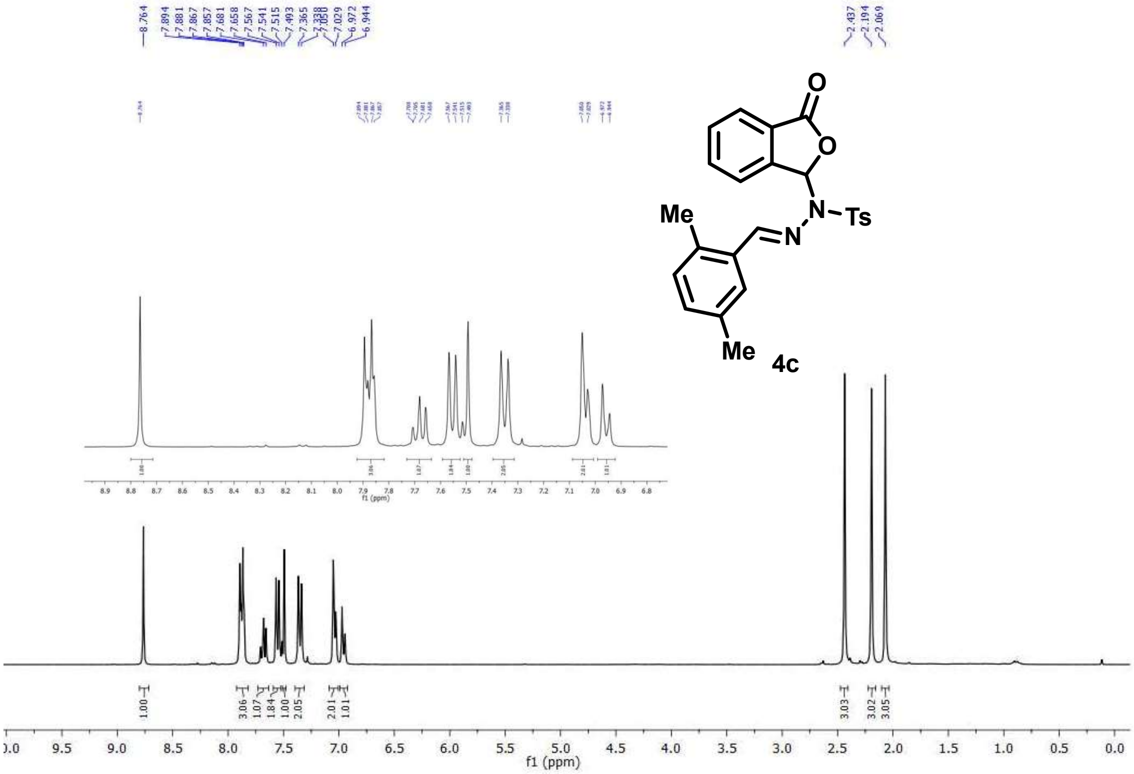

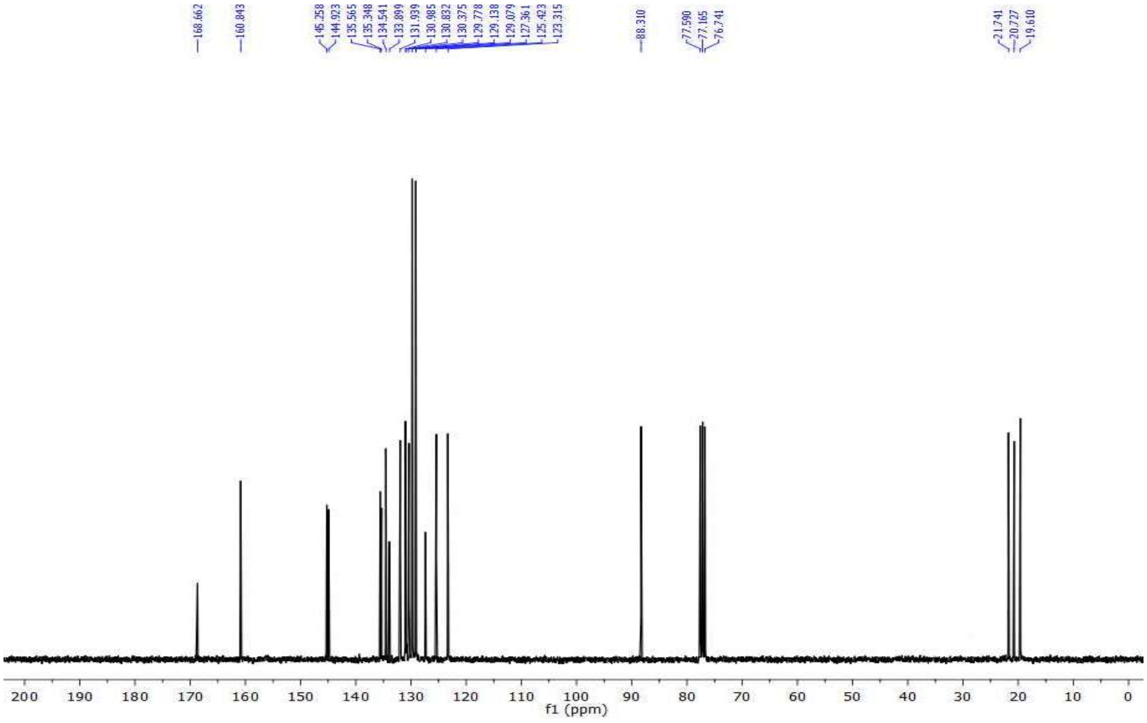

Figure S7. ^{1}H **NMR (300 MHz) and** ^{13}C **NMR (75 MHz) spectra of compound 4d in** $CDCl_3$.

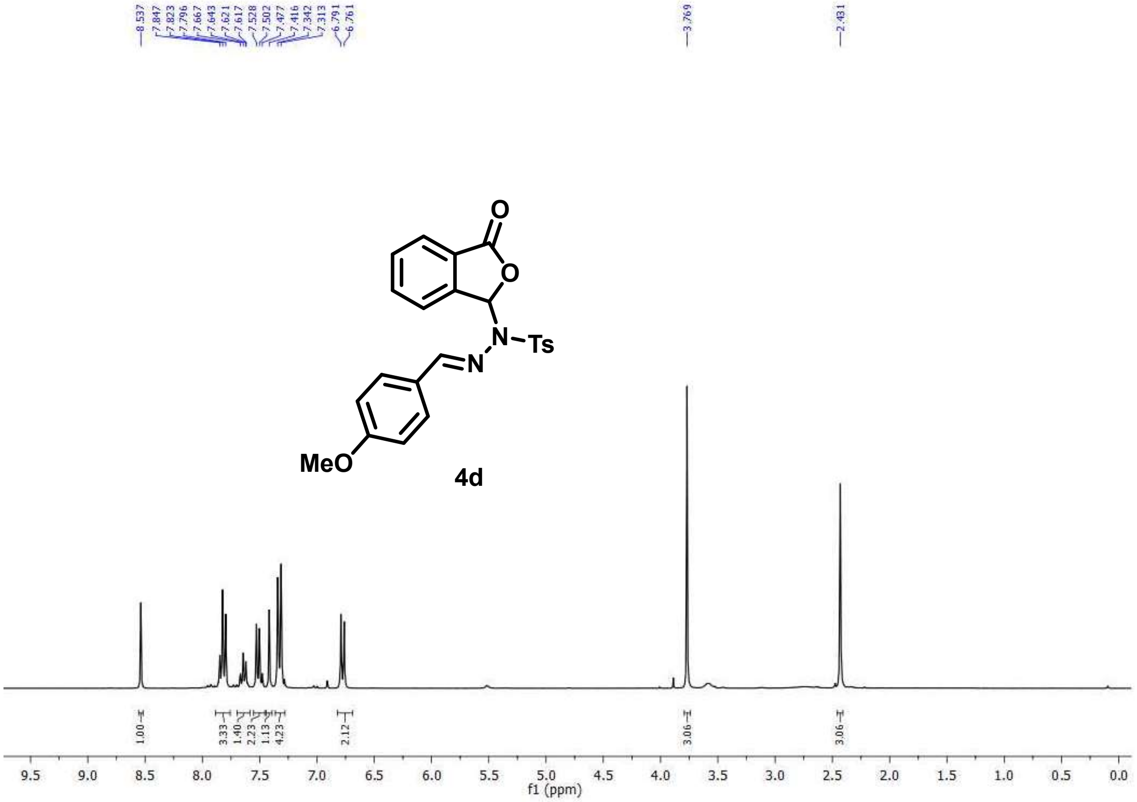

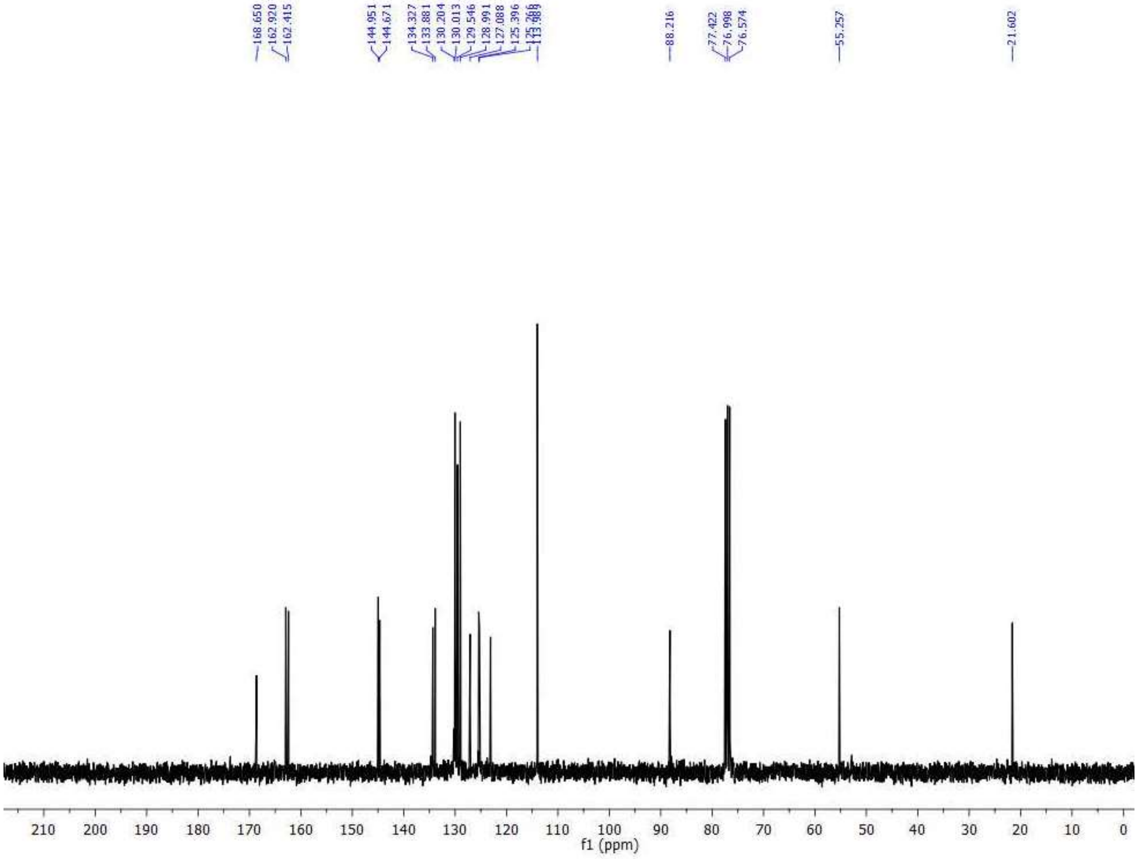

Figure S8. ^1H NMR (300 MHz) and ^{13}C NMR (75 MHz) spectra of compound 4e in $CDCl_3$.

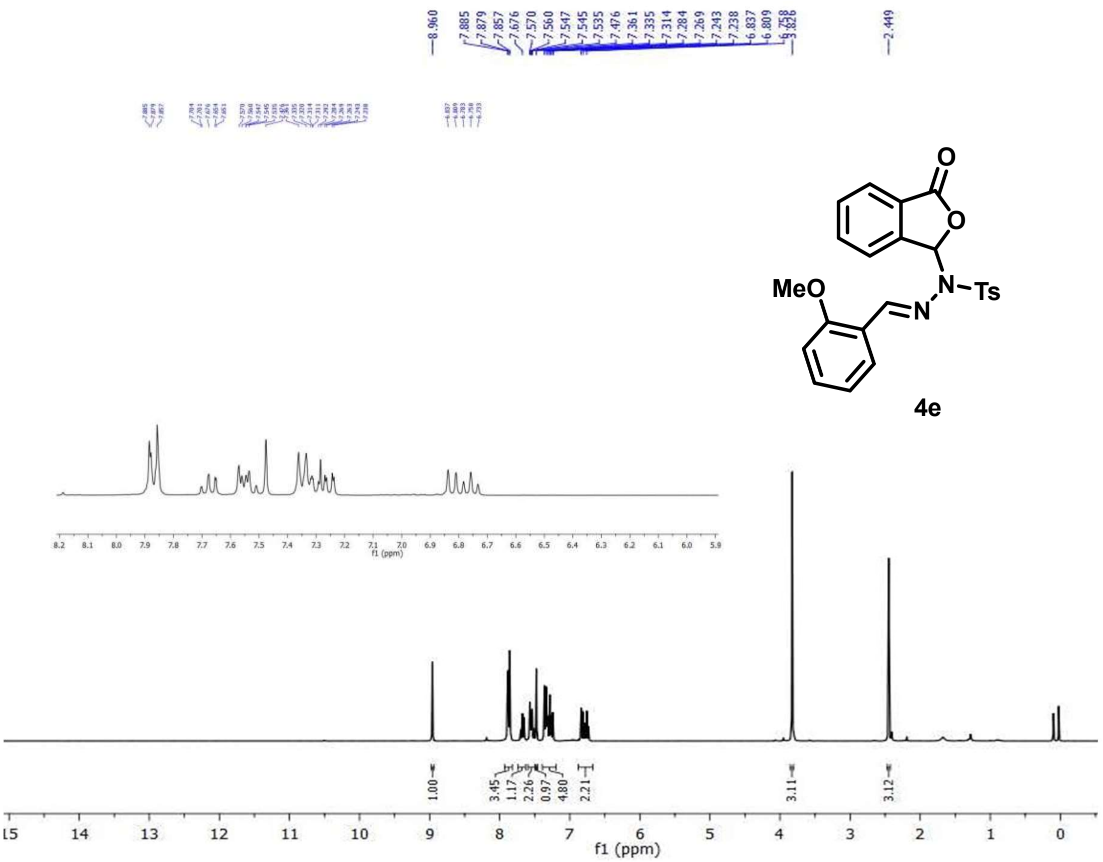

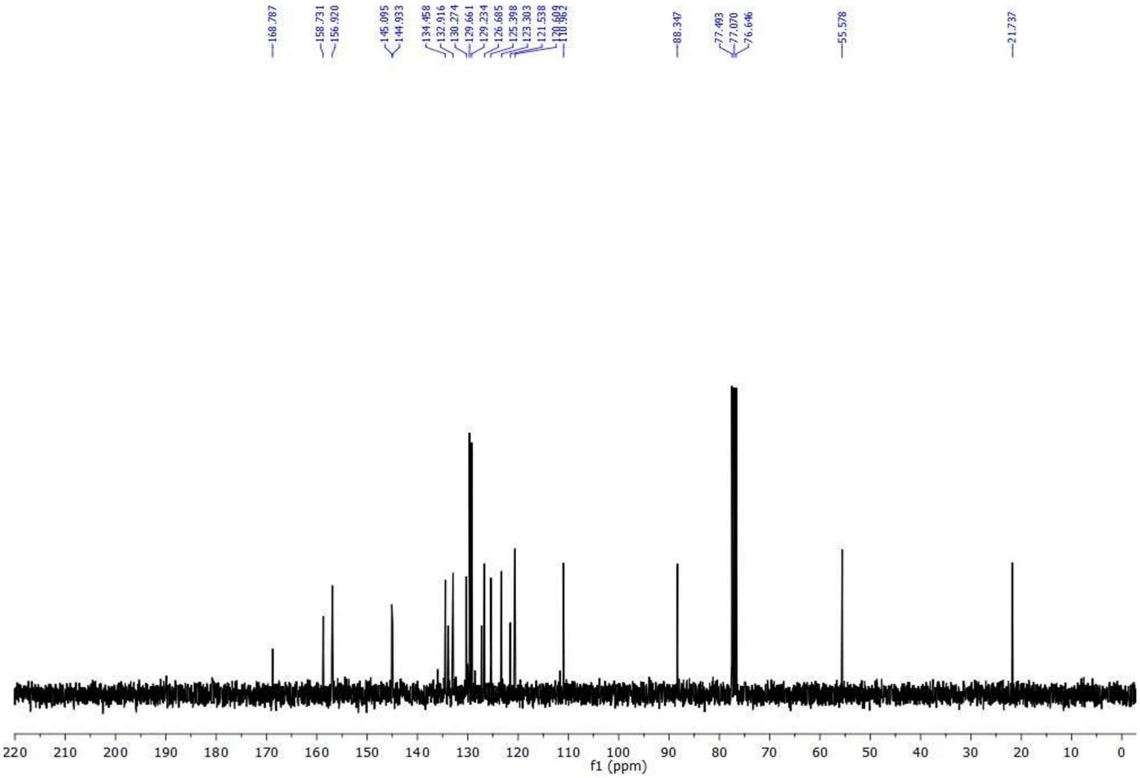

Figure S9. ^{1}H **NMR (300 MHz) and** ^{13}C **NMR (75 MHz) spectra of compound 4f in** $CDCl_3$**.**

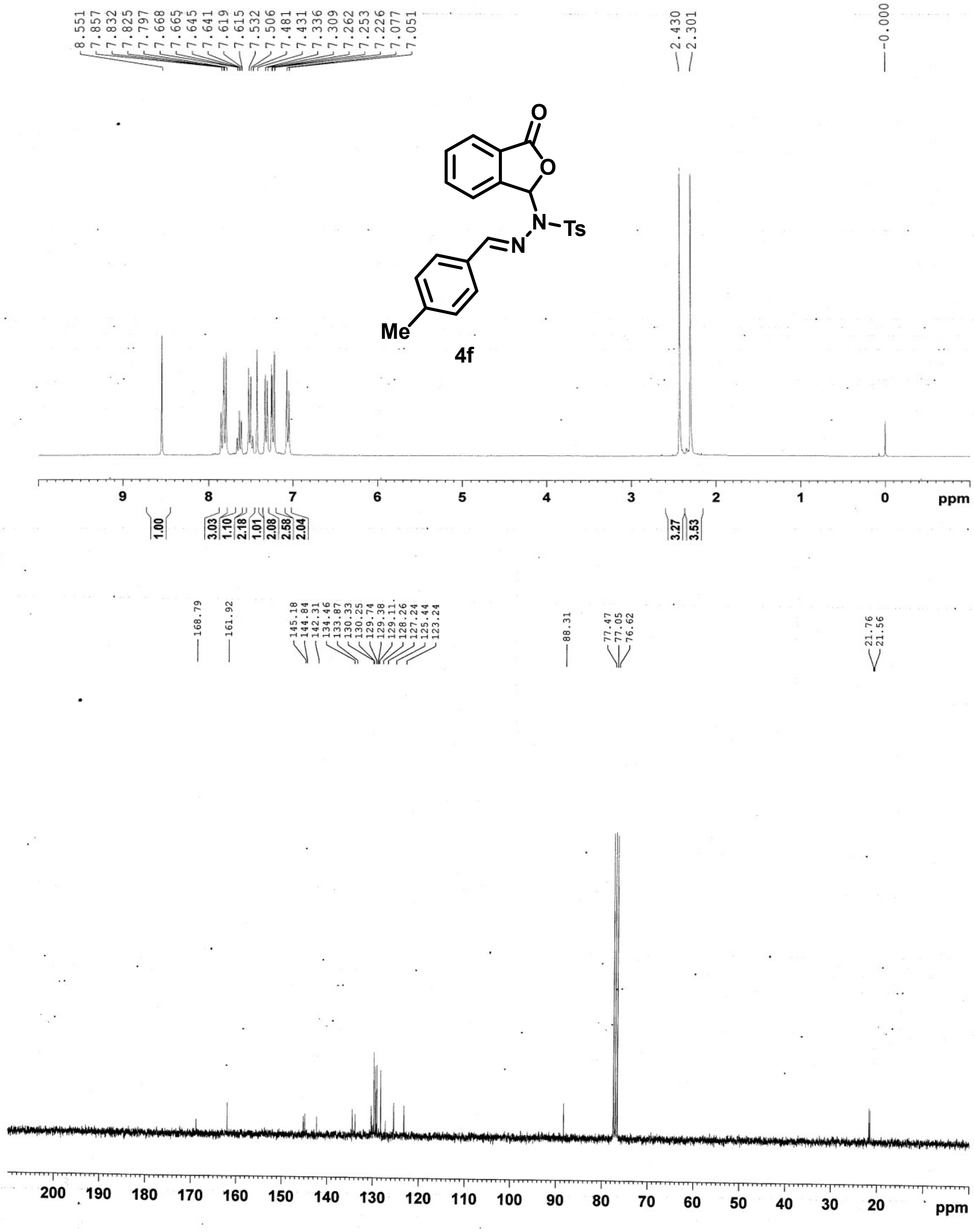

Figure S10. ¹H NMR (300 MHz) and ¹³C NMR (75 MHz) spectra of compound 4g in $CDCl_3$.

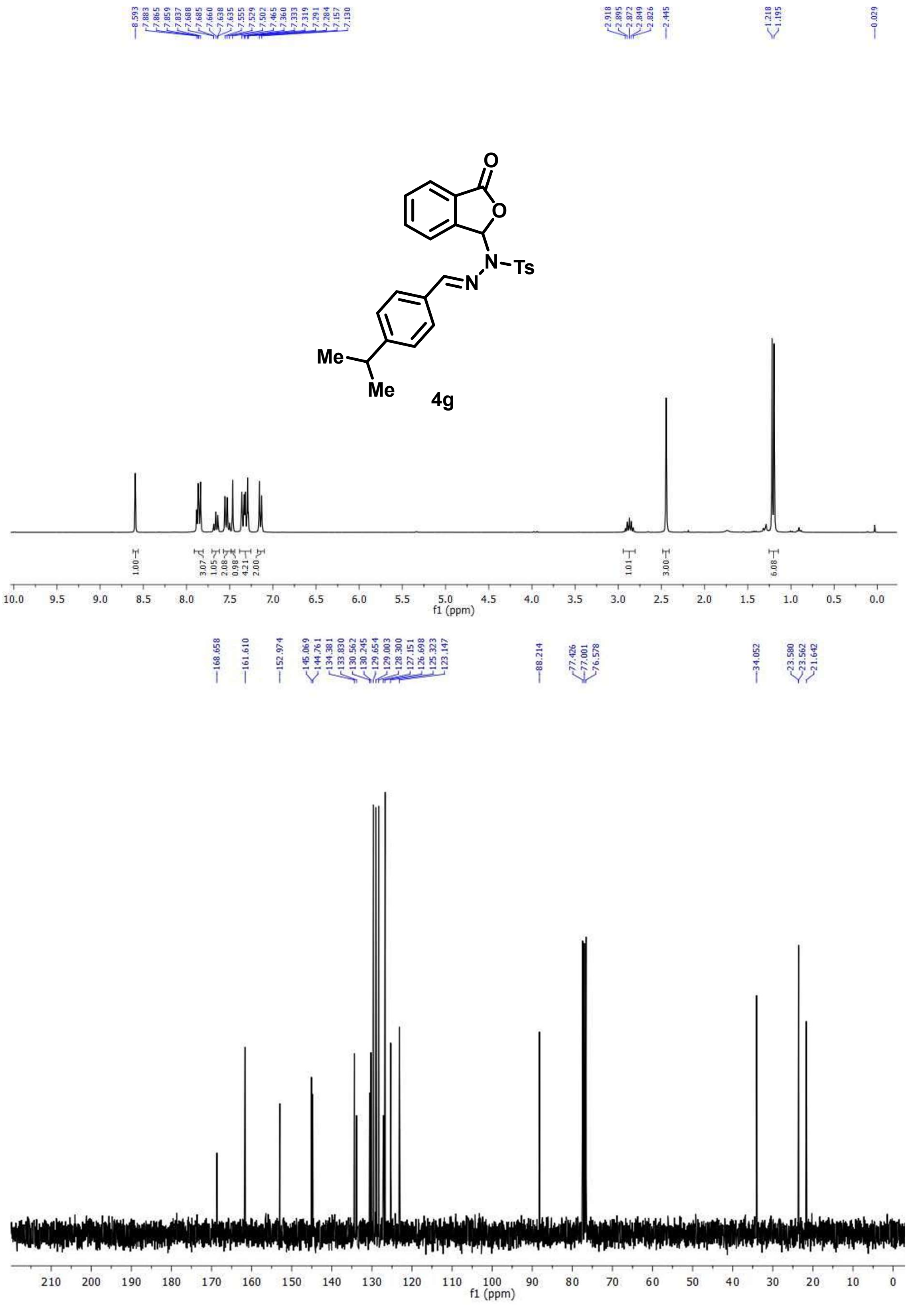

Figure S11. ^{1}H NMR (300 MHz) and ^{13}C NMR (75 MHz) spectra of compound 4h in $CDCl_3$.

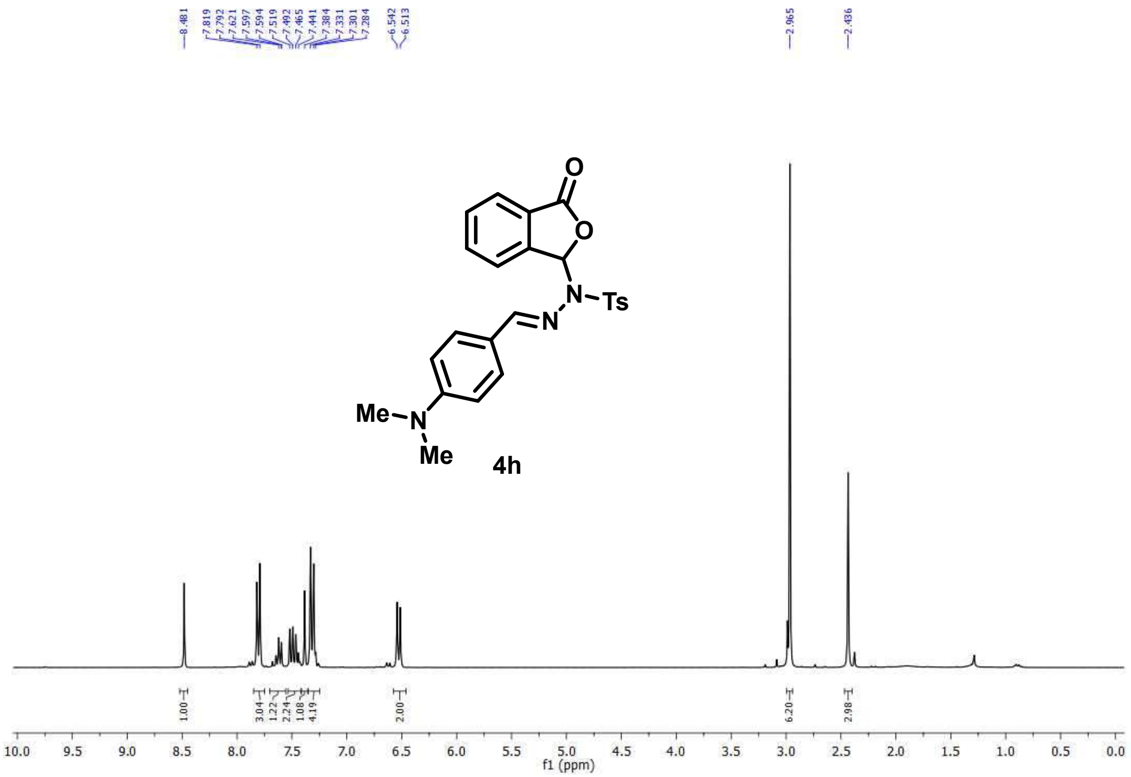

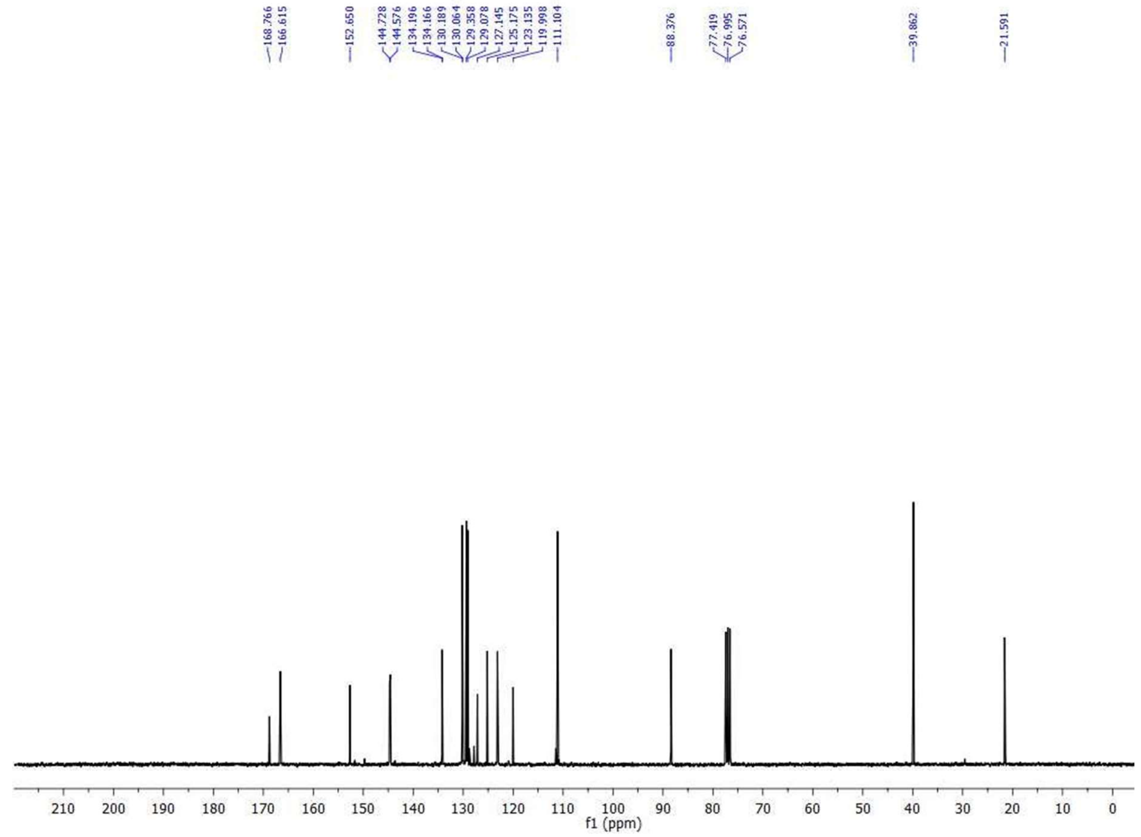

Figure S12. ^{1}H **NMR (300 MHz) and** ^{13}C **NMR (75 MHz) spectra of compound 4i in** $CDCl_3$.

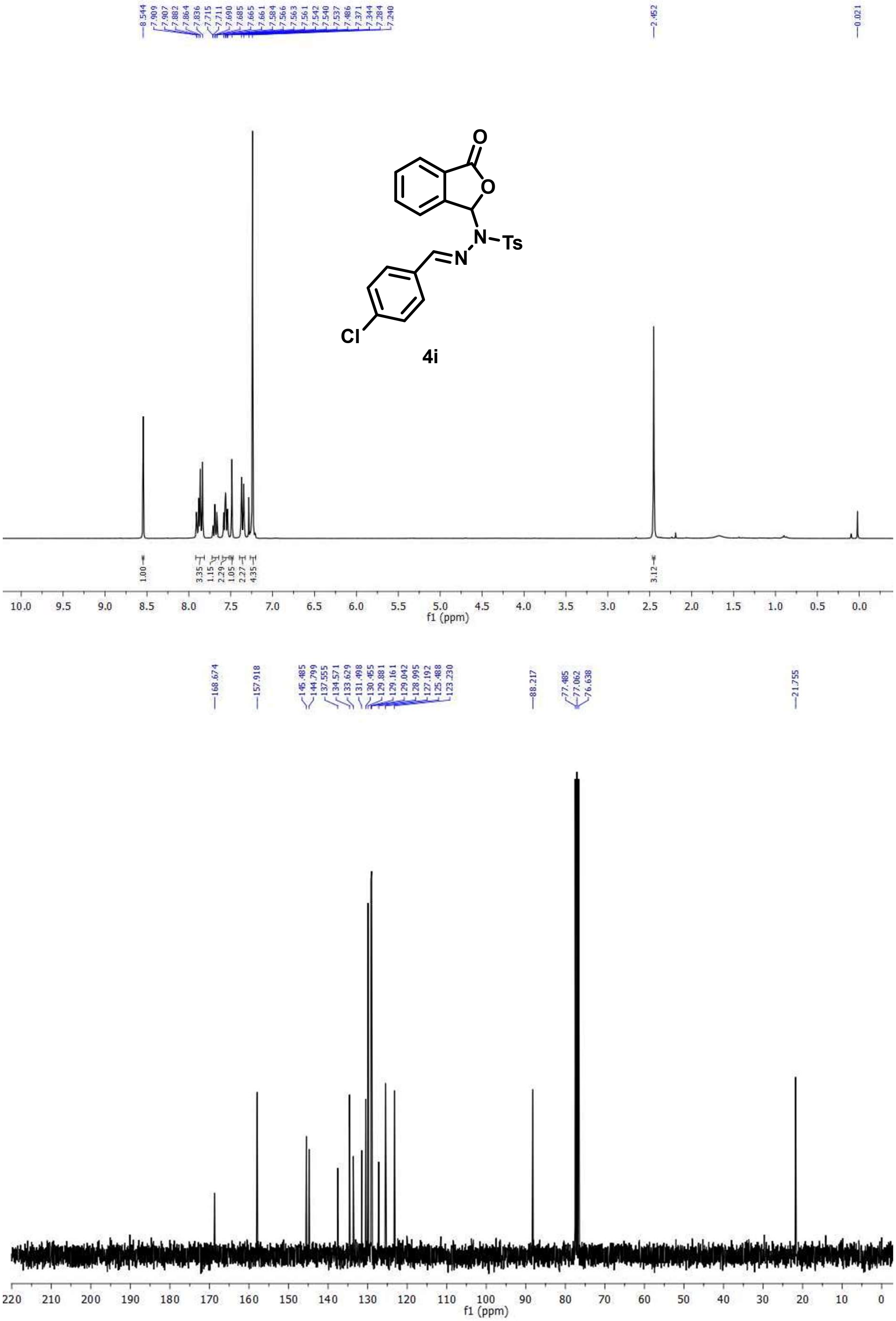

Figure S13. ^{1}H NMR (300 MHz) and ^{13}C NMR (75 MHz) spectra of compound 4j in $CDCl_3$.

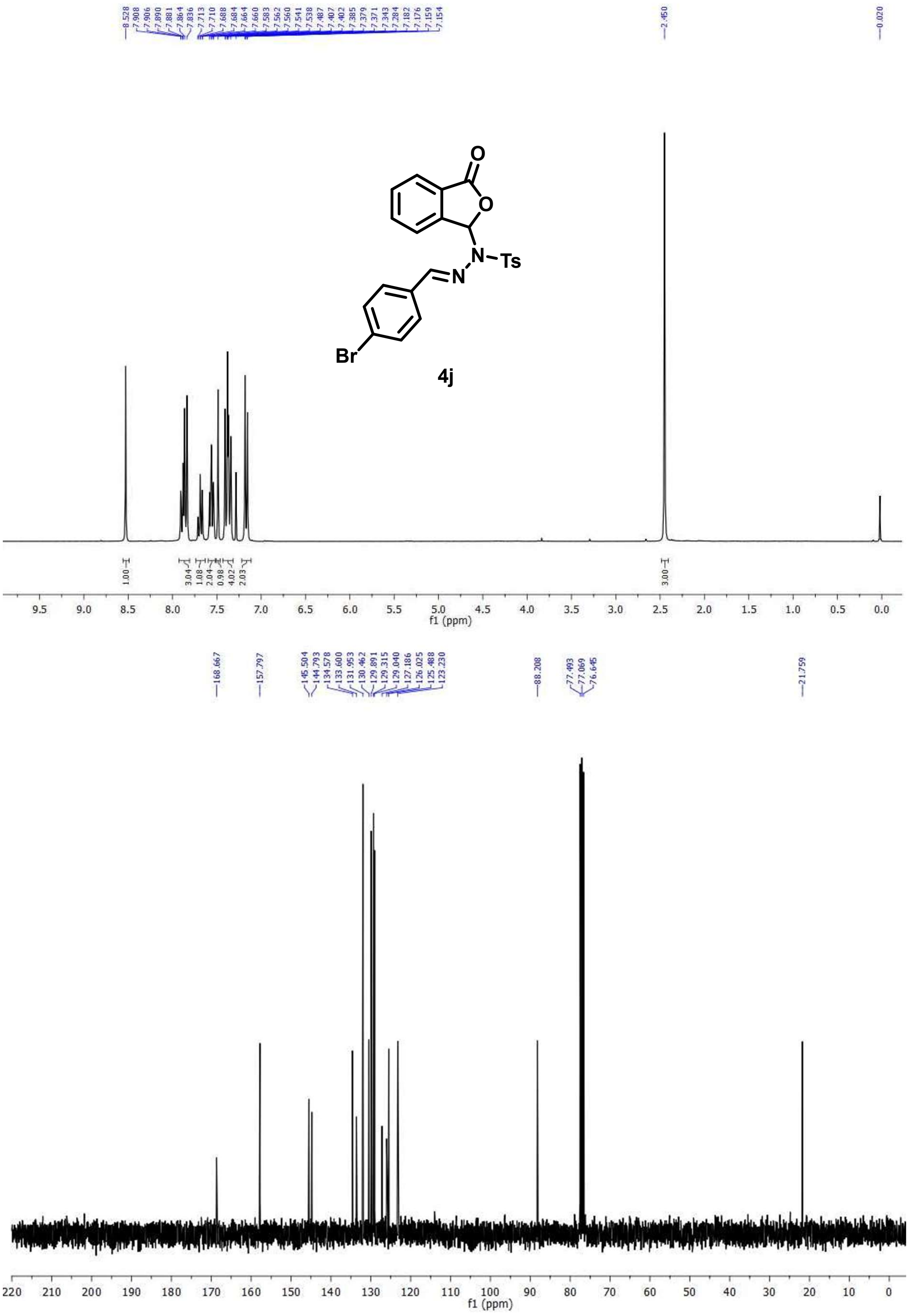

Figure S14. ^{1}H NMR (300 MHz) and ^{13}C NMR (75 MHz) spectra of compound 4k in $CDCl_3$.

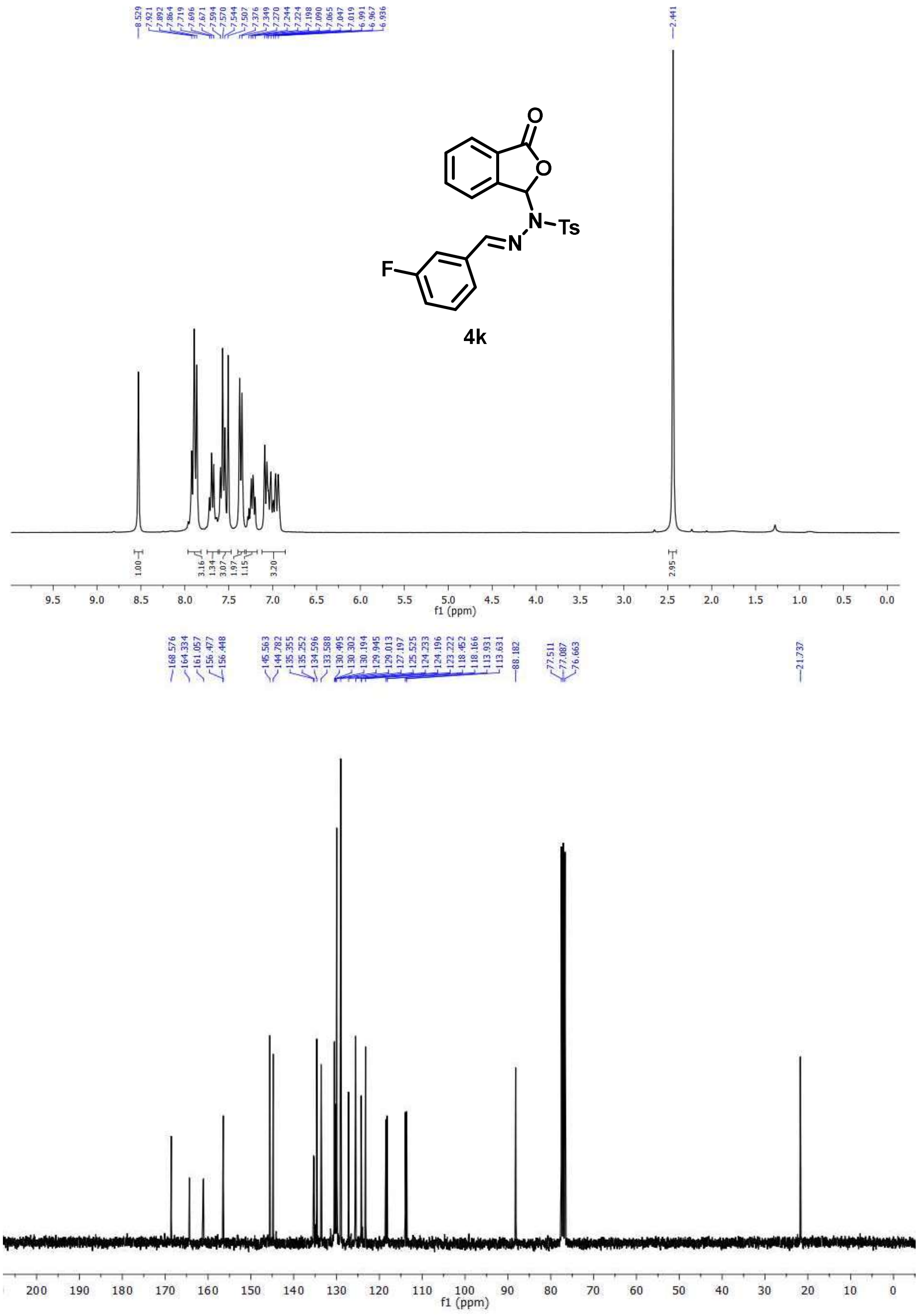

Figure S15. ^{1}H NMR (300 MHz) and ^{13}C NMR (75 MHz) spectra of compound 4l in $CDCl_3$.

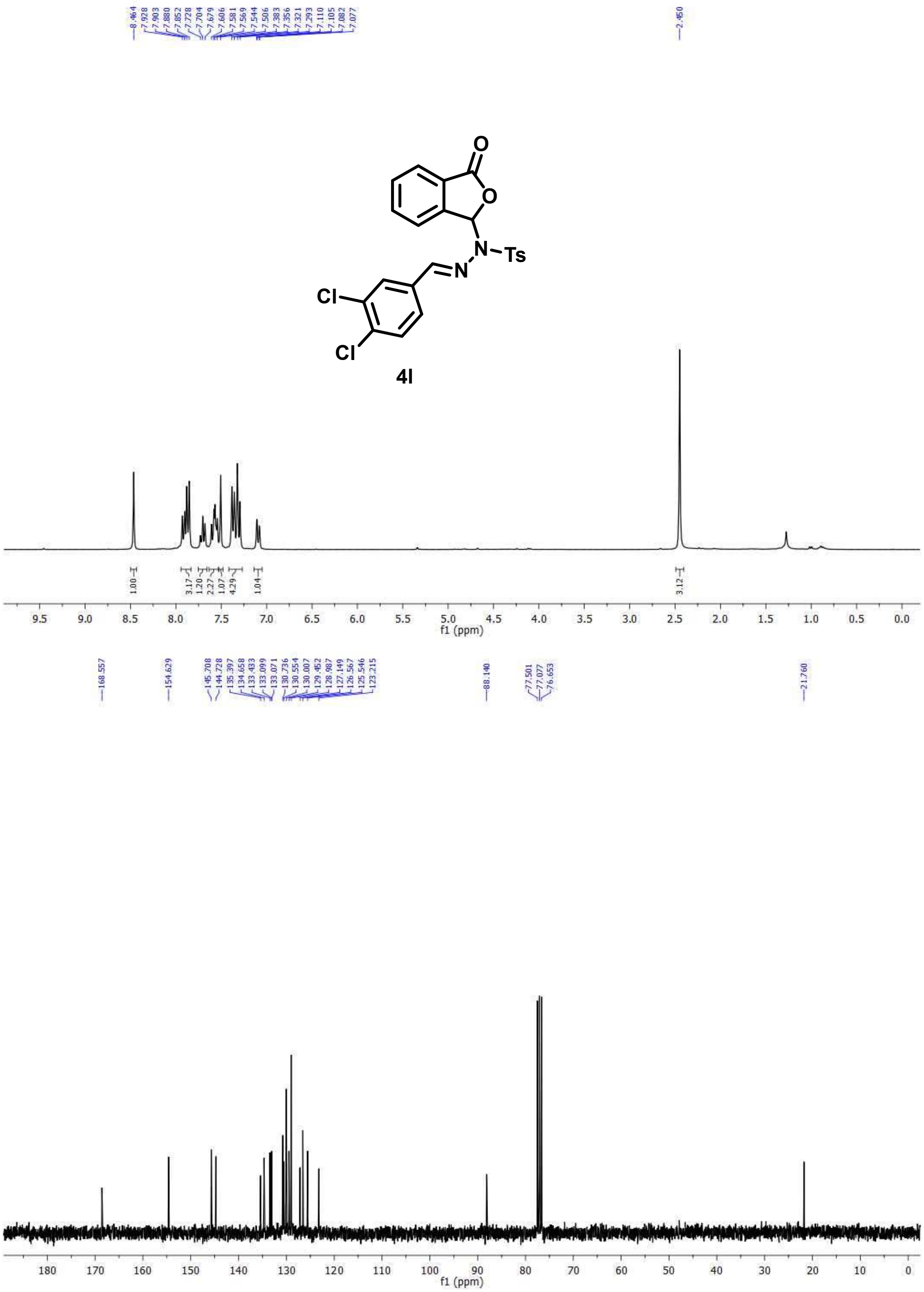

Figure S16. ^{1}H NMR (300 MHz) and ^{13}C NMR (75 MHz) spectra of compound 4m in $CDCl_3$.

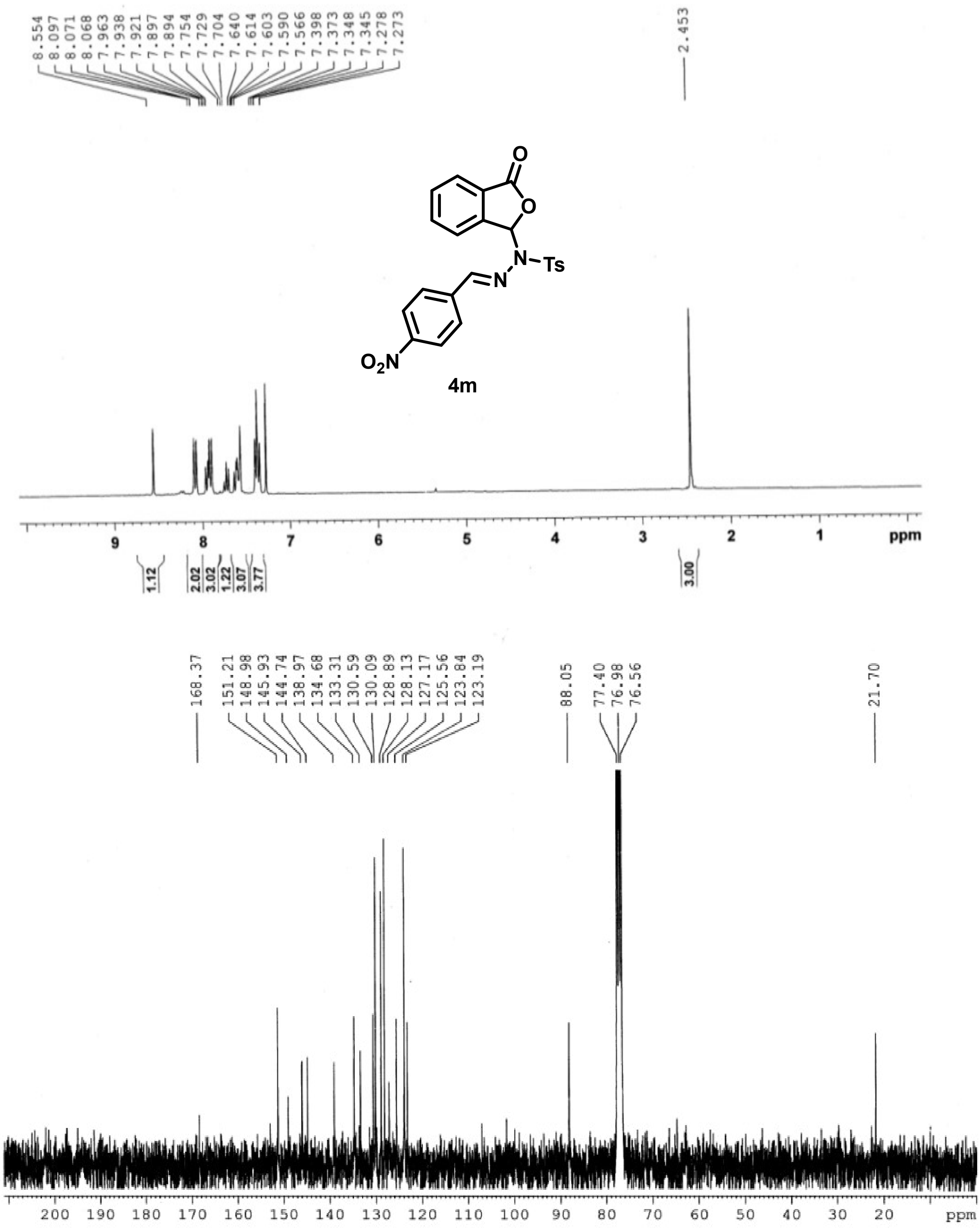

Figure S17. ^{1}H NMR (300 MHz) and ^{13}C NMR (75 MHz) spectra of compound 4n in $CDCl_3$.

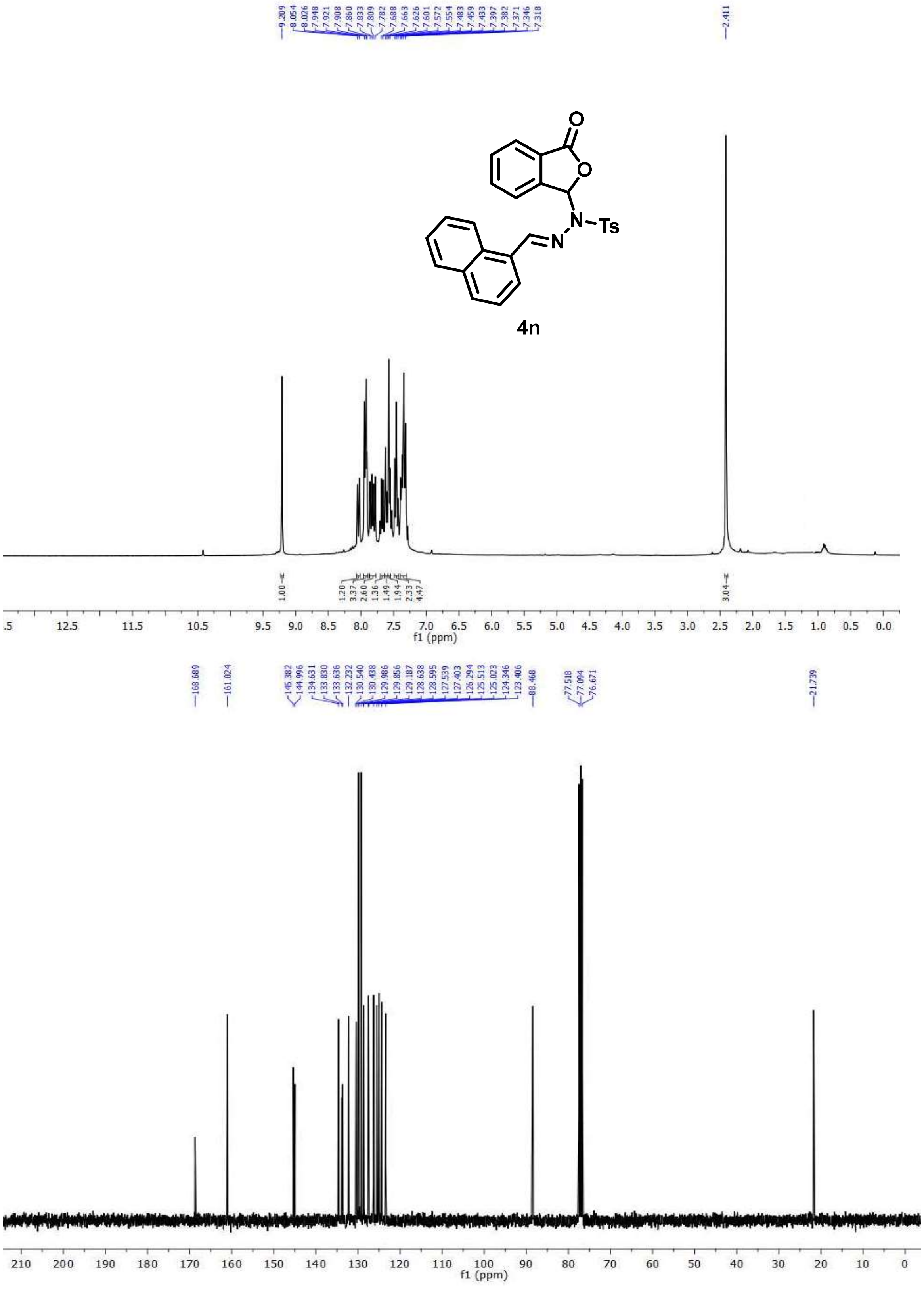

Figure S18. ^{1}H NMR (300 MHz) and ^{13}C NMR (75 MHz) spectra of compound 4o in $CDCl_3$.

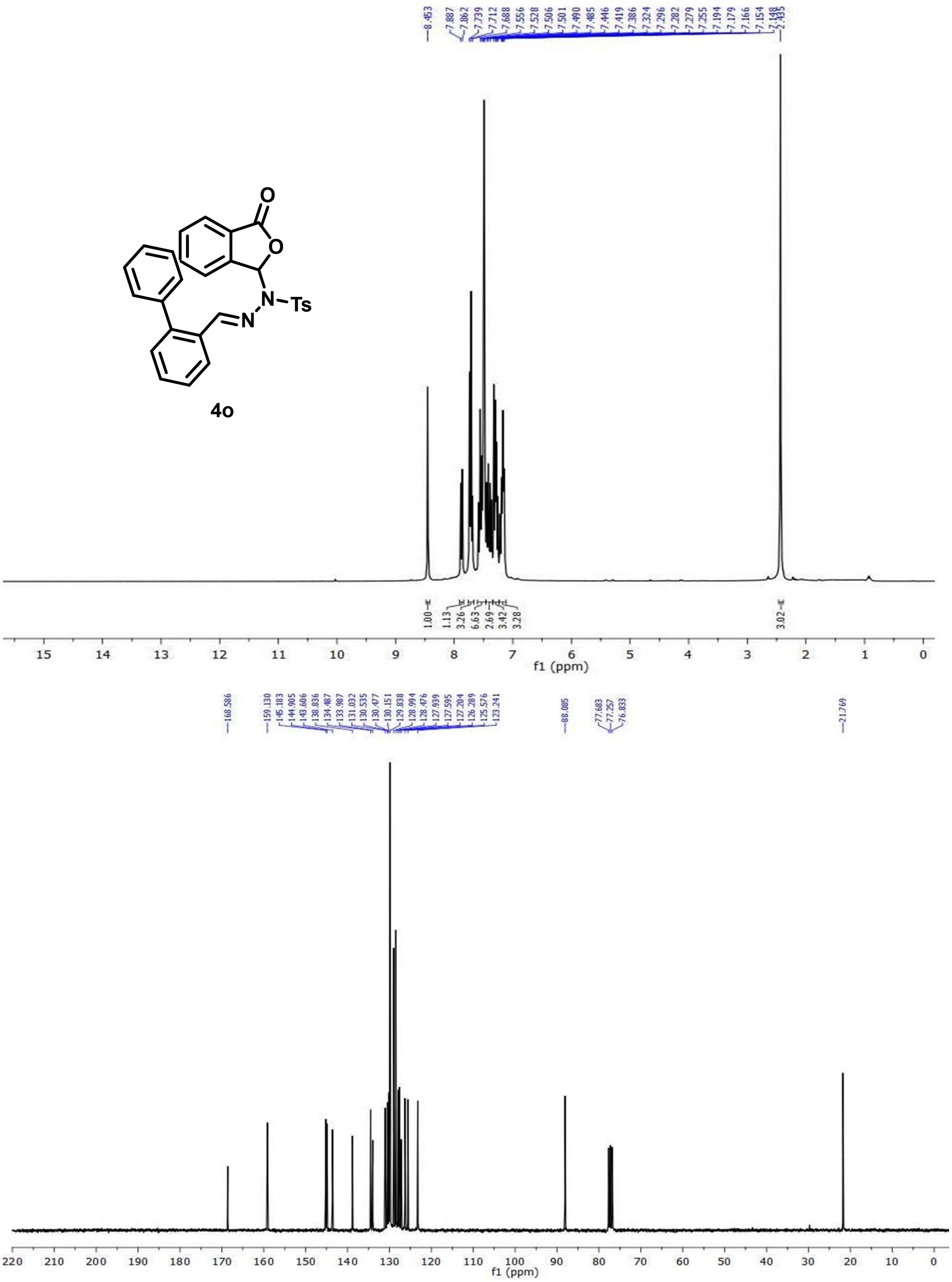

Figure S19. ^{1}H NMR (300 MHz) and ^{13}C NMR (75 MHz) spectra of compound 4p in $CDCl_3$.

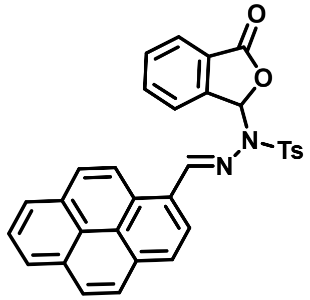

4p

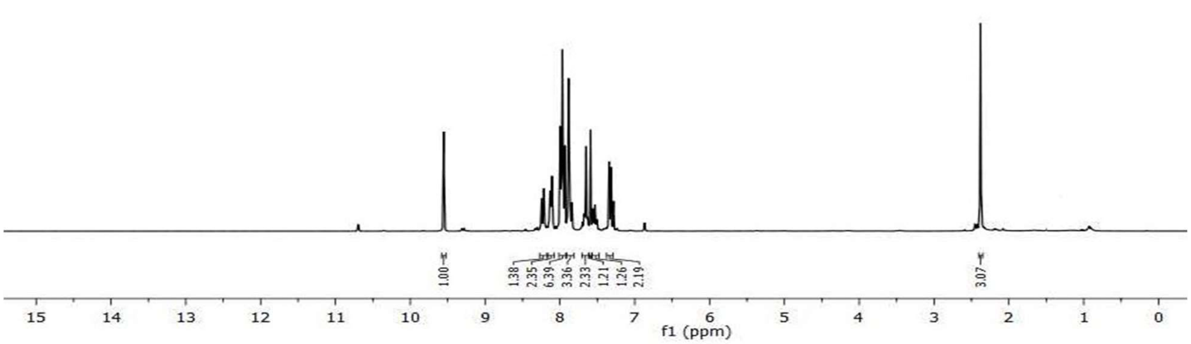

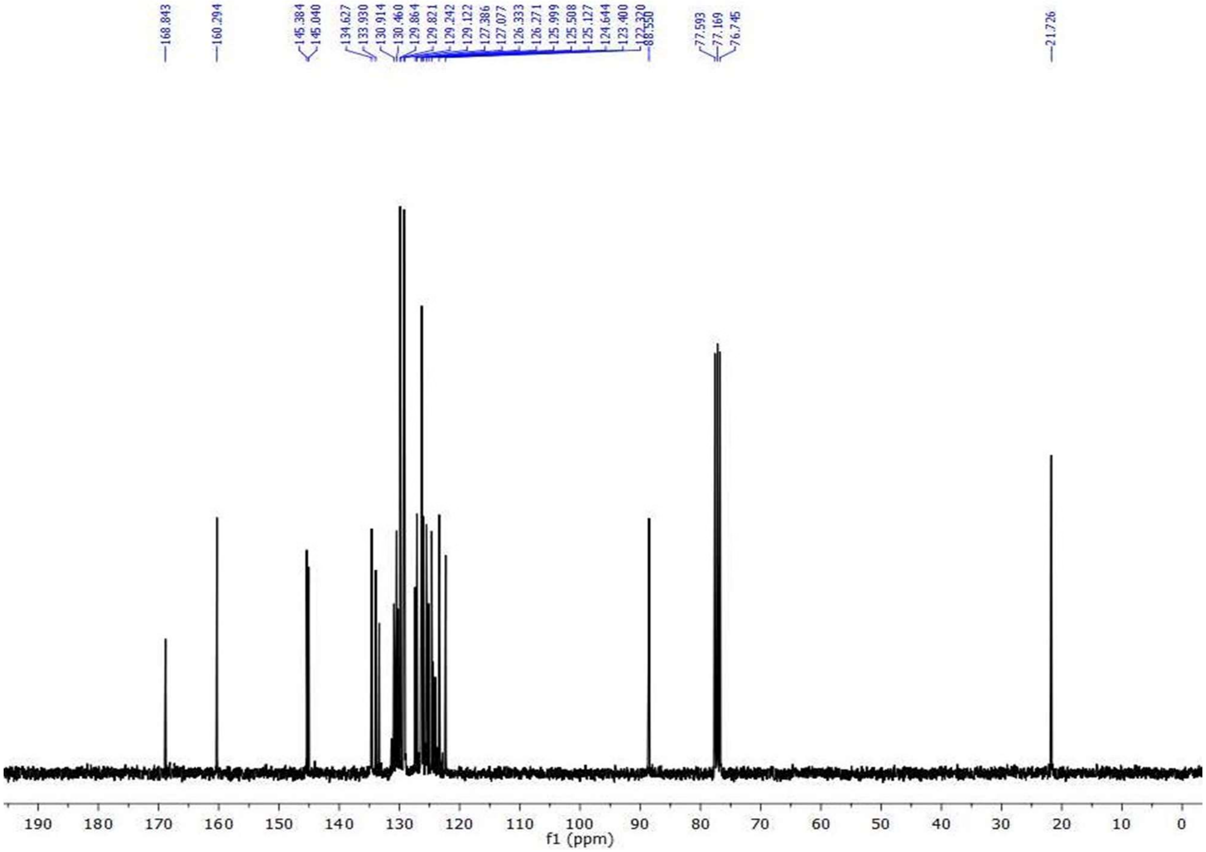

Figure S20. ^{1}H NMR (300 MHz) and ^{13}C NMR (75 MHz) spectra of compound 4q in $CDCl_3$.

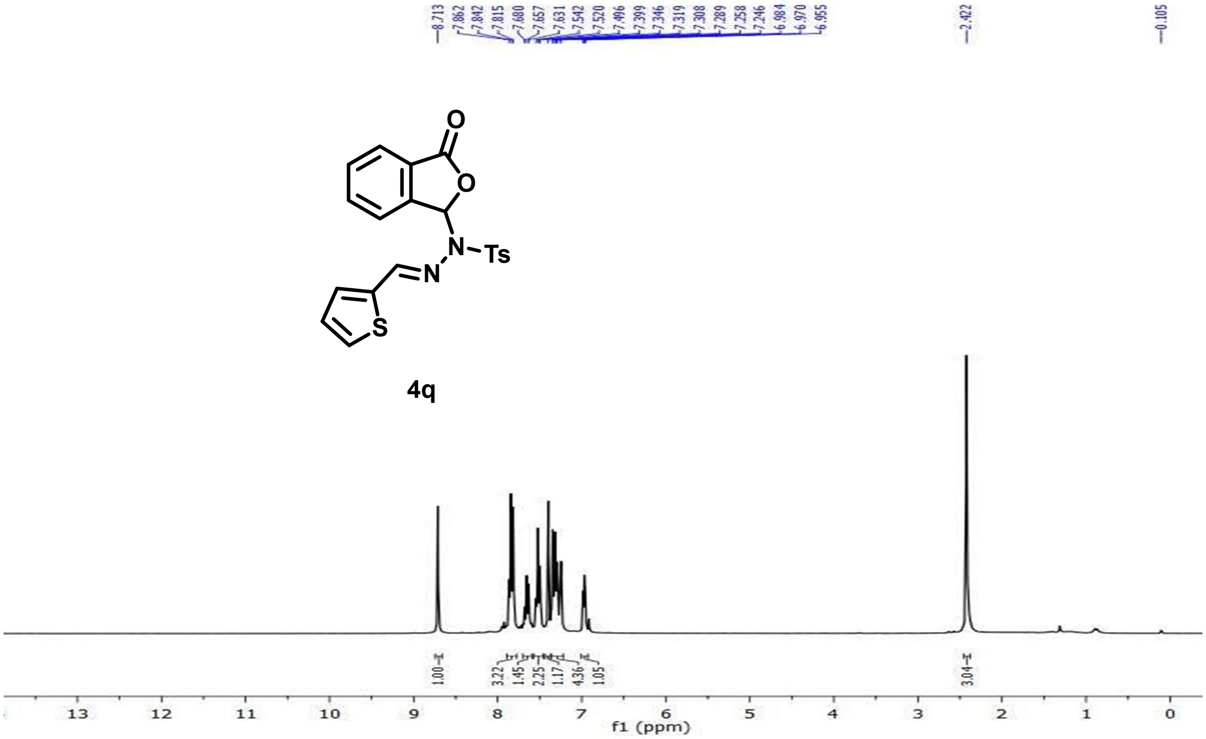

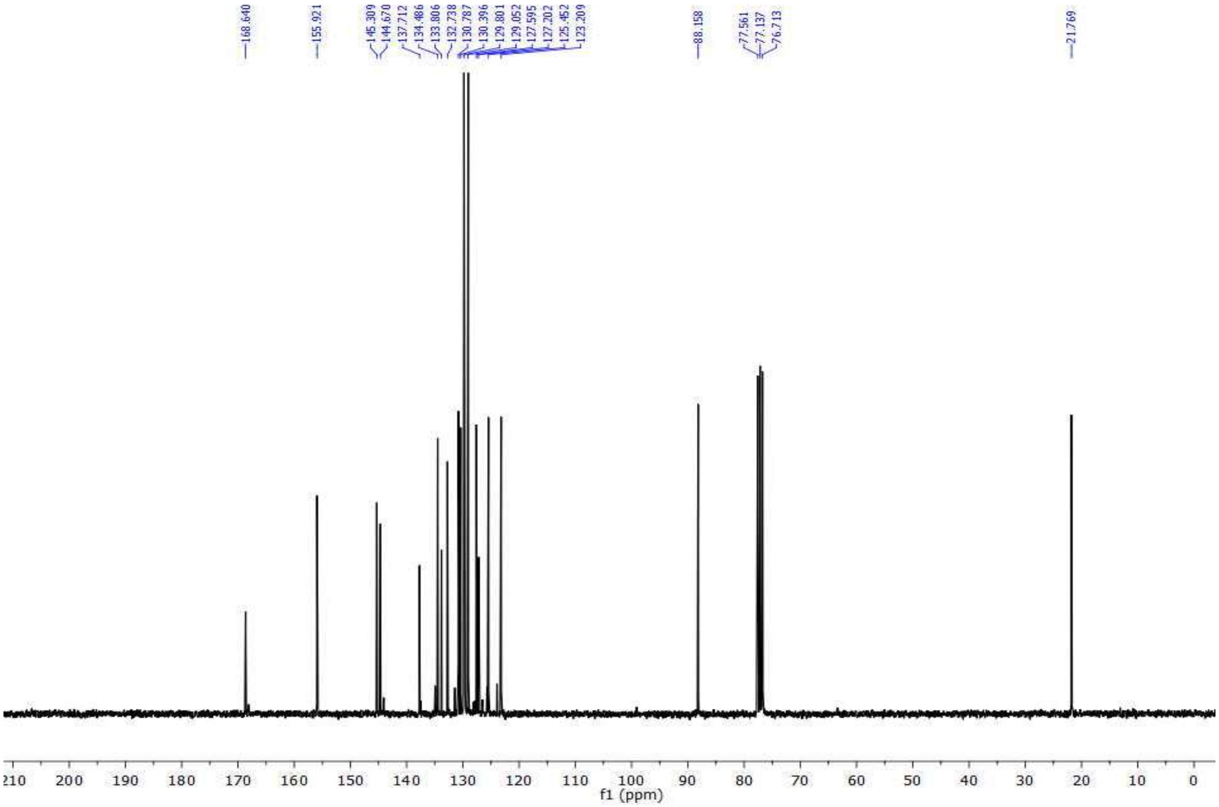

Figure S21. ^{1}H NMR (300 MHz) and ^{13}C NMR (75 MHz) spectra of compound 4r in $CDCl_3$.

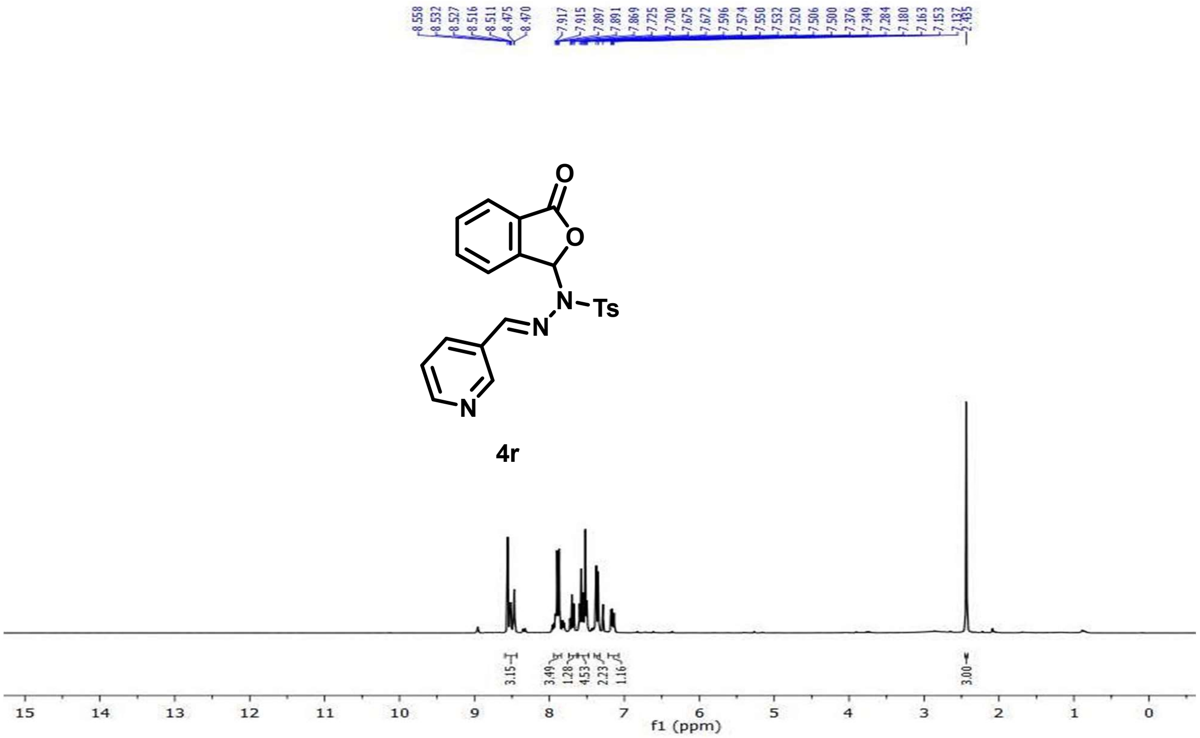

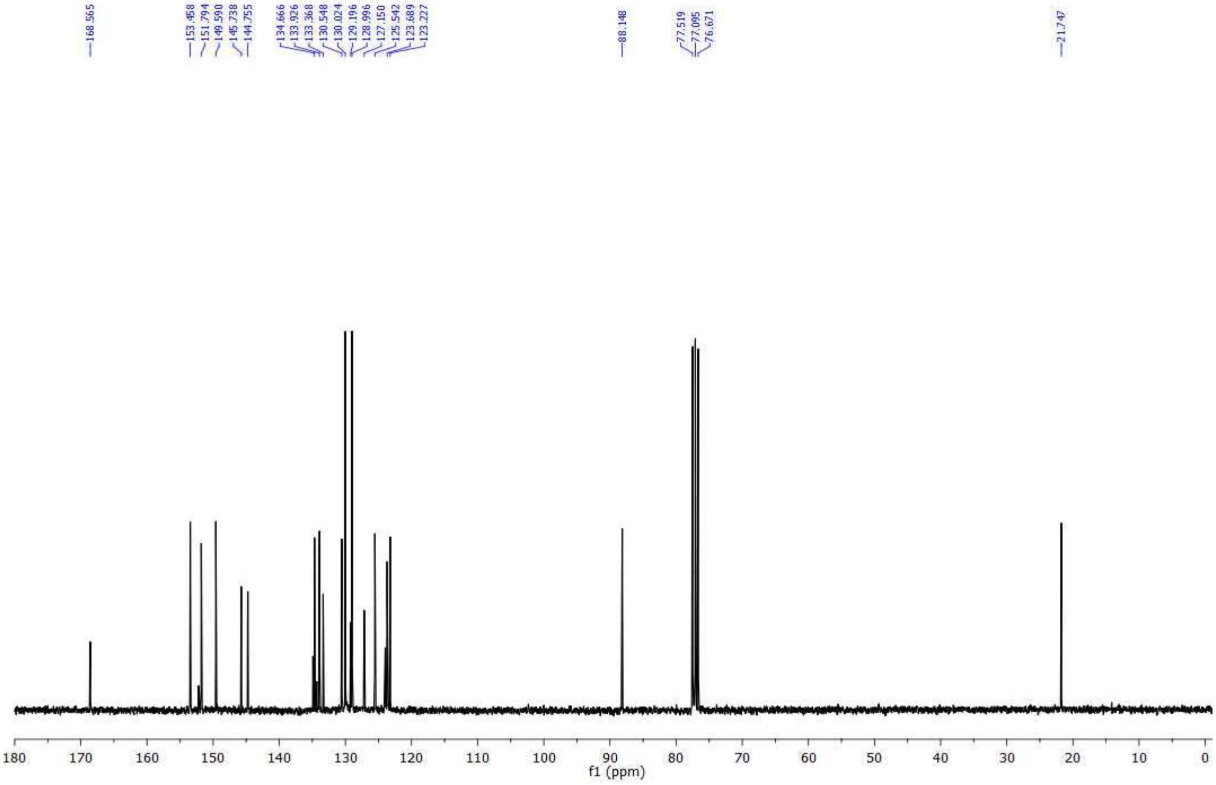

Figure S22. ^1H NMR (300 MHz) and ^{13}C NMR (75 MHz) spectra of compound 4s in $CDCl_3$.

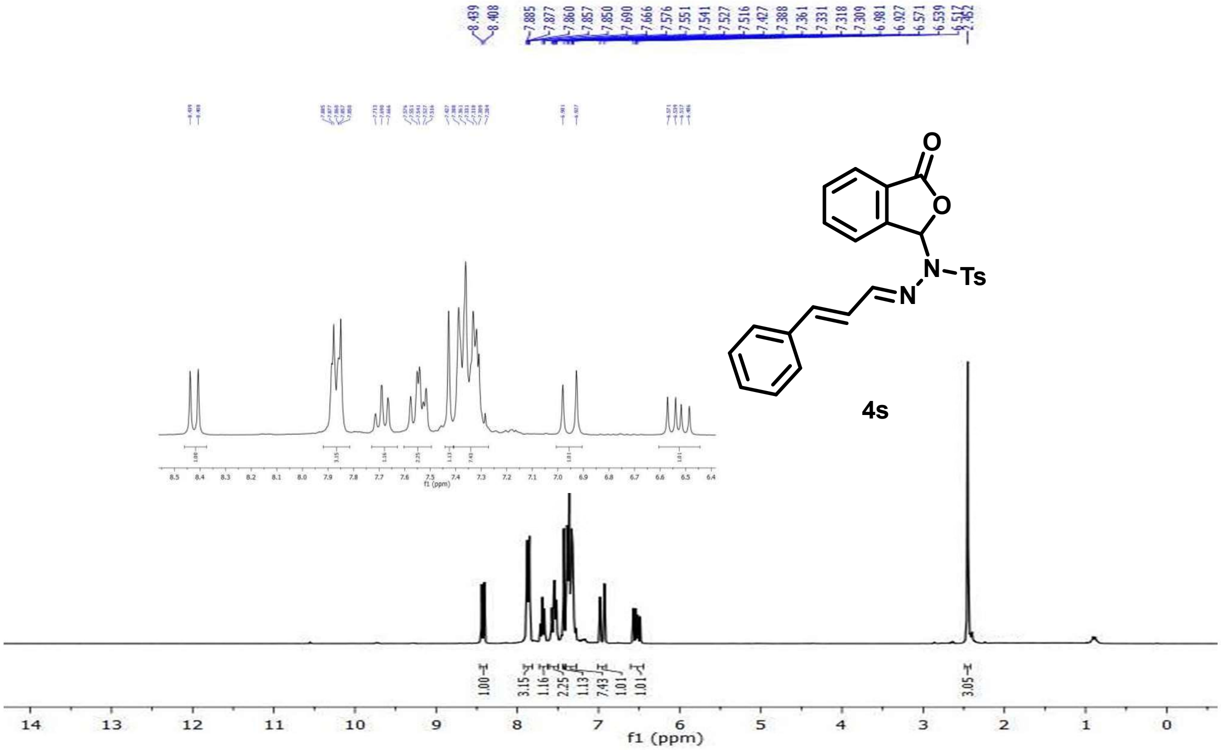

4s

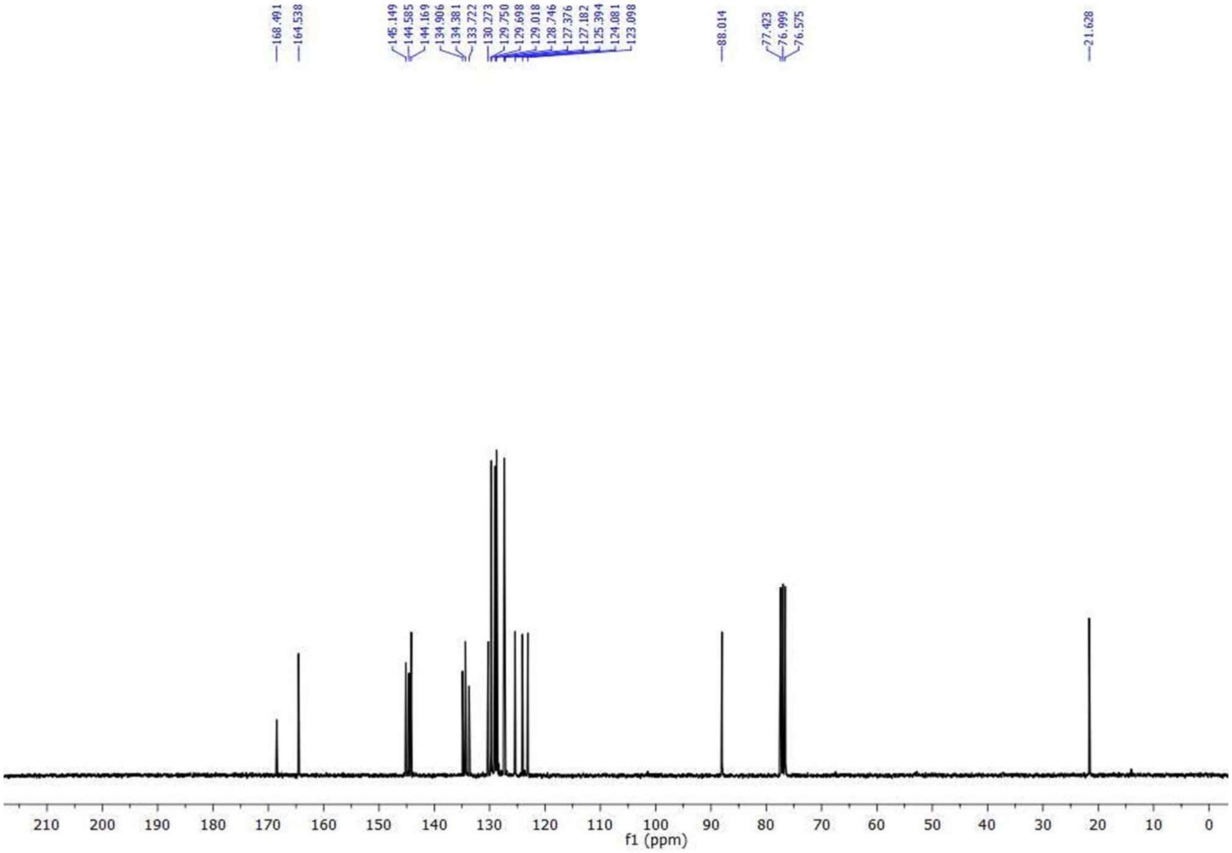

Figure S23. ^{1}H NMR (300 MHz) and ^{13}C NMR (75 MHz) spectra of compound 4t in $CDCl_3$.

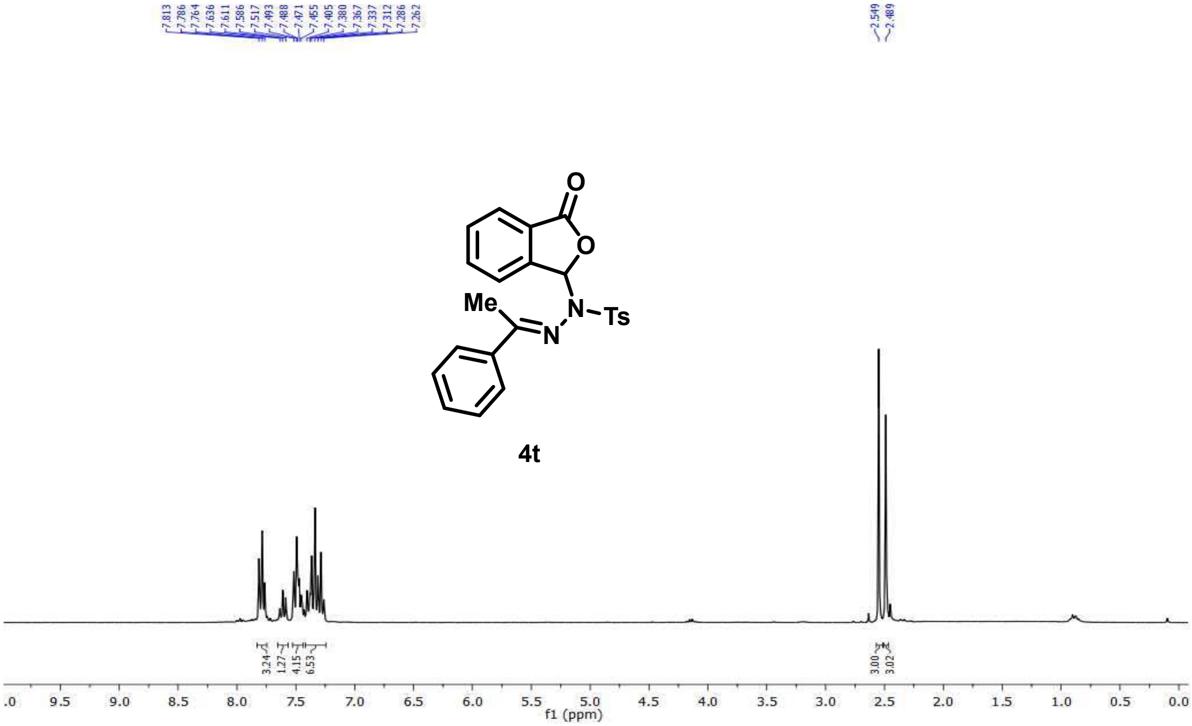
7.813 7.786 7.764 7.636 7.611 7.586 7.517 7.493 7.488 7.471 7.455 7.405 7.380 7.367 7.337 7.312 7.286 7.262
2.549 2.489
Me
N
N
Ts
O
O
4t
3.24 1.27 4.15 6.53
3.00 3.02
f1 (ppm)

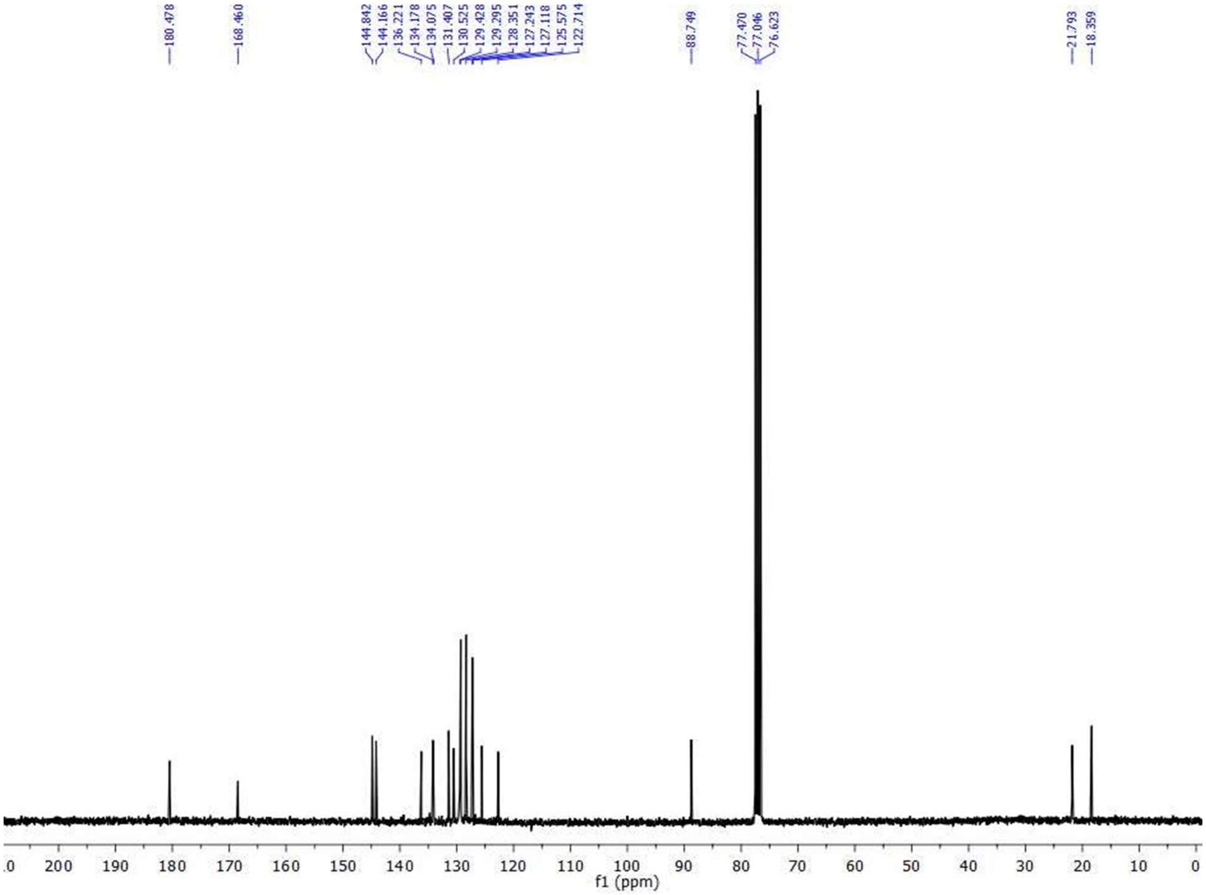
180.478
168.460
144.842 144.166 136.221 134.178 134.075 131.407 130.525 129.428 129.295 128.351 127.243 127.118 125.575 122.714
88.749
77.470 77.046 76.623
21.793 18.359
f1 (ppm)

Figure S24. ^{1}H **NMR (300 MHz) and** ^{13}C **NMR (75 MHz) spectra of compound 4u in** $CDCl_3$.

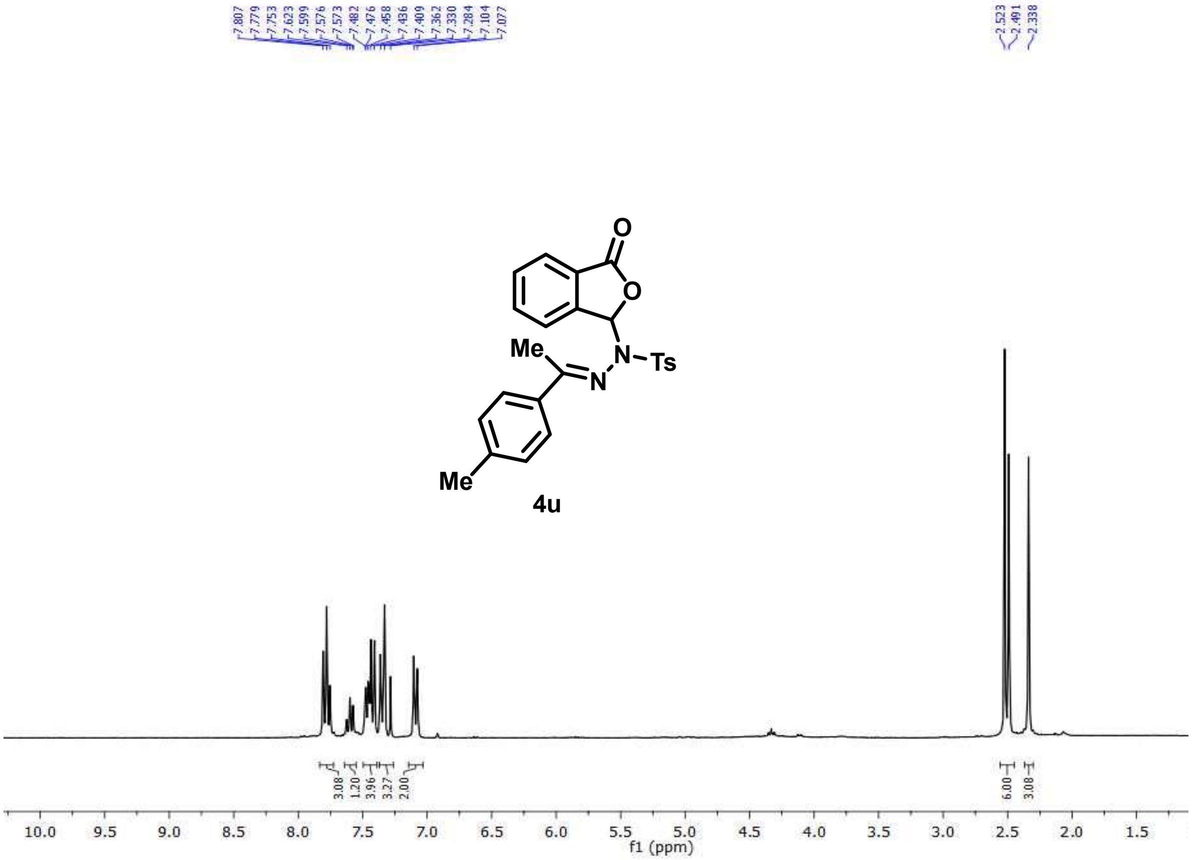

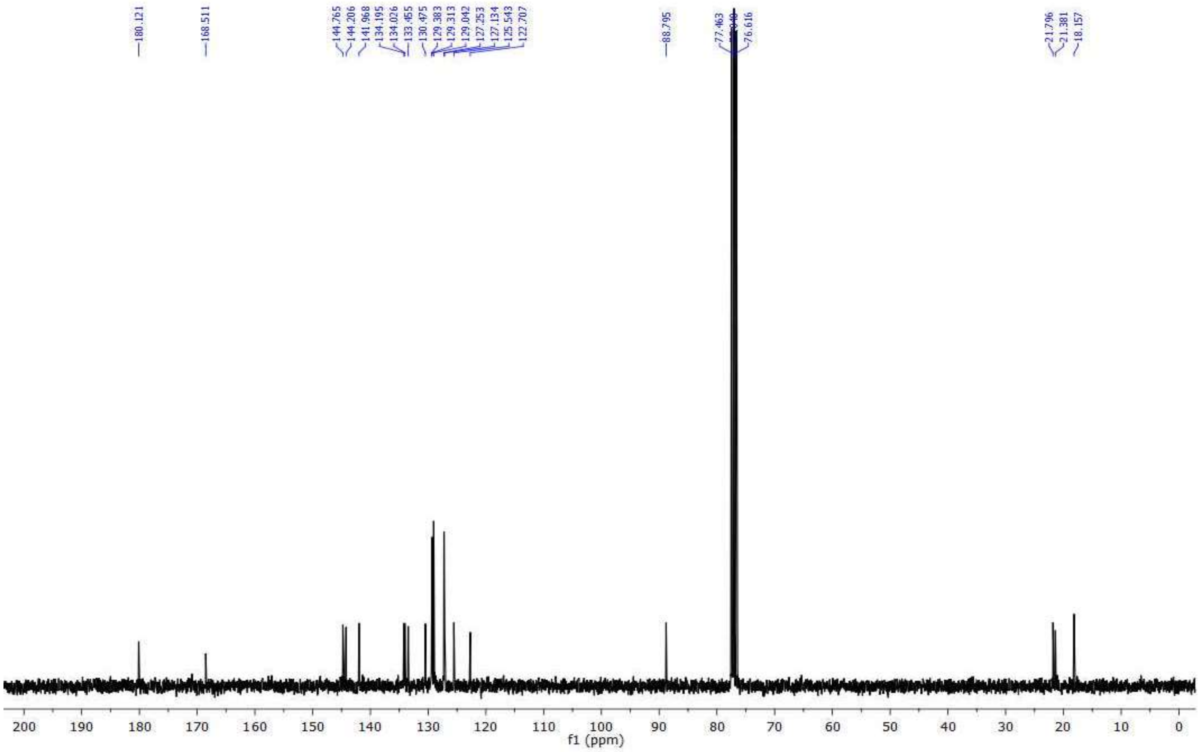

Figure S25. ^{1}H NMR (300 MHz) and ^{13}C NMR (75 MHz) spectra of compound **4v** in $CDCl_3$.

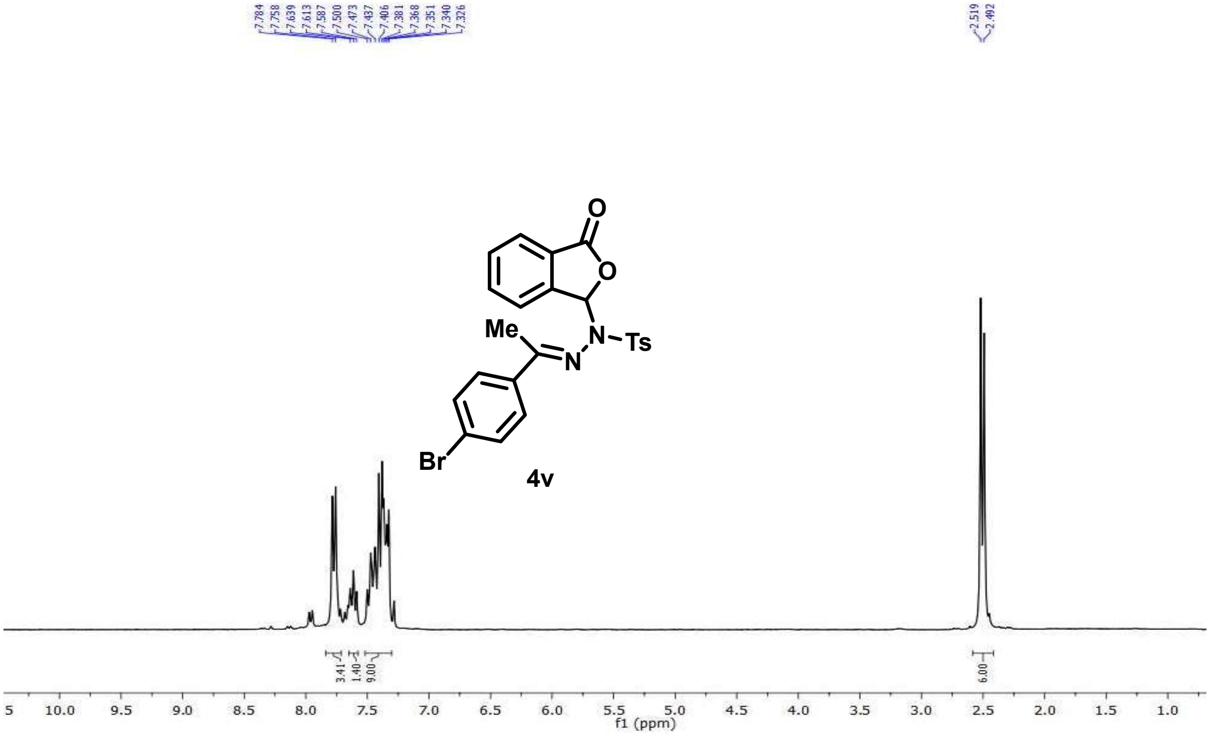

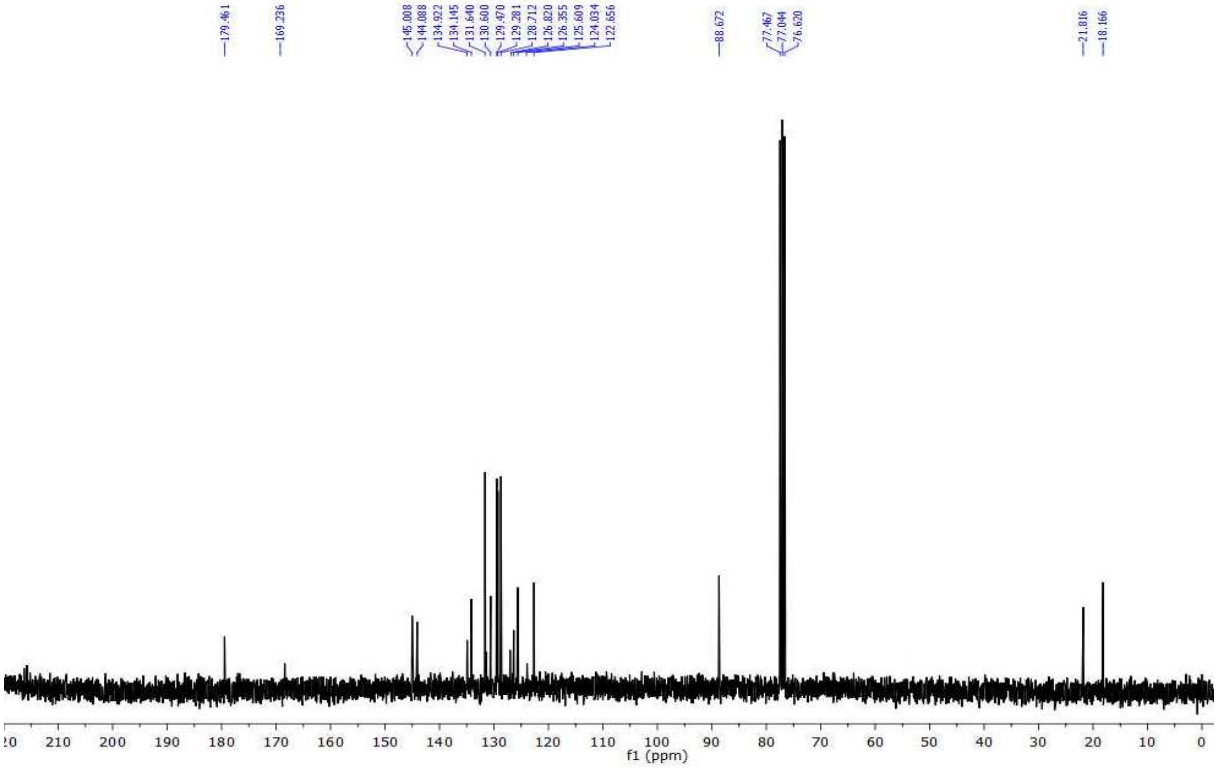

Figure S26. ^{1}H NMR (300 MHz) and ^{13}C NMR (75 MHz) spectra of compound **4w** in $CDCl_3$.

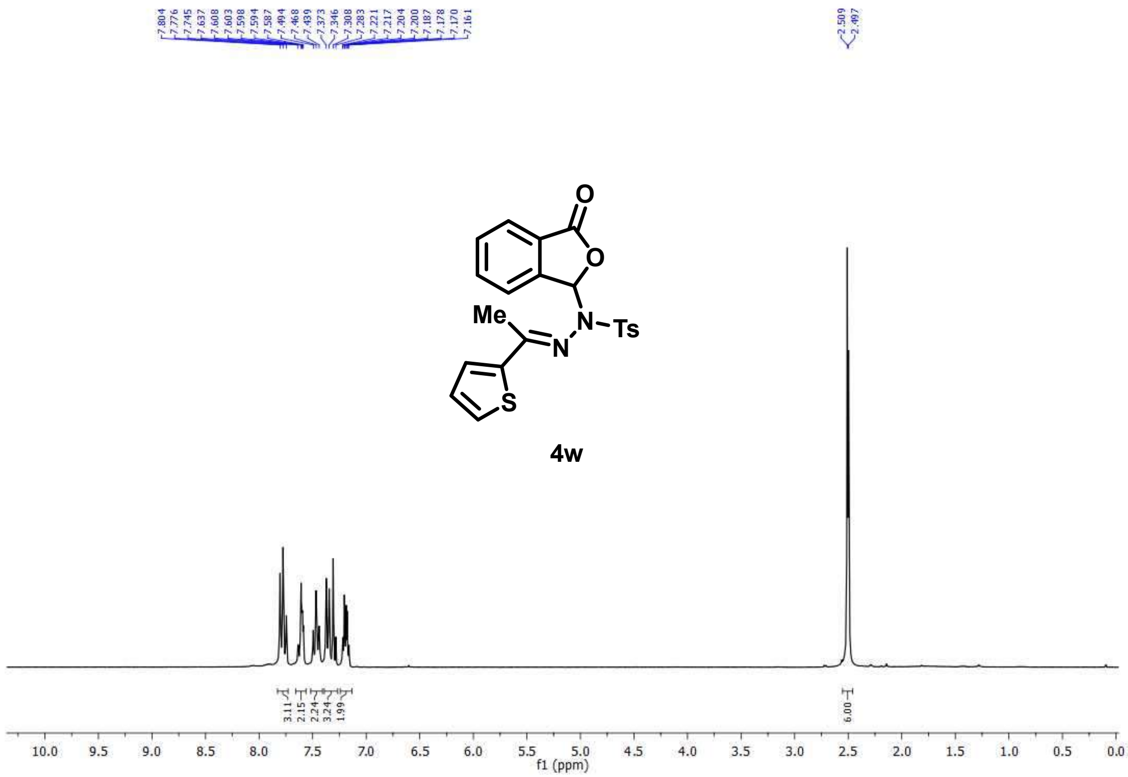

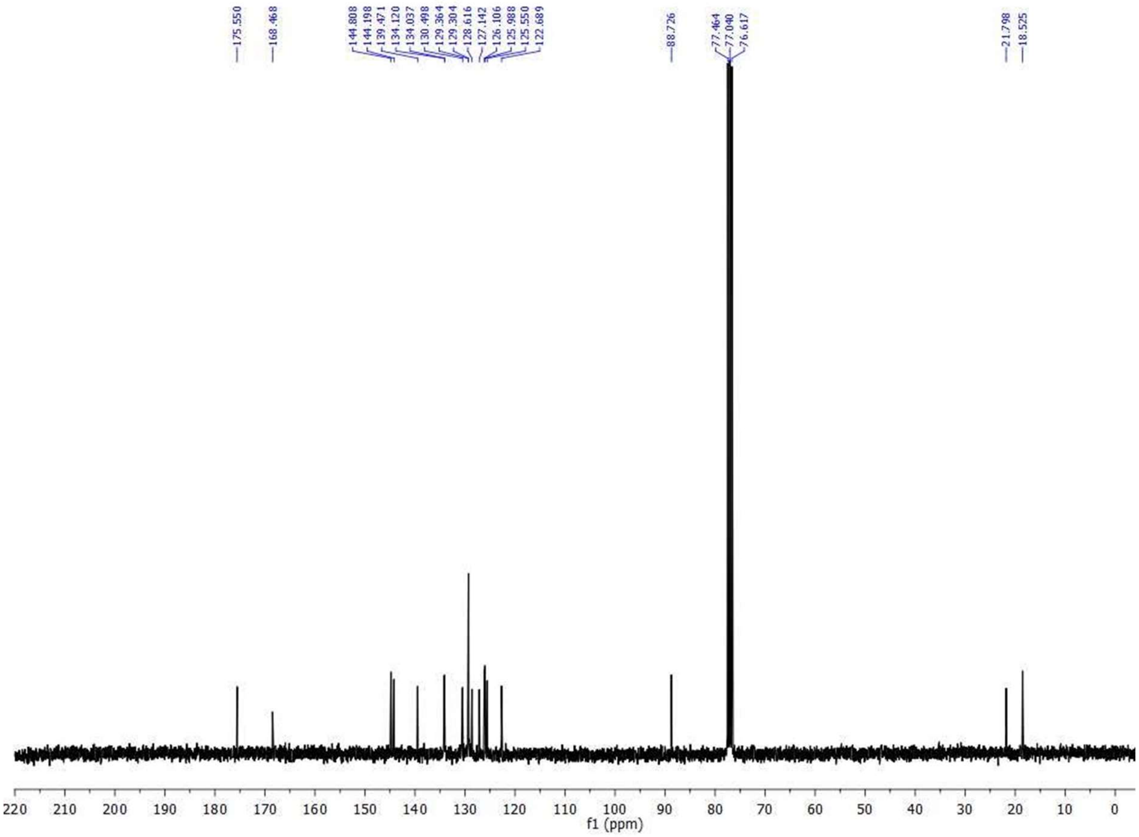

Figure S27. ^{1}H NMR (300 MHz) and ^{13}C NMR (75 MHz) spectra of compound 4x in $CDCl_3$.

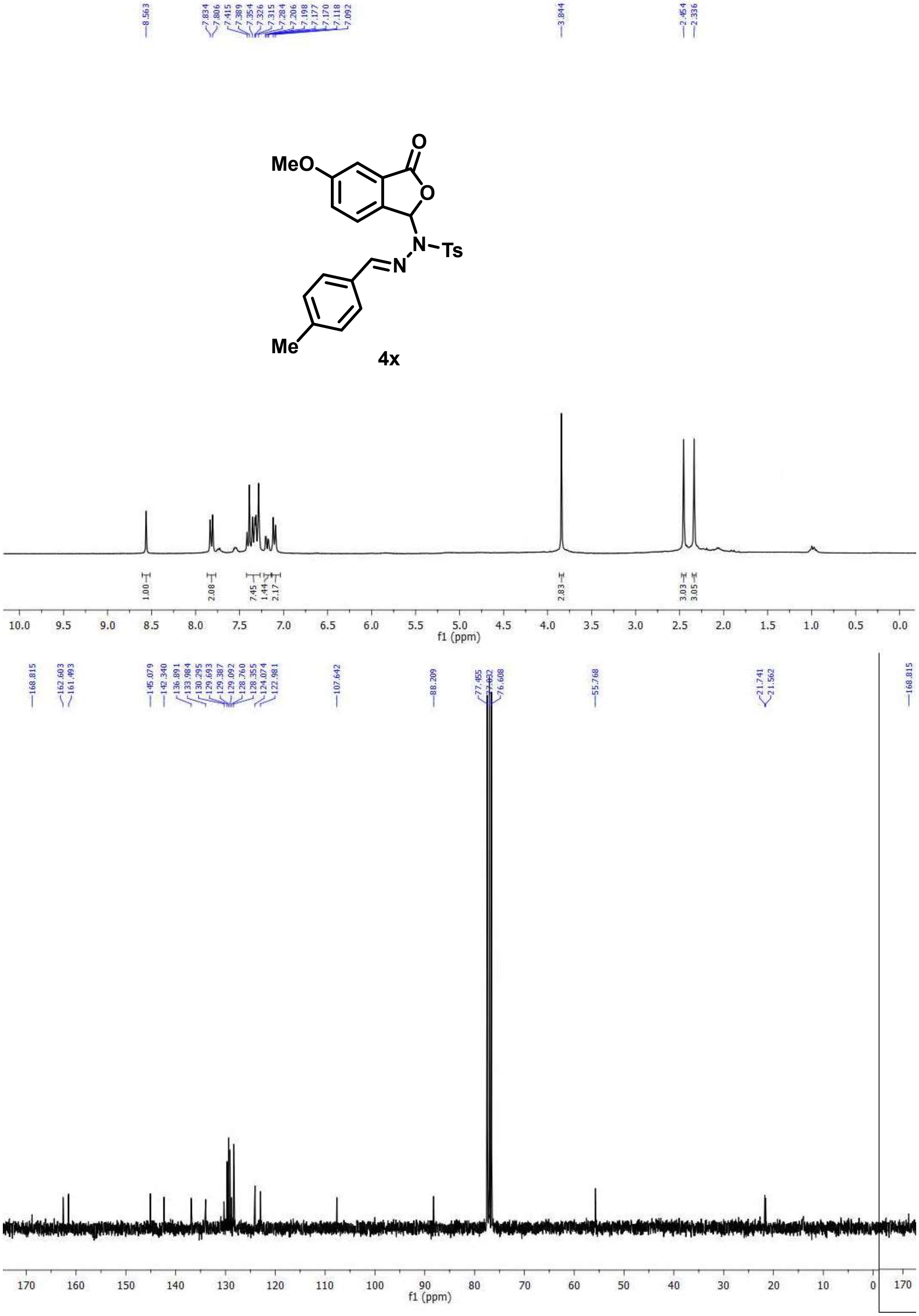

Figure S28. ^{1}H NMR (300 MHz) and ^{13}C NMR (75 MHz) spectra of compound 1b in $CDCl_3$.

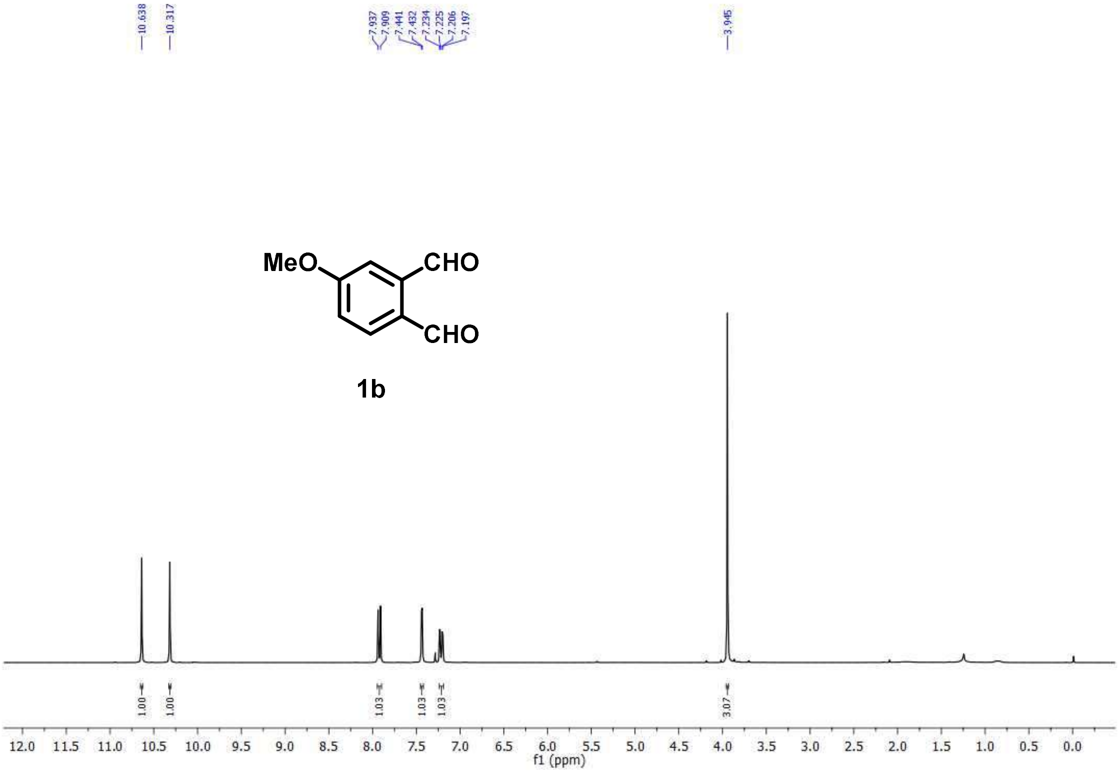
10.638
10.317
7.937
7.909
7.441
7.432
7.234
7.225
7.206
7.197
3.945
MeO
CHO
CHO
1b
1.00
1.00
1.03
1.03
1.03
3.07
f1 (ppm)

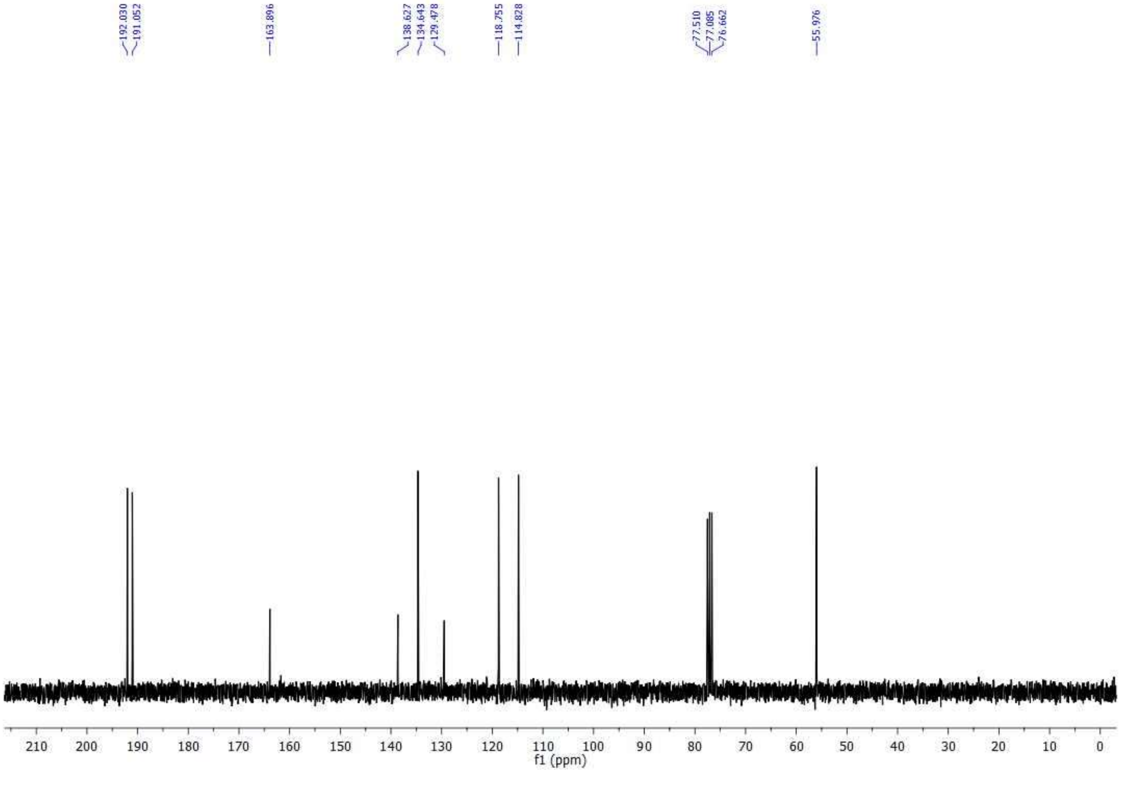
192.030
191.052
163.896
138.627
134.643
129.478
118.755
114.828
77.510
77.085
76.662
55.976
f1 (ppm)

3. X-ray crystallographic characterization data of compound 4m

Figure S29. ORTEP diagram of compound 4m (CCDC 2080548). Ellipsoids are drawn at 50% probability level.

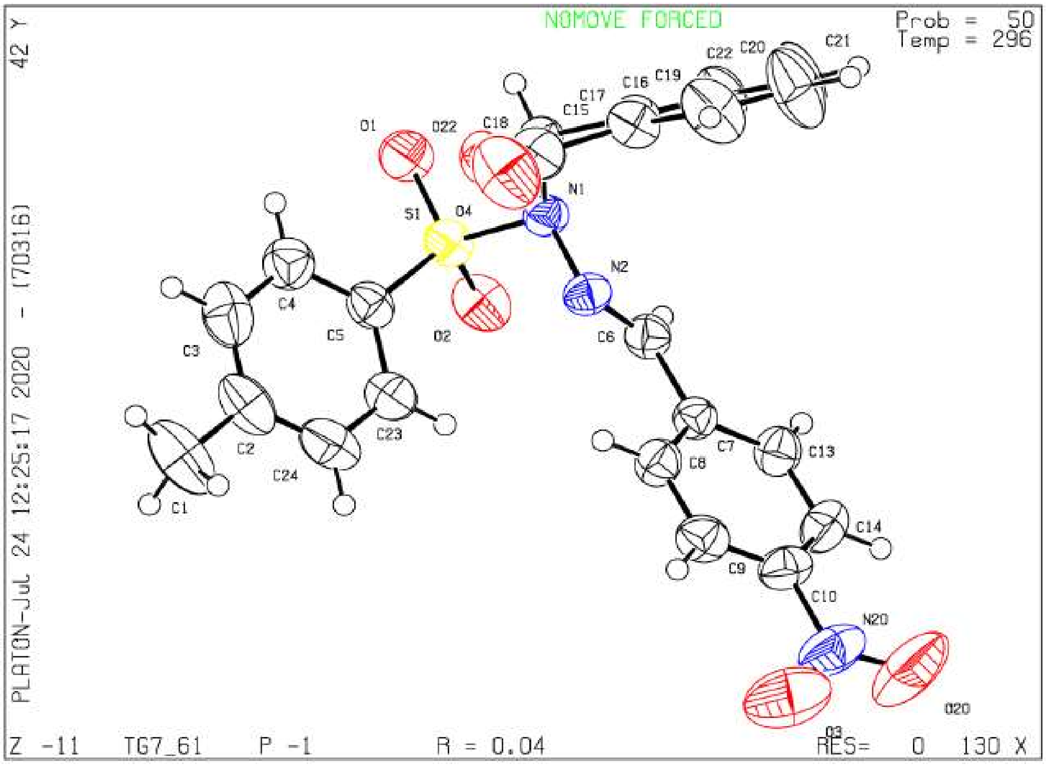

Table S1: Crystal data and structure refinement for 4m (CCDC 2080548):

Datablock: TG7_61

Bond precision:	C-C = 0.0033 A		Wavelength=0.71073
Cell:	a=10.3641(4)	b=10.3966(4)	c=11.5337(4)
	alpha=110.628(2)	beta=106.297(2)	gamma=95.635(2)
Temperature:	296 K		

	Calculated	Reported
Volume	1089.17(7)	1089.17(7)
Space group	P -1	P -1
Hall group	-P 1	-P 1
Moiety formula	C22 H17 N3 O6 S	C22 H17 N3 O6 S
Sum formula	C22 H17 N3 O6 S	C22 H17 N3 O6 S
Mr	451.45	451.45
Dx,g cm-3	1.377	1.377
Z	2	2
Mu (mm-1)	0.193	0.193
F000	468.0	468.0
F000'	468.48	
h,k,lmax	12,12,14	12,12,14
Nref	4326	4296
Tmin,Tmax	0.950,0.970	0.865,0.990
Tmin'	0.947	

Correction method= # Reported T Limits: Tmin=0.865 Tmax=0.990
AbsCorr = MULTI-SCAN

Data completeness= 0.993 Theta(max)= 26.078

R(reflections)= 0.0422(3653) wR2(reflections)= 0.1321(4296)

S = 1.024 Npar= 290

Crystal structure experimental protocol.

Single crystal of compound **4m** was mounted on a Bruker's Kappa Apex II CCCD **2080548** diffractometer equipped with a graphite monochromator and MoKα radiation at the temperature of 296 K. All other crystallographic details such as h, k, l ranges, 2θ ranges, and R-factors can be found above (Table S1).

4. Computational analysis of reaction pathway and study of bioactivity

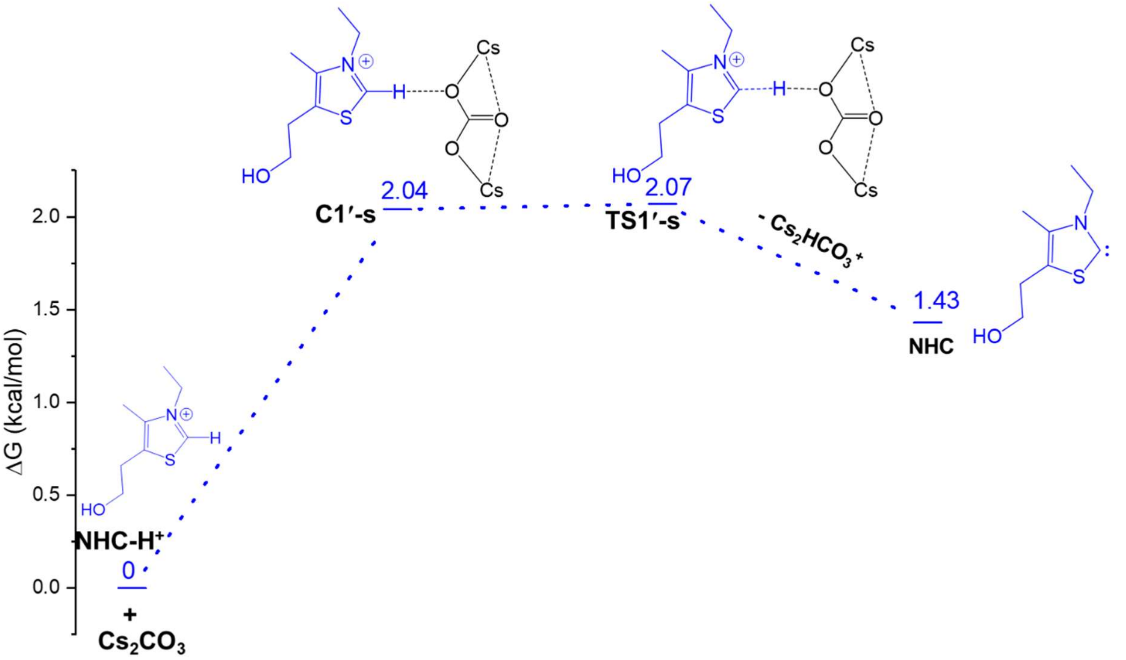

Figure S30. Free energy diagram for forming the **NHC** carbene from azolium cation with base CS_2CO_3.

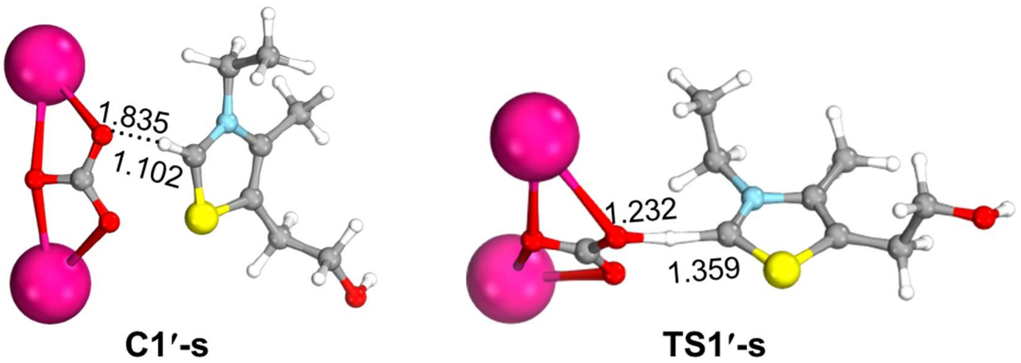

Figure S31. The optimized geometries of stationary points for forming the **NHC** from azolium cation with base CS_2CO_3. The distances are given in Å. [C: grey, O: red, S: yellow, H: white, N: light blue, Cs: pink].

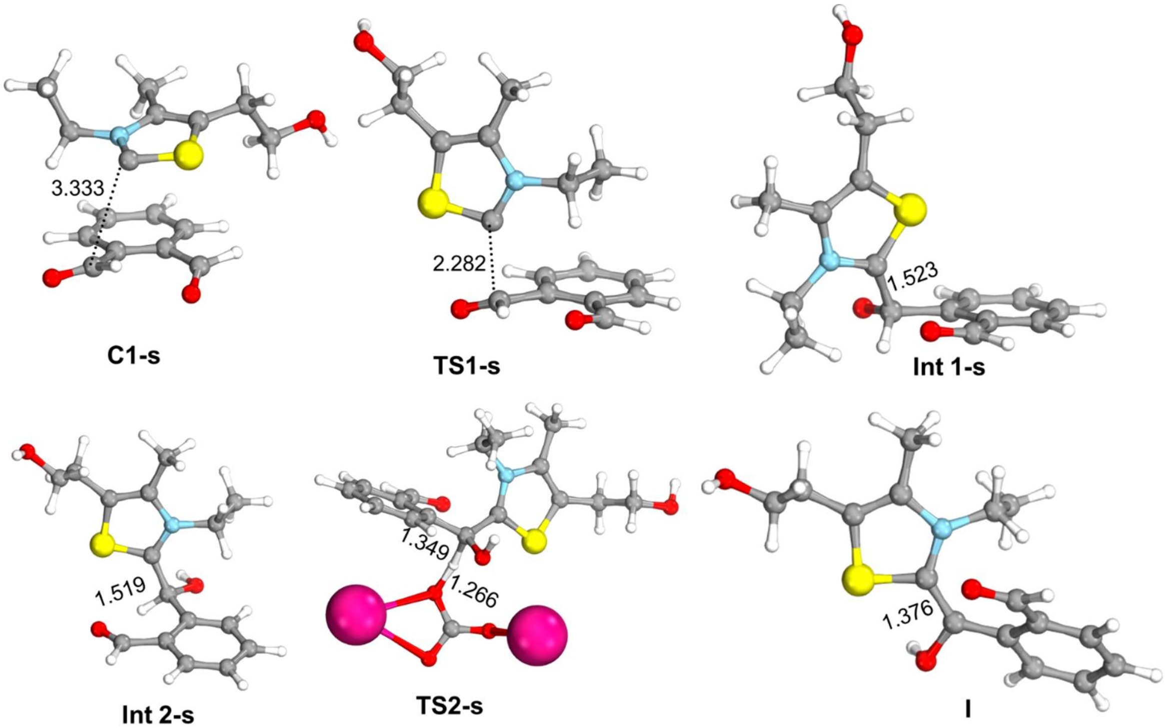

Figure S32. The optimized geometries of stationary points for forming the enolate Breslow intermediate from **NHC** and **phthalaldehyde**. The distances are given in Å. [C: grey, O: red, S: yellow, H: white, N: light blue, Cs: pink].

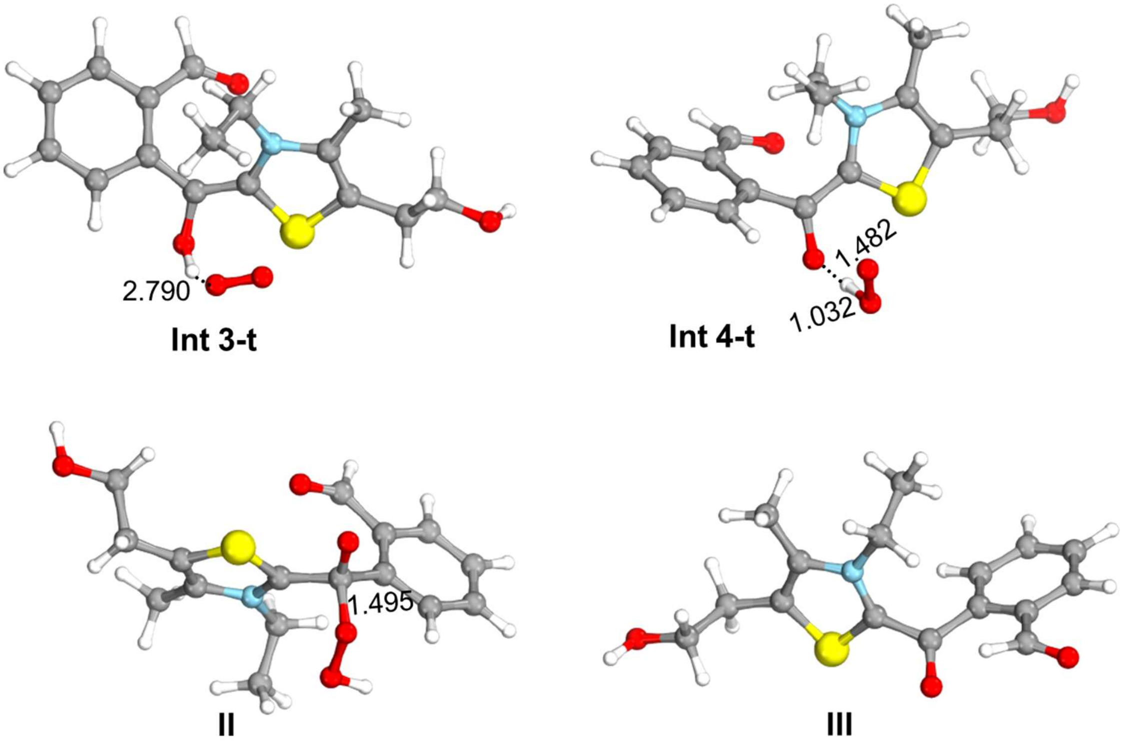

Figure S33. The optimized geometries of stationary points for forming aerial oxidation pathway to generate acyl azolium intermediate (**III**). The distances are given in Å. [C: grey, O: red, S: yellow, H: white, N: light blue].

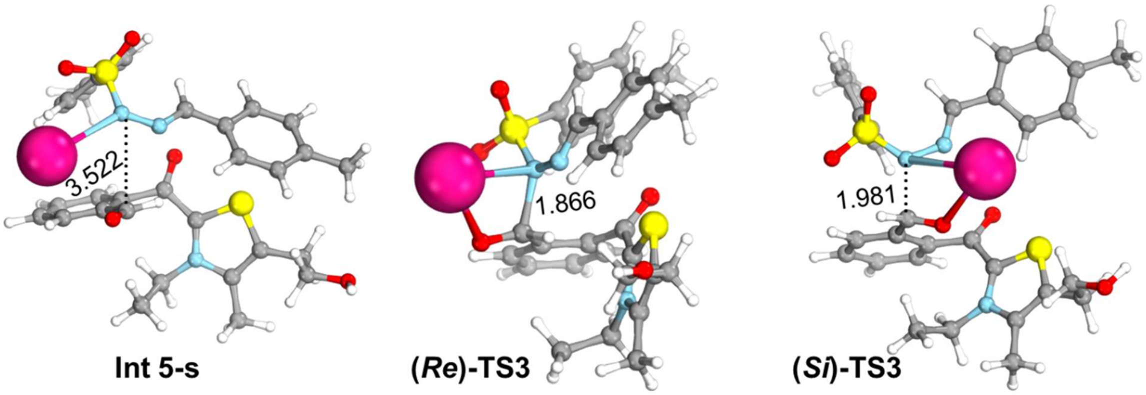

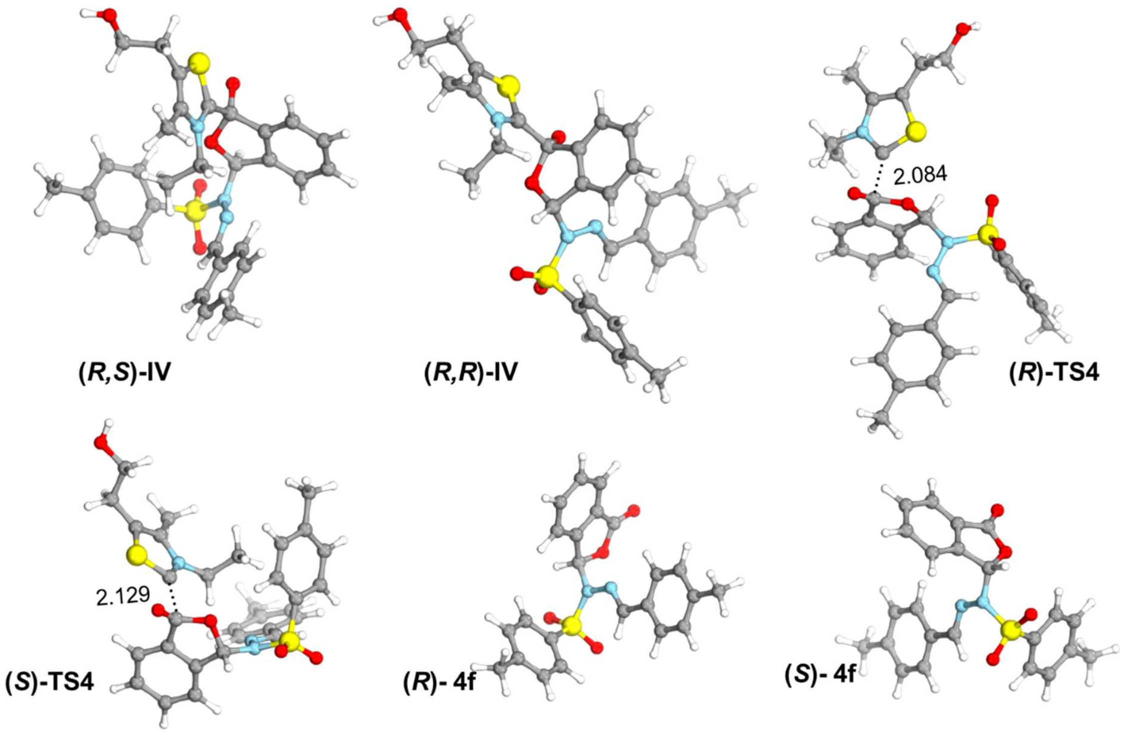

Figure S34. The optimized geometries of stationary points for forming the final product hemiaminal ester along with regeneration of the active catalyst. The distances are given in Å. [C: grey, O: red, S: yellow, H: white, N: blue, Cs: pink].

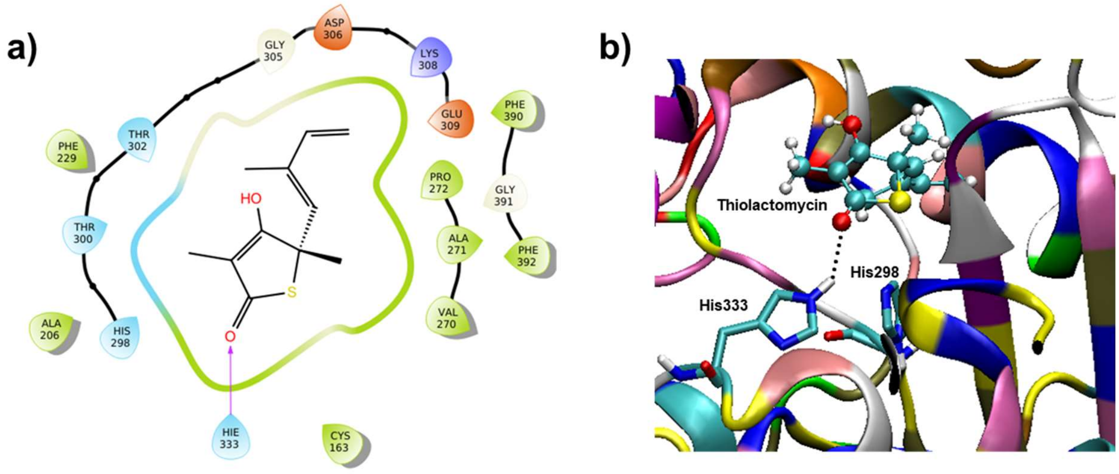

Figure S35. Glide molecular docking interactions of receptor (PDB ID: 1FJ4) with Thiolactomycin (TLM). a) Protein-ligand schematic interaction diagram of the protein and TLM complex, b) Binding pose of TLM in the active site of the receptor.

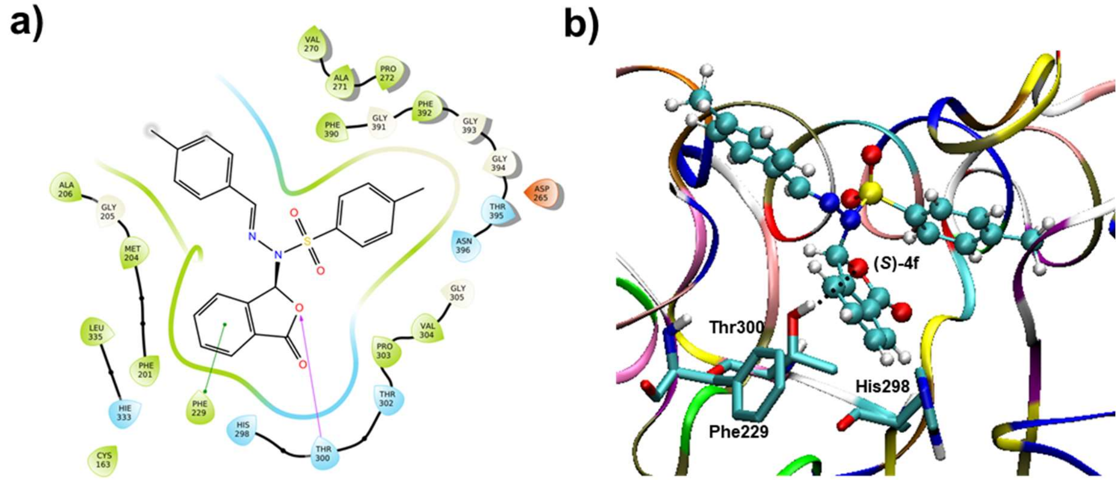

Figure S36. Glide molecular docking interactions of receptor (PDB ID: 1FJ4) with **(*S*)-4f.** a) Protein-ligand schematic interaction diagram of the protein and **(*S*)-4f** complex, b) Binding pose of **(*S*)-4f** in the active site of the receptor.
